# Paroxetine repurposing enhances antitumor immunity via SPOP-mediated PD-L1 ubiquitination and proteasomal degradation

**DOI:** 10.1186/s13046-026-03648-z

**Published:** 2026-01-27

**Authors:** Mengting Xu, Saisai Tian, Hanchi Xu, Xinying Xue, Qing Zhang, Hongmei Hu, Gaosong Wu, Xiangxin Geng, Dianping Yu, Hanchen Xu, Mei Xie, Linyang Li, Xinru Li, Simeng Li, Shize Xie, Xuwen Lin, Shuzhen Lyu, Yutong Xie, Biao Zhang, Haiyang Zhou, Qun Wang, Weidong Zhang, Sanhong Liu

**Affiliations:** 1 State Key Laboratory of Discovery and Utilization of Functional Components in Traditional Chinese Medicine, Shanghai Frontiers Science Center of TCM Chemical Biology, Institute of Interdisciplinary Integrative Medicine Research, Shanghai University of Traditional Chinese Medicine, 1200 Cailun Road, Shanghai, 201203 China; 2 Department of Phytochemistry, School of Pharmacy, Second Military Medical University, 325 Guohe Road, Shanghai, 200433 China; 3 Department of Respiratory and Critical Care, Emergency and Critical Care Medical Center, Beijing Shijitan Hospital, Capital Medical University, Beijing, China; 4 Institute of Digestive Diseases, Longhua Hospital, Shanghai University of Traditional Chinese Medicine, Shanghai, China; 5 Department of Breast Surgery, Beijing Shijitan Hospital, Capital Medical University, Beijing, China; 6 Department of Pathology, Chinese PLA General Hospital, the First Medical Centre, Beijing, China; 7 Shanghai Hui Tian Jin Ze Biomedical Technology Co., Ltd, No. 888, Huanhu West Second Road, Shanghai, 201236 China; 8 Division of Colorectal Surgery, Changzheng Hospital, No. 415, Fengyang Road, Shanghai, 200003 China; 9 State Key Laboratory for Quality Ensurance and Sustainable Use of Dao-di Herbs, Institute of Medicinal Plant Development, Chinese Academy of Medical Sciences and Peking Union Medical College, Beijing, 100193 China; 10 State Key Laboratory of Drug Research, Shanghai Institute of Materia Medica, Chinese Academy of Sciences, Shanghai, 201203 China

**Keywords:** Paroxetine, PD-L1, SPOP, Cancer, Ubiquitination

## Abstract

**Background:**

Immunotherapy targeting the PD-1/PD-L1 axis shows promise in colon and lung cancer treatment but faces challenges like high costs, low response rates, and drug resistance. Developing new small molecule inhibitors is complex. Repurposing existing drugs offers advantages, and paroxetine (PAR), an FDA approved antidepressant, has shown potential antitumor effects, yet its role as an immune checkpoint inhibitor is unclear.

**Methods:**

In this study, we investigated PAR as an immune checkpoint inhibitor. We used various cell lines, including colon and lung cancer cells, and in vivo mouse models. Techniques such as Western blotting, flow cytometry, immunofluorescence, and immunohistochemistry were employed to analyze protein expression, cell surface marker levels, and immune cell populations. We also conducted gene knockdown and overexpression experiments, as well as molecular docking and binding assays.

**Results:**

PAR downregulates PD-L1 protein levels in a concentration and time dependent manner in multiple cancer cell lines. In vivo, it inhibits tumor growth in colon and lung cancer mouse models by activating T cell immunity. Mechanistically, PAR binds to the Asp130 site of speckle-type POZ protein (SPOP), stabilizing this E3 ubiquitin ligase to promote PD-L1 ubiquitination and proteasomal degradation. Moreover, PAR combines with an anti-CTLA4 antibody enhances cancer cell inhibition, and it also suppresses AOM/DSS induced colon cancer.

**Conclusions:**

Our findings demonstrate that PAR can function as an immune checkpoint inhibitor by targeting SPOP to degrade PD-L1, enhancing antitumor immunity. This provides a new theoretical basis for using PAR in colorectal and lung cancer treatment and offers insights into repurposing other drugs for cancer therapy.

**Graphical Abstract:**

**Figure:**
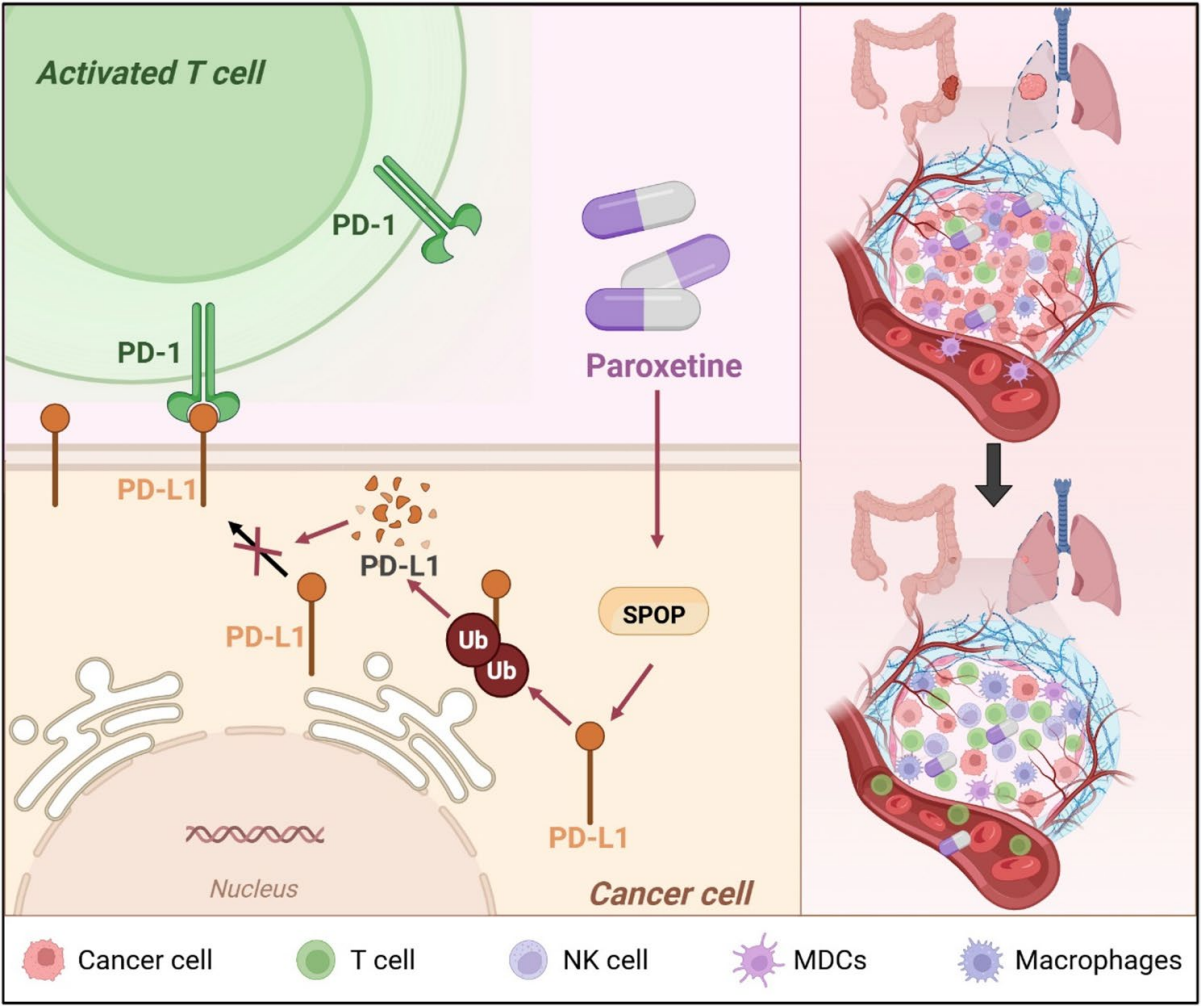
Paroxetine enhances the degradation of PD-L1 by stabilizing the E3 ubiquitin ligase SPOP, thereby transforming the tumor microenvironment from a suppressive state to an activated immune state, which exerts therapeutic effects against colon and lung cancers. This process increases the infiltration and activity of T cells within the tumor microenvironment, enhancing the overall antitumor immune response.

**Supplementary Information:**

The online version contains supplementary material available at 10.1186/s13046-026-03648-z.

## Statement of translational relevance

This study holds substantial translational potential by bridging preclinical findings with actionable clinical applications. Paroxetine (PAR), an FDA-approved agent with established safety profiles, emerges as a repurposable therapeutic that targets the SPOP-PD-L1 axis to modulate antitumor immunity, offering a cost-effective, orally bioavailable alternative to monoclonal antibody-based PD-1/PD-L1 inhibitors. Its ability to enhance tissue penetration and overcome resistance mechanisms addresses critical limitations of current immunotherapies, supporting its rapid evaluation in clinical trials-particularly for patients with limited access to expensive biologics or those refractory to antibody treatments. The observed synergy between PAR and anti-CTLA4 therapy provides a rational basis for combinatorial strategies, which can be readily tested in clinical settings to improve treatment outcomes. Additionally, the negative correlation between SPOP and PD-L1 in human tumors, coupled with the predictive value of SPOP expression for immunotherapy response, enables the development of biomarker-driven patient stratification, ensuring targeted administration of PAR or related therapies to those most likely to benefit. Collectively, these insights accelerate the translation of basic mechanistic discoveries into practical interventions, potentially expanding therapeutic options, reducing healthcare costs, and optimizing personalized cancer immunotherapy.

## Introduction

In the field of tumor immunology, the PD-1/PD-L1 signaling axis occupies a central role in mediating tumor cell evasion of immune surveillance and resistance to immune-mediated attack. Tumor cells frequently upregulate PD-L1 expression, and this elevated PD-L1 engages PD-1 receptors on T cells, resulting in impairment of T cell effector function and a marked reduction in their capacity to eliminate tumor cells [1–5]. Accordingly, the development of PD-1 and PD-L1 inhibitors has emerged as a cornerstone of modern cancer immunotherapy [6]. These inhibitors disrupt the immunosuppressive interaction between PD-L1 and PD-1, thereby reinvigorating T cell-mediated antitumor immune responses. Clinically, immune checkpoint inhibitors such as pembrolizumab and nivolumab have demonstrated substantial efficacy in patients with microsatellite instability-high (MSI-H) and mismatch repair-deficient (dMMR) colorectal cancer, and are established as first-line therapies for advanced non-small cell lung cancer (NSCLC) [7–11]. While these agents counteract tumor immune evasion mechanisms to potentiate the host antitumor immune response, their clinical utility is hampered by challenges including drug resistance, risk of cancer recurrence, and interpatient variability in response-attributed to factors such as genetic background, age, and sex. Furthermore, the complex tumor microenvironment, characterized by an abundance of immunosuppressive cells, cytokine imbalances, and active angiogenesis, further diminishes the efficacy of these immunotherapies [12]. Thus, there is an urgent need for innovative therapeutic strategies.

In contrast to PD-1/PD-L1 monoclonal antibodies, small-molecule inhibitors targeting this pathway are relatively understudied with sparse clinical trial data, posing substantial challenges in drug design and high-throughput screening [13]. The development of potent, low-toxicity small-molecule inhibitors of PD-1 and PD-L1 remains particularly arduous [14]. In this context, drug repurposing of approved agents offers distinct advantages, including accelerated clinical translation and efficacy evaluation. This approach is less time-consuming and cost-intensive, leveraging the extensive clinical experience with existing drugs to provide a well-characterized profile of their therapeutic effects, adverse reactions, and safety profiles.

Paroxetine (PAR), an FDA-approved selective serotonin reuptake inhibitor (SSRI), is primarily indicated for the management of depression, anxiety disorders, and obsessive-compulsive disorder [15]. Emerging evidence suggests that PAR harbors promising antitumor properties: it has been shown to suppress cancer cell proliferation through cell cycle arrest and induction of apoptosis [16]. Additionally, as a GRK2 inhibitor, PAR enhances S1PR1 expression on T cells, promoting T cell egress from the bone marrow and facilitating their infiltration into glioblastoma to exert antitumor effects [17, 18]. Nevertheless, the potential of PAR to function as an immune checkpoint inhibitor remains largely uninvestigated.

In this study, we demonstrate that PAR specifically targets the SPOP, a mechanism that facilitates PD-L1 ubiquitination and subsequent degradation, thereby enhancing antitumor immunity in vivo. Our findings not only provide a novel mechanistic basis for the application of PAR in the treatment of colorectal and lung cancers but also offer new insights into the repurposing of other clinically available drugs.

## Materials and methods

### Cells and reagents

Human colorectal cancer cells (RKO, HCT116, HT29, and DLD1 in MEM, 5 A, and 1640 media, respectively), human lung cancer cells (H1975 and H460 in DMEM), mouse colorectal cancer cells (MC38 in DMEM), mouse lung cancer cells (Lewis in 1640), human gastric cancer cells (AGS in F-12 K), and human anaplastic large cell lymphoma cells (Karpas-299 in 1640) were sourced from the Shanghai Institute of Cell Biology, Chinese Academy of Sciences. Jurkat cells overexpressing PD-1 were provided by Kongming Wu’s research group (Department of Oncology, Tongji Hospital, Tongji Medical College, Huazhong University of Science and Technology, Wuhan, China). All culture media (Meron Bio, China) were supplemented with 100 mg/mL streptomycin, 100 U/mL penicillin, and 10% fetal bovine serum (Biological Industries, Cromwell, CT, USA). Paroxetine hydrochloride (PAR), Fluoxetine hydrochloride (Fux), chloroquine (CQ), primaquine, MG-132, cycloheximide (CHX), and 3-methyladenine (3-MA) were obtained from MedChemExpress. In addition, the relevant antibodies used in the experiments are listed in Supplementary Table S1.

### Cell viability and toxicity assays

The effects of different PAR concentrations on cell viability and cell proliferation were evaluated via the Cell Counting Kit-8 (CCK-8) and Cell Proliferation Kit (Beyotime, Haimen, China) according to the manufacturers’ instructions.

### Western blotting and flow cytometry

Six-well plates were inoculated with the appropriate cell density. After the cells expanded and were treated with drugs, all the cells were collected. Total protein was extracted via NP40 lysis buffer supplemented with 1% protease inhibitor (Beyotime, Haimen, China). The protein concentration was determined with a BCA protein assay kit (Beo Tianmei, Haimen, China). The proteins were separated by SDS-PAGE and transferred to a PVDF membrane. The membranes were blocked with 5% skim milk and incubated with specific antibodies overnight at 4 °C, followed by a 1-hour incubation with secondary antibodies at room temperature. The results were scanned via a Bio-Rad imaging system. To assess PD-L1 abundance on cell membranes via flow cytometry, drug-treated cells were incubated with an anti-PD-L1 antibody at 4 °C for 30 min, washed with PBS and resuspended in 500 µL of PBS.

### Immunofluorescence

The cells were inoculated into 12-well plates and treated with PAR for 24 h. After the samples were washed twice with PBS, they were fixed with paraformaldehyde for 15 min, followed by three 5-minute washes with PBS. The cells were then blocked with 5% BSA for one hour. A specific conjugate antibody was incubated overnight at 4 °C, followed by three washes with TBST. Next, the membrane was incubated with the secondary antibody at room temperature in the dark for one hour and then washed three times with TBST. Finally, DAPI-containing antifade mounting medium (Beyotime, Haimen, China) was added, and images were captured using Cytation 5 (BioTek, USA).

## Transfection and Immunoprecipitation

siRNAs targeting PD-L1, MARCH8, HRD1, SPOP, ARIH1, STUB1, and BTRC genes, as well as the overexpression plasmids pcDNA3.1-Ub, pcDNA3.1-USP22, and pcDNA3.1-OTUB1, were obtained from GenePharma (Shanghai, China). These siRNAs and plasmids were transfected into RKO cells via Lipofectamine 2000 (Invitrogen, Carlsbad, CA) for at least 6 h, followed by 48 h of culture before drug treatment. The relevant siRNA sequences are provided in Supplementary Table 2. For immunoprecipitation experiments [19], cells that overexpressed NC or Ub were lysed via IP cell lysis buffer (Beyotime, Haimen, China) containing 1% protease inhibitor. The lysates were then incubated overnight with anti-FLAG beads at 4 °C on a shaker. After several washes, Western blotting analysis was performed.

### RT-qPCR analysis

Reagents were purchased from Takara (Dalian, China), and total cellular RNA was extracted via RNAiso-Plus. The Prime Script RT Kit was subsequently used to transcribe mRNA into cDNA according to the manufacturer’s guidelines. RT-qPCR analyses were conducted on a LightCycler^®^ 96 system (Roche, Basel, Switzerland). The primer sequences are shown in Supplementary Table S3.

## Cellular thermal shift assay (CETSA)

The collected RKO cells were first lysed with IP lysis buffer supplemented with 1% protease inhibitor (Beyotime, Haimen, China), and the supernatant was treated with PAR (200 µM) for a period of time, heated with a gradient or constant temperature, and centrifuged to remove the thermally denatured precipitated proteins. The target proteins were subsequently detected and quantified via Western blotting and plotted on a graph.

## Molecular Docking and microscale thermophoresis (MST)

The molecular structures of the PAR compounds were sourced from the PubChem database (https://pubchem.ncbi.nlm.nih.gov/) and imported into Molecular Operating Environment (MOE) software [20]. The crystal structures of SPOP were retrieved from the literature and imported into MOE [21, 22]. Molecular docking calculations were conducted via the DOCK module in MOE, selecting the highest-scoring results for analysis. GFP SPOP and mutant plasmids were constructed on the basis of this analysis and transfected into 293T cells, and cell lysates were prepared via the addition of 1% protease inhibitor (Beyotime, Haimen, China). The lysed protein solutions were then collected and analyzed via MonolithTM NT.115 MST device (NanoTemper, Germany).

### In vitro T cell-mediated killing assay

A 12-well plate was seeded with an appropriate density of cells, which were fully adherent and treated with PAR for 24 h. Jurkat cells stably transfected with human PD-1 and activated with 1 mg/mL phytohemagglutinin (PHA) plus 50 ng/mL phosphatidylinositol 12 myristate 13-acetate (PMA) were introduced for cocultivation, and finally, surviving tumor cells were identified via crystalline violet staining.

### Generation of MC38 PD-L1‑Knockout cell line

To generate a PD-L1-knockout MC38 cell line, gene editing was performed using the CRISPR/Cas9 system. A sgRNA targeting the murine PD-L1 gene (sequence: TATGGCAGCAACGTCACGA) was designed [23], cloned into the BsmBI-digested lentiCRISPRv2 vector, and ligated using T4 DNA ligase. Lentivirus was then packaged in 293T cells using a three-plasmid system (lentiCRISPRv2-sgRNA, pSPAX2, pMD2.G). The collected viral supernatant was used to infect MC38 cells. After 6–8 h of infection, the medium was replaced, and cells stably integrating the vector were selected with puromycin. A monoclonal cell population was subsequently isolated by limiting dilution. Following expansion, the loss of PD-L1 protein expression was confirmed by flow cytometry, thereby successfully establishing the MC38 PD-L1‑KO cell line.

### Animal experiments

Eight-week-old C57BL/6J mice and nude mice were purchased from Shanghai Jihui Laboratory Animal Breeding Co. Tumor models were established via the subcutaneous injection of 8 × 10^6^ MC38 cells and 3 × 10^7^ Lewis cells into the axillary region of mice of the corresponding strains. When the tumor volume of the mice was close to 1600 mm^3^, a portion of the tumor tissue was collected at the end of the experiment and enzymatically digested with collagenase type 4 (1 mg/ml, Yeasen) and DNase 1 (0.1 mg/ml, Yeasen) at 37 °C. The enzymatically digested cells were used to target CD3, CD8, CD25, Gr-1, CD11b, GzmB and Foxp3 for 30 min at 4 °C. The cells were fixed for 30 min before Foxp3 staining, and the samples were analyzed via flow cytometry [24] (Beckman-Coulter, USA). We sent another portion of tumor tissue to three immunohistochemistry companies (Servicebio, Yangming, and Yurun) for immunohistochemical examination, and major organs were harvested for hematoxylin and eosin (H&E) staining.

For the AOM/DSS model, male C57BL/6J mice were injected intraperitoneally with a single dose of AOM (12.5 mg/kg). Seven days later, the mice were given drinking water containing 2.5% DSS for one week. This was followed by a 14-day recovery period with normal drinking water, which was repeated for three cycles [25]. Body weight changes in the treatment group were monitored weekly, and colorectal tissues were collected at the end of the study to assess tumor progression in the different experimental groups. All animal experiments in this study were performed in accordance with the ethical guidelines established by the Department of Laboratory Animal Science, Shanghai University of Traditional Chinese Medicine, with approval numbers PZSHUTCM2311230012, PZSHUTCM2302150001 and PZSHUTCM2305310005.

### Bioinformatics analysis

Gene expression data for COAD, READ, and LUAD were integrated from the TCGA and GTEx databases, and the expression of SPOP was divided into high- and low-expression groups on the basis of the median. We then analyzed the immunological characteristics of the tumor microenvironment, including the activity of immunomodulators, the infiltration levels of tumor-infiltrating immune cells, and the steps of the cancer immunity cycle. In this research, information on 114 immunomodulators was gathered from previous studies [26]. The activities of the cancer immune cycle were quantified via single-sample gene set enrichment analysis (ssGSEA), and the infiltration levels of tumor-infiltrating immune cells were calculated via the TIMER and MCPcounter algorithms [27, 28]. Additionally, immunotherapy cohort data were obtained from the Kaplan-Meier plotter website [29].

### Clinical tissue samples

Human colon cancer tissues and adjacent tissues were obtained from Longhua Hospital of Shanghai University of Traditional Chinese Medicine, whereas human lung cancer tissues and adjacent tissues were obtained from Beijing Shijitan Hospital. Samples from patients with colon cancer and lung cancer treated with neoadjuvant immunotherapy were obtained from Division of Colorectal Surgery, Changzheng Hospital, Shanghai, China and Department of Respiratory and Critical Care, Emergency and Critical Care Medical Center, Beijing Shijitan Hospital, Capital Medical University, Beijing, China, respectively. All human tissue samples were collected in compliance with informed consent policy. The study protocol was approved by the Institutional Review Board of Changzheng Hospital (2023SL043). Specific clinical information is summarized in Tables S4-S7.

### Data analysis

The data that support the findings of this study are available from the corresponding author upon reasonable request. Graphical abstract and animal flow charts were created via Biorender. Quantitative analysis was conducted via ImageJ-win64, and the data were analyzed statistically with GraphPad Prism 10.0.1 and are presented as the mean ± standard error of the mean with independent samples t tests for two-way comparisons and two-way ANOVA for multiple datasets. Bioinformatics analyses were conducted in R version 4.1.1.

## Results

### PAR downregulates PD-L1 and promotes cancer cell killing by T cells

**Fig. 1:**
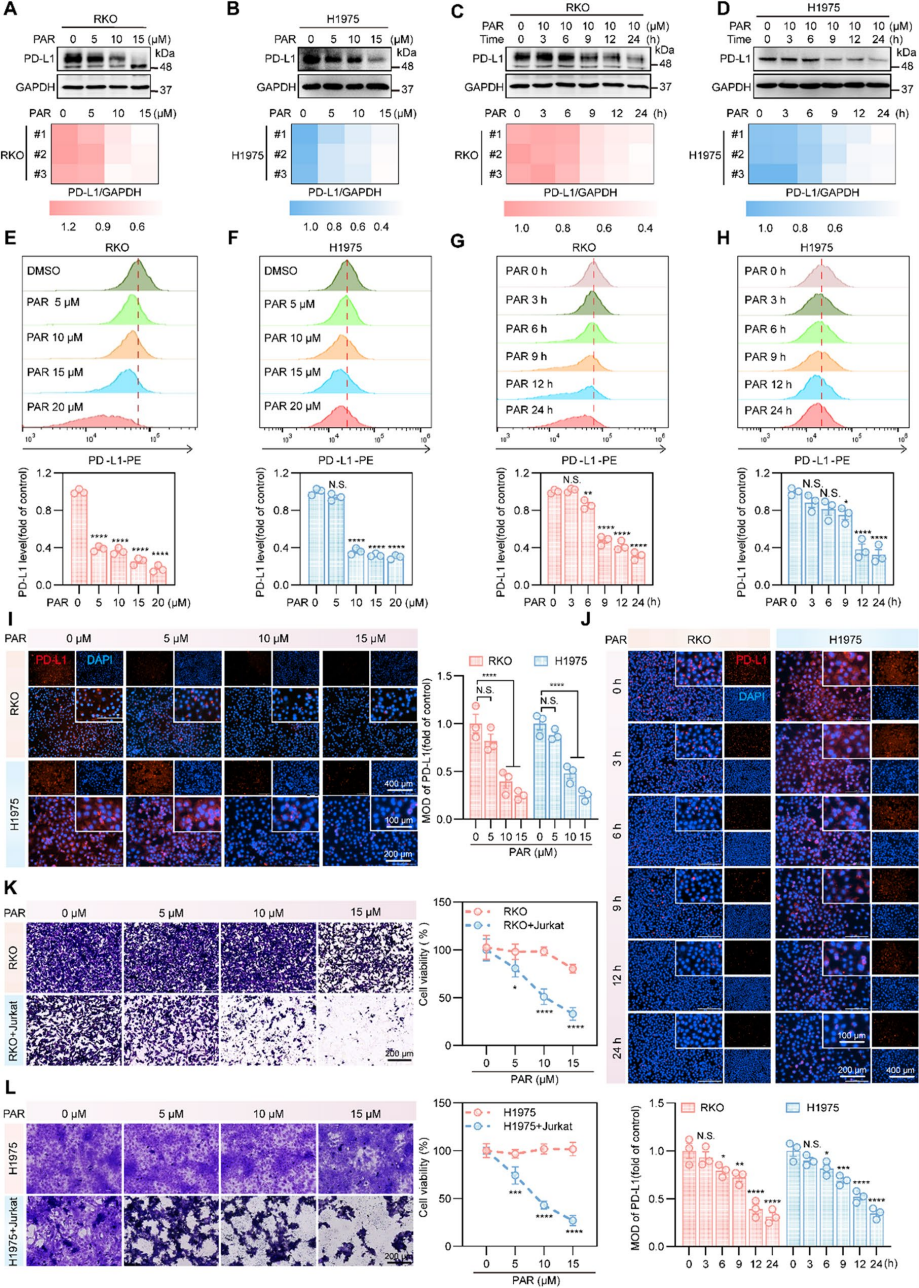
PAR reduces PD-L1 in tumor cells, increasing T cell killing in vitro. **A**-**D** Western blotting assay for PD-L1 protein expression after treatment of RKO (**A**) or H1975 (**B**) cells with different concentrations of PAR for 24 h and PD-L1 levels after treatment of RKO (**C**) or H1975 (**D**) cells with 10 µM PAR for different durations. **E**-**H** Flow cytometry analysis of PD-L1 levels on the membranes of RKO (**E**) or H1975 (**F**) cells treated with different concentrations of PAR for 24 h. The same method was also used to detect changes in PD-L1 on the membranes of RKO (**G**) and H1975 (**H**) cells after treatment with the same concentration of PAR (10 µM). **I** and **J** Expression of membrane PD-L1 detected by immunofluorescence after treating RKO and H1975 cells with different concentrations of PAR for 24 h (**I**) or treating RKO and H1975 cells with PAR (10 µM) for different periods of time (**J**). DAPI staining in blue indicates the cell nucleus, red indicates PD-L1, and the scale bar represents 200 μm. **K** and **L** Jurkat cells were cocultured with RKO (**K**) and H1975 (**L**) cells treated with various concentrations of PAR in a 12-well plate for 48 h, after which the number of surviving cells was assessed via crystal violet staining. Data are presented as mean ± SEM. Statistical significance was determined by one-way ANOVA with Dunnett’s test (*p < 0.05, **p < 0.01, ***p < 0.001, ****p < 0.0001; N.S., not significant)

We selected RKO colon cancer cells and H1975 lung cancer cells, both of which highly express PD-L1 [30], for PAR treatment and found that PAR downregulated PD-L1 protein levels in both cell lines in a concentration- and time-dependent manner (Fig. 1A-D). The effect of PAR on PD-L1 expression on tumor cell membranes was then detected via flow cytometry and immunofluorescence. As shown in Fig. 1E-J, the downregulation of PD-L1 expression on RKO and H1975 cell membranes was observed to be both time- and dose dependent. A similar downregulation effect was also observed in the MC38 cell line (Figure S1A-S1F). In addition, we examined the effects of different concentrations of PAR on the viability and proliferation of RKO, H1975 and MC38 cells, and the results revealed that PAR had no cytotoxic effects at working concentrations (Figure S2A-S2E). Moreover, we also examined the effects of PAR on PD-L1 expression on the membranes of additional colon cancer cells (DLD1, HT29, and HCT116), H460 lung cancer cells, AGS gastric cancer cells and the human metaplastic large cell lymphoma cell line Karpass-299 via flow cytometry (Figure S3A-S3H). The results demonstrate that PAR exerts a broad-spectrum downregulating effect on PD-L1. To investigate whether this effect is selective, we further examined the potential degradation of other immune checkpoint proteins, such as FGL1 and CD47, by PAR via Western blot, and compared the impact of fluoxetine—another serotonin reuptake inhibitor—on PD-L1 expression. The data showed in Figure S3I-S3K that PAR did not significantly alter the protein levels of FGL1 or CD47, and fluoxetine likewise failed to downregulate PD-L1 in RKO cells, indicating that the degradation of PD-L1 by PAR is specific.

Furthermore, in a co-culture assay using PD-1‑overexpressing Jurkat cells and cancer cells treated with varying concentrations of PAR, PAR significantly enhanced the cytotoxicity of Jurkat cells toward cancer cells **(**Fig. 1K and L**)**. Together, these findings suggest that PAR promotes T cell‑mediated antitumor activity in vitro through the specific downregulation of PD-L1.

### PAR inhibits cancer growth in vivo by activating T cell immunity

**Fig. 2:**
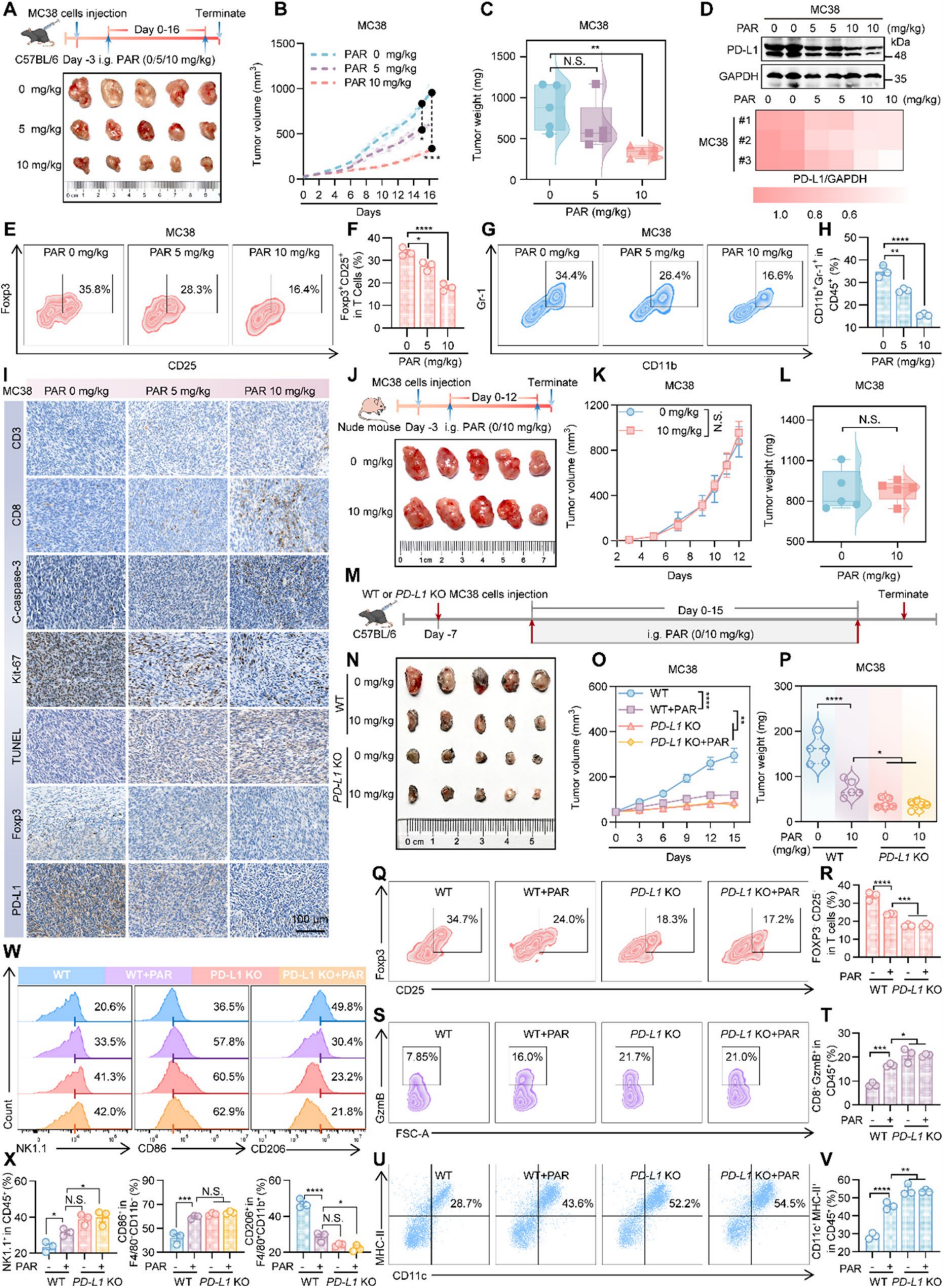
PAR activates immune suppression and inhibits colon cancer growth in vivo by degrading PD-L1.** A**-**I **Dose-Response Study in Immunocompetent Mice: MC38 colon cancer cells were subcutaneously inoculated into female C57BL/6J mice (*n* = 5 per group) and treated with various oral doses of PAR. Key results include: (**A**) Experimental schematic and representative tumor photographs; (**B**) Individual tumor growth curves; (**C**) Tumor weights; (**D**) PD-L1 expression in tumor tissues by Western blot and quantification; (**E**, **G**) Flow cytometry analysis of regulatory T cells (CD4^+^CD25^+^Foxp3^+^) and myeloid-derived suppressor cells (CD11b^+^Gr-1^+^), quantified in (**F**, **H**); (**I**) Immunohistochemical (IHC) staining of tumor tissues for CD3, CD8, cleaved caspase-3, Ki-67, TUNEL, Foxp3, and PD-L1 (scale bar: 100 μm). **J**-**L** Efficacy Validation in an Immunodeficient Model: A subcutaneous model was established in female nude mice by injecting MC38 cells into the armpit (*n* = 5 per group), followed by oral administration of 0 or 10 mg/kg PAR. Results show: (**J**) Experimental schematic and representative tumors; (**K**) Tumor growth curves; (**L**) Tumor weights. **M**-**X** PD-L1 Knockout Validation Experiment: C57BL/6 mice were subcutaneously injected with 1 million WT or PD-L1 knockout (KO) MC38 cells and divided into four groups (*n* = 5): WT, WT + PAR (10 mg/kg), PD-L1 KO, and PD-L1 KO + PAR. Analyses include: (**M**) Experimental schematic; (**N**) Representative tumors; (**O**) Tumor growth curves; (**P**) Tumor weights; (**Q**, **S**, **U**) Flow cytometry analysis of regulatory T cells, cytotoxic T cells (CD8^+^GzmB^+^), and dendritic cells (CD11c^+^MHC-II^+^), quantified in (**R**, **T**, **V**); (**W**) Flow cytometry analysis of NK cells (NK1.1^+^), M1 macrophages (CD86^+^), and M2 macrophages (CD206^+^), quantified in (**X**). Data are presented as mean ± SEM. Statistical significance was determined by two-way ANOVA with Dunnett’s test (*p < 0.05, **p < 0.01, ***p < 0.001, ****p < 0.0001; N.S., not significant)

Since PAR can enhance T cell-mediated tumor cytotoxicity in vitro, we next explored whether PAR has the same effect in vivo by constructing subcutaneous tumor models of colon cancer and lung cancer in C57BL/6J mice. As shown in Fig. 2A-C and Figure S5A-S5C, PAR inhibited tumor growth and tumor weight in a dose-dependent manner in mice inoculated with MC38 colon cancer cells, and the same results were also obtained in the lung cancer model (Figure S4A-S4F). Additionally, throughout the entire experimental process, the impact of PAR on the body weight of the mice was almost negligible (Figure S5D and Figure S4G). After PAR treatment, the expression of PD-L1 in mouse tumors decreased in a dose-dependent manner (Fig. 2D and Figure S4H). Then, to explore whether PAR activates the immune system in mouse tumors, flow cytometry was used to detect myeloid-derived suppressor cells (MDSCs) and regulatory T cells (Tregs), which promote the depletion of T cells in the tumor microenvironment of mice [31–33], and we also examined the level of granzyme B, which represents an indicator of cytotoxic T cell activation [34, 35], according to the assay method described in the relevant article [20, 24, 30]. The results revealed that the number of activated MDSCs (CD11b^+^Gr-1^+^) and Tregs (CD4^+^CD25^+^Foxp3^+^) decreased significantly with increasing PAR dosage (Fig. 2E-H and Figure S4I-S4L), whereas the level of granzyme B was positively correlated with PAR dosage (Figure S5E-S5F and Figure S4M-S4N). Moreover, the immunohistochemistry (IHC) results showed that the levels of Ki-67, Foxp3 and PD-L1 in mouse tumor tissues decreased with increasing doses of PAR, whereas the levels of CD3, CD8, TUNEL and cleaved caspase-3 increased after PAR treatment (Fig. 2I, Figure S4O and Figure S5H-S5I), suggesting that PAR inhibits the proliferation and promotes the apoptosis of tumor cells. In addition, relevant studies have shown that PAR can produce anticolorectal cancer effects by promoting the apoptosis of cancer cells [16]. However, the inhibitory effects of PAR on MC38 and Lewis tumors in T cell deficient nude mice are not obvious (Fig. 2J-L and Figure S4P-S4R), which may be attributed to the differences in the tumor microenvironments resulting from the inoculation of different cancer cells and suggests that the antitumor effects exerted by PAR are mainly through the activation of T cell immunity. Moreover, consistent with the previous results, PAR administration had no significant effect on the body weight of the nude mice (Figure S5G and Figure S4S), and H&E staining of the internal organs of the C57BL/6 mice or the nude mice in the subcutaneous tumor model of colon and lung cancers revealed that PAR did not cause any damage to the internal organs of the mice (Figure S6A-S6B). These results indicate that PAR can exert antitumor effects in vivo by activating the immune system.

To investigate whether paroxetine exerts its antitumor effect by degrading tumor cell PD-L1 and relieving immune suppression, we generated a PD-L1-knockout (KO) MC38 cell line. After confirming knockout efficiency by flow cytometry in Figure S5J, these cells were subcutaneously inoculated into C57BL/6 mice. The experimental design included groups implanted with either wild-type (WT) or KO tumor cells, each of which was treated with either PAR or vehicle control. Therapeutic outcomes are presented in Fig. 2M-P. No significant difference in tumor volume was observed between the PD-L1 knockout group and the PD-L1 knockout group treated with paroxetine. Tumor volumes in both groups were significantly smaller than those in the wild-type control group and were also modestly reduced compared with the wild-type group receiving PAR. Throughout the treatment period, body weight did not differ significantly among the groups, suggesting that the drug regimen was well tolerated (Figure S5K). Collectively, these results indicate that paroxetine does not further suppress tumor growth following PD-L1 knockout, supporting the conclusion that its antitumor activity depends on the presence of PD-L1 in tumor cells. To further characterize alterations in the tumor immune microenvironment, we analyzed immune cell subsets and associated functional markers in tumor tissues by flow cytometry. The Fig. 2Q-X and Figure S5L-S5M showed that compared with the WT group, PD-L1 knockout tumors displayed a significant increase in immune effector cells and activation markers, including CD3⁺ T cells, CD8⁺ T cells, CD8⁺GZMB⁺ cytotoxic T cells, CD11c⁺MHC-II⁺ dendritic cells, NK1.1⁺ NK cells, and CD86⁺ M1-type macrophages. Conversely, immunosuppressive cell populations were notably reduced, with both CD4CD25⁺Foxp3⁺ regulatory T cells and CD206⁺ M2-type macrophages showing significant decreases. It is noteworthy that PD-L1 expression on dendritic cells (CD11c⁺MHC-II⁺PD-L1^+^) did not differ significantly between the WT group and the WT group treated with PAR. However, this marker was slightly lower in both the PD-L1 KO group and the PD-L1 KO group treated with PAR relative to the WT group, suggesting that deletion of tumor cell PD-L1 may indirectly modulate PD-L1 expression on dendritic cells within the tumor microenvironment (Figure S5N).

In summary, PD-L1 knockout alone was sufficient to inhibit tumor growth, and PAR provided no additional therapeutic benefit under these conditions. The immune microenvironment in KO tumors exhibited a broadly activated antitumor state. These findings further support the mechanism whereby paroxetine exerts its antitumor effects by targeting tumor cell PD-L1, thereby alleviating immune suppression and promoting antitumor immunity.

### PAR promotes ubiquitinated degradation of PD-L1

**Fig. 3:**
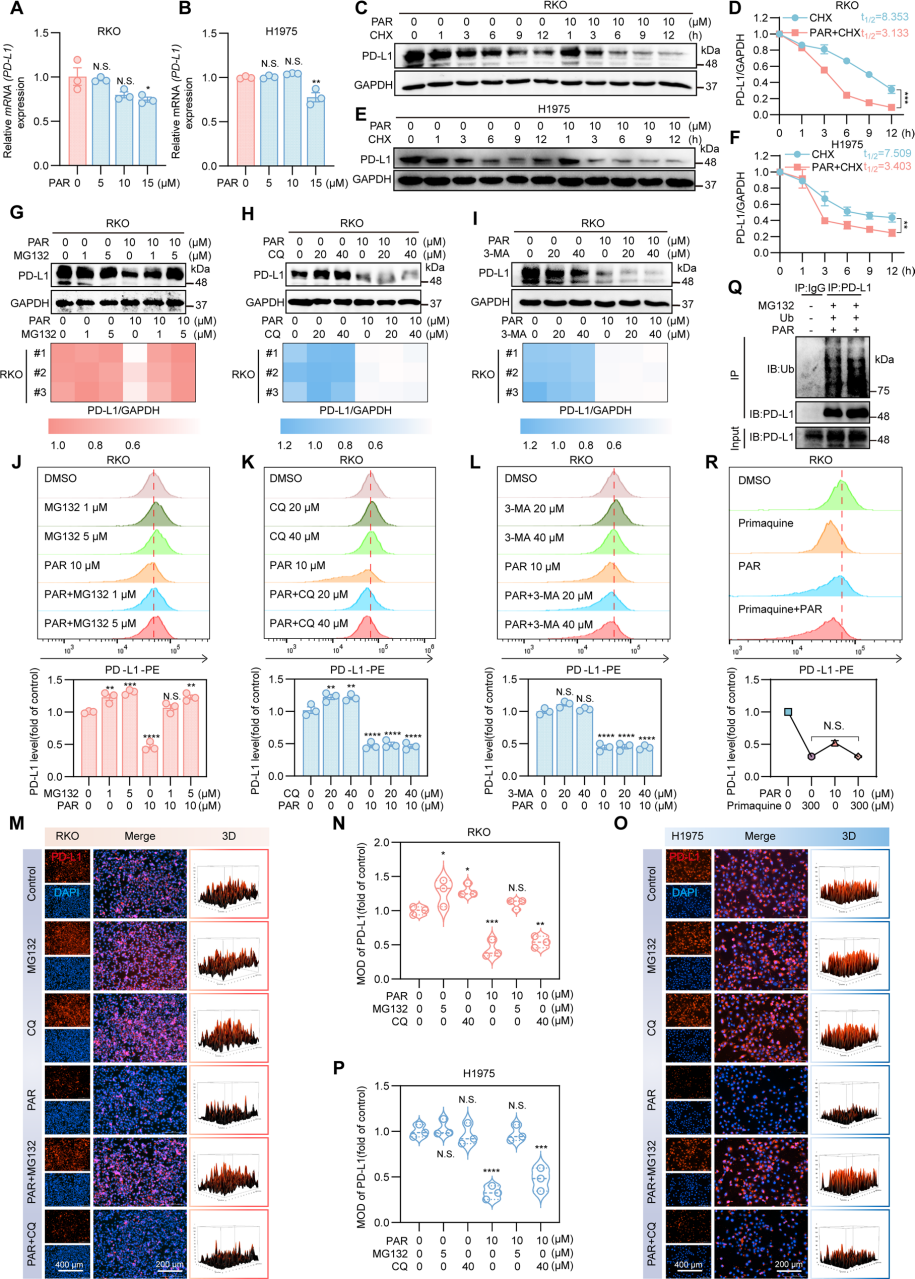
PAR downregulates PD-L1 via the ubiquitin‒proteasome pathway. **A** and **B** RT-PCR analysis of PD-L1 levels after treatment of RKO (**A**) and H1975 (**B**) cells with different concentrations of PAR. **C**-**F** Western blotting was performed to detect the expression of PD-L1 after treatment of RKO (**C**) and H1975 (**E**) cells with PAR (10 µM) for different durations in the presence of CHX (50 mg/mL). (**D)** and (**F**) Quantification curves of (**C**) and (**E**), respectively. **G-I** Western blotting was conducted to measure PD-L1 protein levels in RKO cells treated with PAR and either the proteasome inhibitor MG132 (**G**), the lysosomal inhibitor chloroquine (**H**), or the autophagy inhibitor 3-methyladenine (**I**), followed by statistical quantification. **J-L** Flow cytometry analysis of PD-L1 expression on RKO cell membranes after cotreatment with PAR and MG132 (**J**), CQ (**K**), or 3-MA (**L**). **M-P** RKO (**M**) or H1975 (**O**) cells were treated for 12 h with 10 µM PAR combined with either 5 µM MG132 or 40 µM chloroquine. DAPI staining in blue revealed cell nucleus, while red fluorescence indicated PD-L1 on cell membranes. The scale bar represents 200 μm. (**N**) and (**P**) Quantification of (**M**) and (**O**), respectively. (**Q**) Ub overexpression in RKO cells was followed by the detection of PAR-induced PD-L1 ubiquitination through immunoprecipitation with Flag beads and immunoblotting with an anti-Ub antibody. **R** Flow cytometry was used to analyze PD-L1 levels on cell membranes after treatment of RKO cells with PAR in combination with the endocytosis inhibitor primaquine. Data are presented as mean ± SEM. Statistical significance was determined by one-way ANOVA with Dunnett’s test (*p < 0.05, **p < 0.01, ***p < 0.001, ****p < 0.0001; N.S., not significant)

To explore the pathway through which PAR reduces the expression of PD-L1, we first examined the effect of PAR on PD-L1 RNA expression. As shown in Fig. 3A and B, PAR had little effect on the RNA expression levels of PD-L1 in RKO and H1975 cells. To confirm that the degradation of PD-L1 by PAR is a result of posttranslational regulation, we exposed RKO cells to the protein translation inhibitor cycloheximide (CHX). In the presence of CHX, the turnover rate of PD-L1 in cells treated with PAR was faster than that in untreated cells (Fig. 3C-F), indicating that the downregulation of PD-L1 triggered by PAR is primarily controlled at the protein level. The degradation of PD-L1 protein involves mainly the ubiquitin-proteasome pathway and the lysosome-dependent degradation pathway [36–39]. Therefore, to determine which pathway PAR specifically downregulates PD-L1 protein, we treated RKO cells with the proteasome inhibitor MG132, the lysosome inhibitor chloroquine (CQ), and 3-methyladenine (3-MA). After 12 h, we detected PD-L1 expression via Western blotting and flow cytometry, and the results revealed that only MG132 reversed the degradation of PD-L1 by PAR (Fig. 3G-L). We further verified these results via Western blotting (Figure S7A-S7D) and immunofluorescence experiments (Fig. 3M-P). Immunoprecipitation experiments also showed that the level of ubiquitination of PD-L1 was significantly increased after PAR treatment (Fig. 3Q). These results collectively indicate that PAR promotes the ubiquitination and subsequent degradation of the PD-L1 protein. Since intracellularly synthesized and modified PD-L1 may not always function on the cell membrane surface or be secreted from the cell but may also be transferred from the cell membrane surface to the cytoplasm, it affects the immune escape of tumor cells [21, 38, 40–42]. To determine whether PAR influences the process of PD-L1 protein turnover, we used the intracellular recycling inhibitor primaquine to treat RKO cells as previously described [21]. Flow cytometry analysis revealed that the PD-L1 protein on the cell surface rapidly disappeared following incubation with primaquine, indicating an inability to be recycled back to the plasma membrane. However, PAR did not further promote or accelerate the degradation of PD-L1 induced by primaquine (Fig. 3R). These findings suggest that PAR may inhibit the expression of the PD-L1 protein by blocking the recycling pathway of internalization.

### PAR promotes T cell killing by targeting SPOP to downregulate PD-L1

**Fig. 4:**
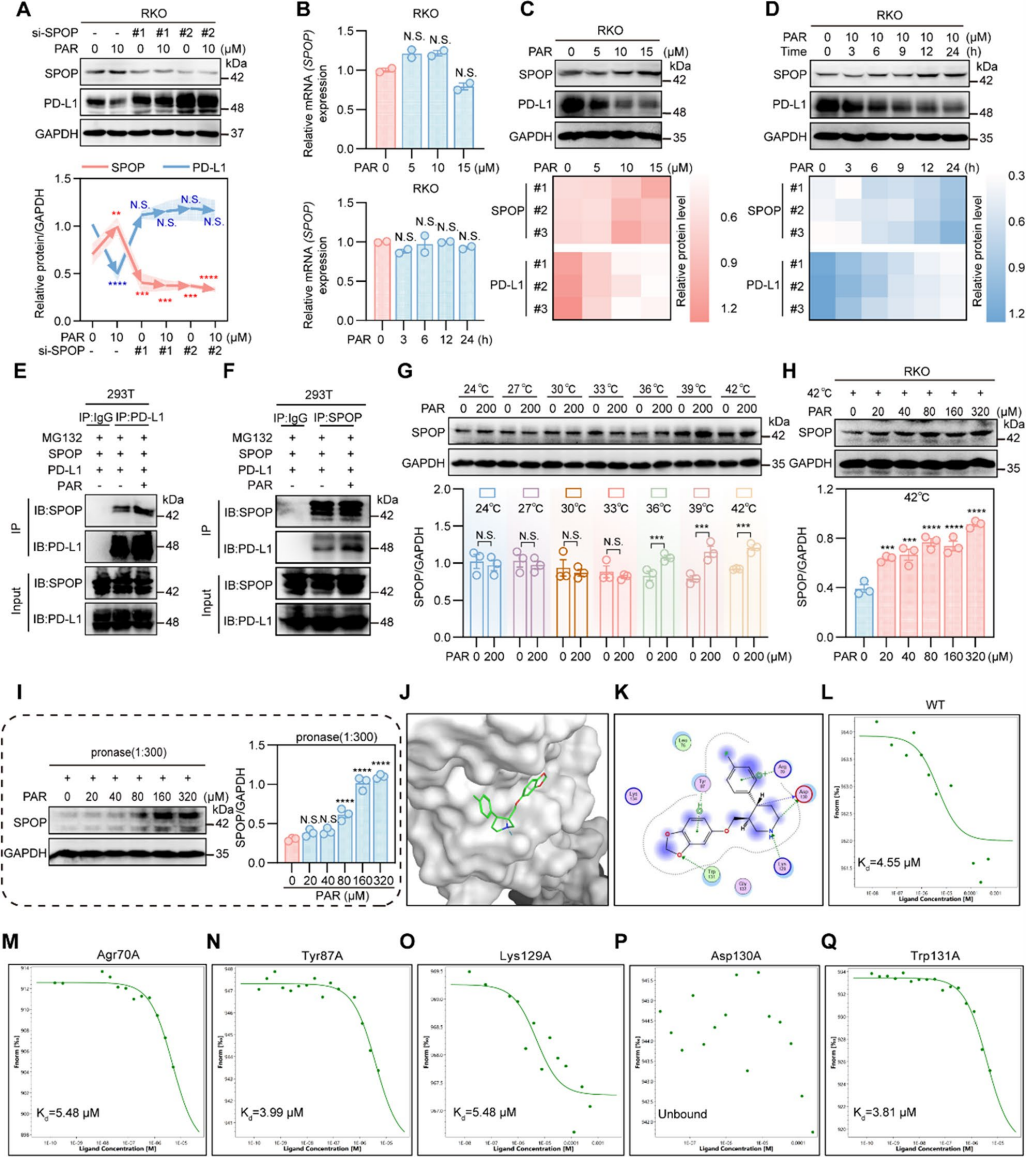
PAR targeting SPOP enhances PD-L1 degradation. **A** Protein expression of SPOP and PD-L1 in PAR-treated RKO cells was evaluated via Western blotting after treatment with siRNA targeting SPOP or a negative control (siRNA-NC). **B** RT-qPCR was conducted to measure PD-L1 RNA levels in RKO cells treated with various PAR concentrations for 24 h or with a fixed PAR concentration over different time periods. **C** and **D** The total protein profile of SPOP was examined via Western blotting after treating RKO cells with different PAR concentrations for 24 h (**C**) or with 10 µM PAR for various durations (**D**). **E** and **F** 293T cells were treated with or without PAR (10 µM) for 12 h and MG132 (10 µM) for 6 h. Exogenous SPOP (**E**) or PD-L1 (**F**) was pulled down with PD-L1 antibody (**E**) or SPOP antibody (**F**), respectively, and then PD-L1 and SPOP were detected by western blotting. **G** The thermal stability of SPOP was assessed via the CETSA method at temperatures of 24 °C, 27 °C, 30 °C, 33 °C, 36 °C, 39 °C, and 42 °C when interacting with PAR. **H** and **I** The effects of different PAR concentrations on SPOP protein stability at 42 °C were analyzed (**H**), as was the stability of SPOP after treatment with varying PAR concentrations at a 1:300 pronase-to-protein ratio (**I**). **J** and **K** Molecular docking of PAR with SPOP is illustrated (**J**), along with potential binding sites (**K**). **L**-**Q** The binding of PAR to wild-type SPOP (**L**) and mutant forms at Agr70A (**M**), Tyr87A (**N**), Lys129A (**O**), Trp131A (**P**), and Asp130A (**Q**) was detected via microcalorimetric thermophoresis. Data are presented as mean ± SEM. Statistical significance was determined by two-way ANOVA with Dunnett’s test (*p < 0.05, **p < 0.01, ***p < 0.001, ****p < 0.0001; N.S., not significant)

In proteasomal degradation, ubiquitination involves E1-activating enzymes, E2-binding enzymes, and E3 ligases that recognize target proteins. Deubiquitinating enzymes are influenced by phosphorylation, ubiquitination, and SUMOylation, which regulate substrate protein deubiquitination, protein function, and various cellular activities. As shown in Figure S8A-S8E and Fig. 4A, we transfected RKO cells with siRNAs targeting various E3 ligases (MARCH8 [43, 44], BTRC [45–47], HRD1 [20, 48–51], ARIH1 [52], STUB1 [40], and SPOP [21, 22, 53, 54]) and detected the knockdown efficiency via RT-qPCR. The aforementioned RKO cells were subsequently treated with PAR for 24 h, after which the protein expression of PD-L1 was detected via Western blotting. Interestingly, only the knockdown of SPOP reversed the downregulation of PD-L1 by PAR. Similarly, the overexpression of deubiquitinating enzymes such as USP22 and OTUB1 did not reverse the degradation of PD-L1 by PAR (Figure S8F). Therefore, we hypothesize that SPOP is a potential target for PAR in the degradation of PD-L1. To test this hypothesis, we examined whether PAR affects the transcription or protein levels of SPOP via RT-qPCR. The results revealed that PAR had no effect on the transcriptional level of SPOP (Fig. 4B), but there was an increase in the SPOP protein **(**Fig. 4C and D). This finding implies that PAR may enhance tumor suppression by upregulating SPOP proteins, which are integral to cell cycle regulation and tumor suppression [53, 55].

To validate this hypothesis, we used immunoprecipitation experiments, which revealed that PAR enhanced the interaction between SPOP and PD-L1 **(**Fig. 4E and F). We employed a cellular thermal shift assay (CETSA) to determine whether PAR directly interacts with SPOP [30, 56, 57]. As shown in Fig. 4G, PAR (200 µM) at 36 °C, 39 °C, and 42 °C could enhance the thermal stability of the SPOP protein. Furthermore, we also observed that at a fixed temperature of 42 °C, the stability of the SPOP protein increased with increasing PAR concentration (Fig. 4H). Moreover, PAR resists the degradation of the SPOP protein by proteases in a concentration-dependent manner (Fig. 4I). These results indicate a binding interaction between SPOP and PAR. Next, we simulated potential binding modes and sites for PAR and SPOP via Molecular Manipulation Environment (MOE) software (Fig. 4J and K). We transfected 293T cells with GFP-tagged wild-type SPOP and the Agr70A, Try87A, Lys129A, Asp130A, and Trp131A mutant plasmids. After the cell lysates were collected, microscale thermophoresis (MST) was used to detect PAR binding to SPOP. The results revealed that only the Asp130A mutation influenced PAR binding to SPOP (Fig. 4L-Q), confirming that Asp130 is the binding site for SPOP and PAR.

**Fig. 5:**
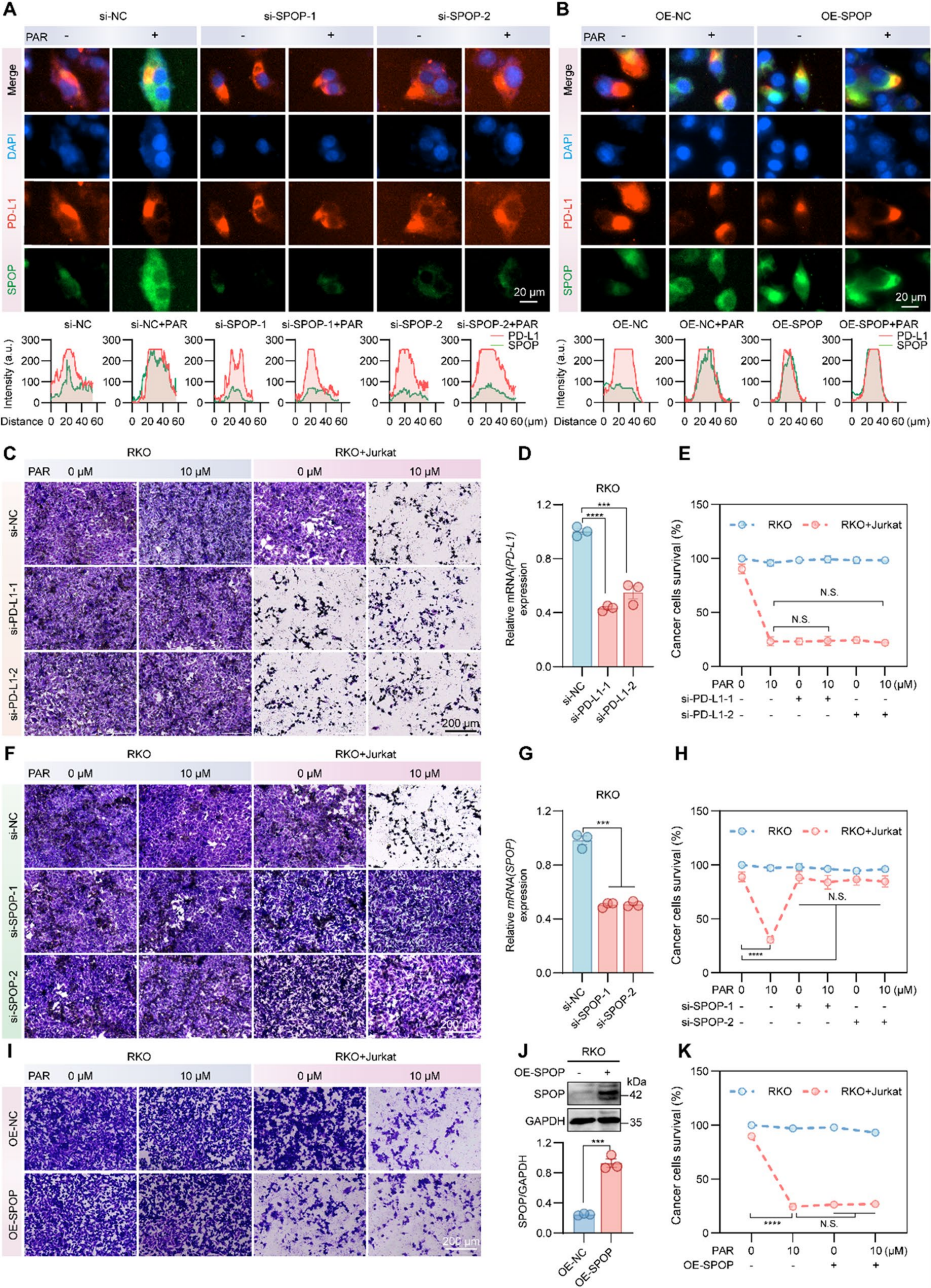
PAR enhances T cell killing in vitro by targeting SPOP to promote PD-L1 degradation. **A** and **B** RKO cells were treated with SPOP siRNA (**A**) or SPOP plasmid (**B**), followed by 12 h of PAR (10 µM) treatment. The interaction between PD-L1 and SPOP was then assessed using immunofluorescence. **C**-**H** Tumor cells that survived after being cocultured with PAR and Jurkat cells for a period of time after being subjected to siRNA for PD-L1 (**C**) or SPOP (**F**) were observed via crystal violet staining. (**D)** and (**G**) Knockdown efficiencies of PD-L1 and SPOP assessed by RT-qPCR, respectively. (**E)** and (**H**) Statistical quantitative plots of surviving tumor cells in (**C**) and (**F**), respectively. **I**-**K** Tumor cells were subjected to crystal violet staining of RKO-overexpressing SPOP (**I**) treated with PAR for 12 h and then were cocultured with Jurkat cells for a period of time. (**J)** Overexpression efficiency of SPOP in RKO cells detected via Western blotting. (**K)** Statistical analysis of the number of surviving tumor cells in (**I**). Data are presented as mean ± SEM. Statistical significance was determined by one-way ANOVA with Dunnett’s test (*p < 0.05, **p < 0.01, ***p < 0.001, ****p < 0.0001; N.S., not significant)

Previous in vitro experiments have shown that PAR enhances the killing of tumor cells by T cells (Fig. 1K and L) and immunofluorescence showed reduced co-localization of SPOP and PD-L1 in RKO cells following SPOP knockdown, and the overexpression of SPOP did not further increase PAR-promoted co-localization (Fig. 5A and B). We explored whether this effect was generated through PAR targeting SPOP to promote PD-L1 degradation. PD-L1 and SPOP were first knocked down in RKO cells, which were then cotreated with PAR and Jurkat cells overexpressing PD-1. The results revealed that the effects of PD-L1 knockdown on tumor cell killing by T cells were similar to those of PAR alone, and the combination of the two did not significantly enhance T cell activity (Fig. 5C-E). However, the knockdown of SPOP reversed the promotional effect of PAR on the killing of tumor cells by T cells (Fig. 5F-H), and overexpression of SPOP did not further enhance PAR-promoted T cell killing (Fig. 5I-K), suggesting that PAR achieves its antitumor effect by targeting SPOP.

### PAR prevents colon carcinogenesis induced by AOM/DSS in vivo

**Fig. 6:**
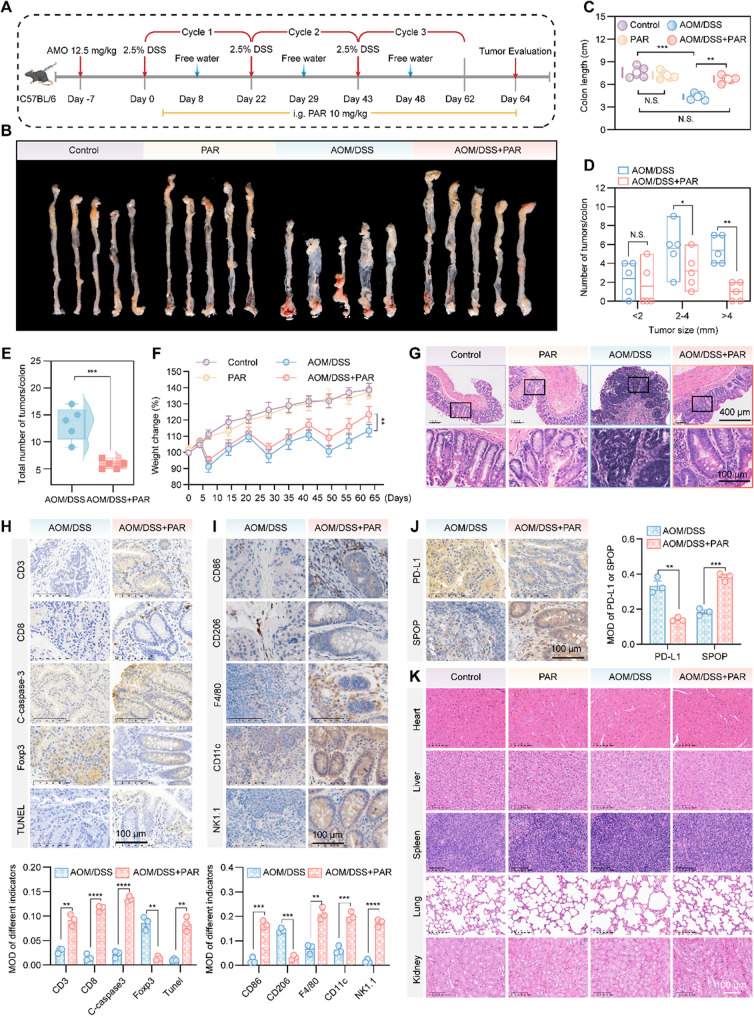
PAR suppresses colon cancer induced by AOM/DSS. **A** Schematic diagram of the CRC induction model. Four groups of 10 male C57BL/6J mice each were established: a blank group, a PAR group, an AOM/DSS model group, and an AOM/DSS + PAR group. The AOM/DSS model and AOM/DSS + PAR groups were injected with azomethane (AOM) and then treated with dextran sodium sulfate (DSS) for three cycles after one week, with PAR administered from the start of DSS treatment until the study’s conclusion. **B** Representative images of the colons and rectums of each group. **C** Measurement of total colon length at the end of the study, indicating inflammation severity. **D** and **E** Number and diameter of tumors in the colon and rectum of the AOM/DSS-treated groups. **F** Changes in body weight from day 0 to day 65, normalized to 100 on day 0. **G** Representative H&E staining micrographs of the colon in different groups. **H**-**J** Immunohistochemical detection of CD3, CD8, C-caspase3, Foxp3, and TUNEL content in colon tissues from the AOM/DSS and AOM/DSS + PAR groups (**H**), along with expression levels of CD86, CD206, F4/80, CD11c, and NK1.1 (**I**), as well as SPOP and PD-L1 content, which were analyzed similarly (**J**); scale bar = 100 μm. **K** H&E staining of viscera from different representative groups of mice; scale bar = 100 μm. Data are presented as mean ± SEM. Statistical significance was determined by ANOVA test (*p < 0.05, **p < 0.01, ***p < 0.001, ****p < 0.0001; N.S., not significant)

To verify the anticancer effect of PAR, we used azoxymethane (AOM) [58] and dextran sodium sulfate (DSS) [25, 59] to simulate the process of ulcerative colitis-associated colorectal cancer in C57BL/6J mice and to gain a deeper understanding of the mechanism of PAR in antitumor immunity, as well as its potential synergistic effects when used in combination with other immunotherapies (Fig. 6A). The mice were divided into four groups: the normal control group, the PAR treatment group, the AOM/DSS treatment group, and the AOM/DSS plus PAR combined treatment group. At the end of the experiment, analysis of the colon and rectum revealed that compared with the model group, the AOM/DSS plus PAR group had fewer and smaller tumors (Fig. 6B). Additionally, the length of the colon indicated that PAR mitigated the inflammatory response induced by DSS (Fig. 6C). Compared with those in the model group, there was a 41.5% reduction in the number of large tumors (greater than 4 mm), a 42.8% reduction in the number of medium-sized tumors (2–4 mm), and a 33.3% reduction in the number of small tumors (less than 2 mm) in the PAR treatment group (Fig. 6D), with an overall 56.7% decrease in the total number of tumors (Fig. 6E). Weight change assessment revealed that, compared with that in the AOM/DSS group, the decrease in mouse body weight significantly slowed after PAR treatment (Fig. 6F). In addition, HE staining showed that the progression from adenoma to carcinoma in the PAR treatment group was markedly improved (Fig. 6G**)**, suggesting that PAR ameliorates the occurrence and development of colorectal cancer induced by AOM/DSS. Next, we conducted immunohistochemical analysis of colon tissues from two groups of mice treated with AOM/DSS. The results revealed a significant increase in CD3, CD8, C-caspase3, and TUNEL, along with a notable decrease in Foxp3 in the model group after PAR administration (Fig. 6H). Additionally, the increase in CD86 and F4/80 and the decrease in CD206 indicate that the proportion of M1-type tumor-associated macrophages is higher than that of M2-type macrophages (Fig. 6I), suggesting that PAR not only inhibits the development of colorectal cancer induced by AOM/DSS but also improves the tumor immune microenvironment. Furthermore, the increased levels of NK1.1 and CD11c in the tissues after PAR treatment further supported these findings (Fig. 6I). As shown in Fig. 6J, the level of PD-L1 in the colon of the mice in the PAR treatment group was significantly lower than that in the AOM/DSS group, whereas the level of SPOP was significantly higher, further indicating that PAR exerts its antitumor effect by targeting SPOP to promote the degradation of PD-L1. Finally, H&E staining of organs from the mice in the different groups confirmed that PAR had little effect on the mice (Fig. 6K). In summary, PAR has a significant inhibitory effect on the development of AOM/DSS-induced colon cancer.

### PAR has anticancer effects similar to those of anti-PD-1 and boosts efficacy with anti-CTLA4

**Fig. 7:**
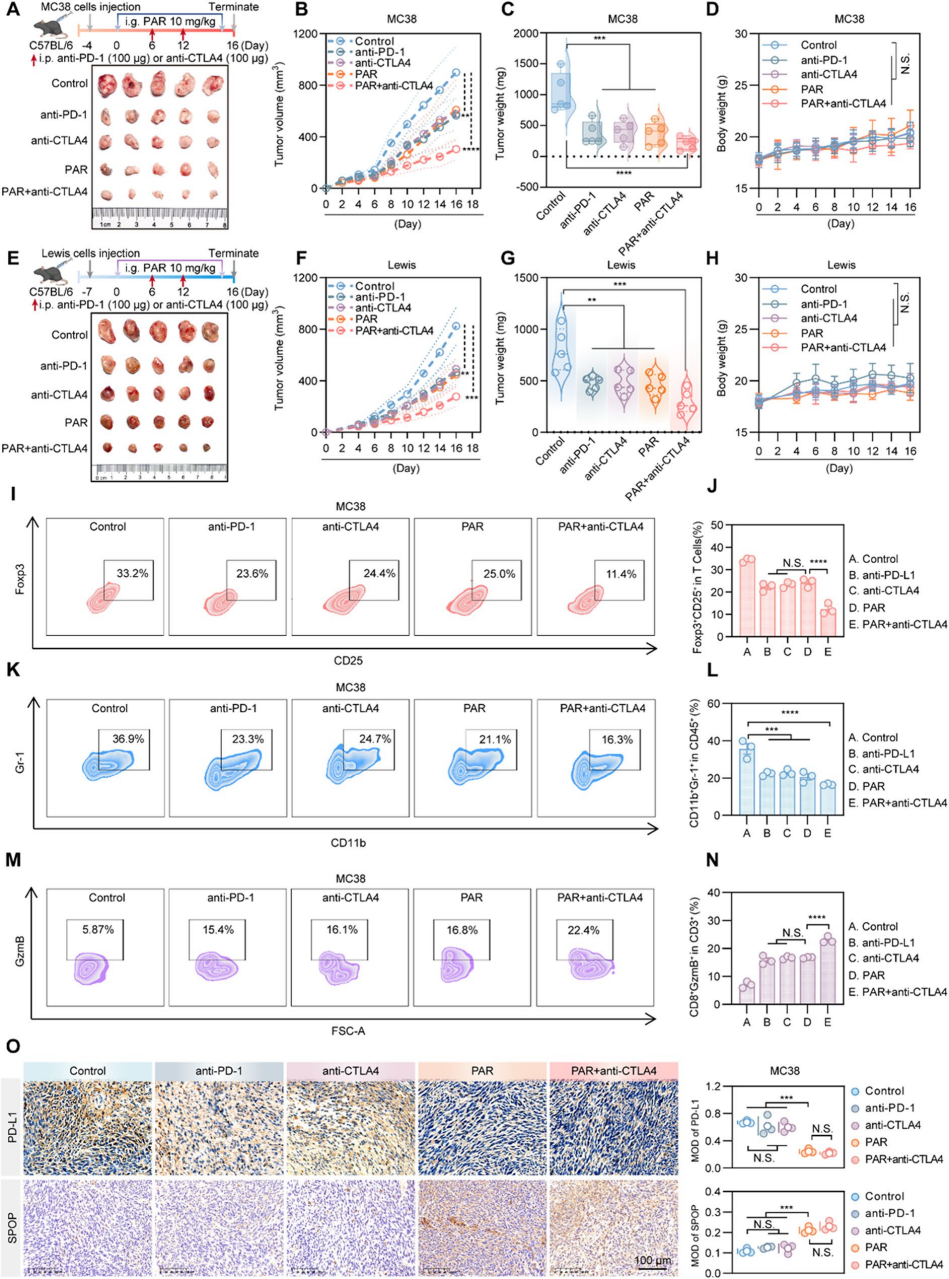
Combination therapy using PAR and anti-CTLA4 enhances the efficacy in colorectal cancer. **A**-**H** Colon cancer cells (MC38, 8 × 10^6^ cells/mouse) or lung cancer cells (Lewis, 3 × 10^7^ cells/mouse) or were injected subcutaneously into female C57BL/6J mice, which were divided into five groups, which were treated with corn oil, anti-PD-1, anti-CTLA4, PAR (10 mg/kg) or PAR combined with anti-CTLA4. A flow chart of the MC38 (**A**) or Lewis (**E**) transplant tumor treatment process (i.g., intragastric injection; i.p., peritoneal injection) and a schematic diagram of representative solid tumors in different groups of mice. (**B)** and (**F)** were the tumor growth curves of the mice that received different treatments. (**C)** and (**G)** were the weights of resected tumors from different groups of mice that received different treatments. (**D)** and (**H**) were the changes in the body weights of the mice in each group. **I-N** Flow cytometry analysis of CD4^+^CD25^+^Foxp3^+^ (**I**) and CD11b^+^Gr-1^+^ (**K**) as well as CD8^+^GzmB^+^ (**M**) levels in MC38 subcutaneous tumor tissues of mice treated via different methods. (**J**), (**L**) and (**N**) Quantitative results of (**I**), (**K**) and (**M**), respectively. **O** Immunohistochemistry showing the levels of PD-L1 and SPOP in the colon tumor tissues of different groups of mice; scale bar = 100 μm. The data shown are the mean value ± standard error of the mean (SEM). Data are presented as mean ± SEM. Statistical significance was determined by two-way ANOVA with Dunnett’s test (*p < 0.05, **p < 0.01, ***p < 0.001, ****p < 0.0001; N.S., not significant)

The CTLA4 antibody can not only reactivate the differentiation and proliferation of T cells to become effector T cells through blockade of CTLA4 but also clear the local Tregs of tumors with high expression of CTLA4 and release the immunosuppression of Tregs, thus exerting antitumor immunity [60]. We aimed to explore whether the antitumor effect of PAR is superior to that of anti-CTLA4 and anti-PD-1 alone in the treatment of subcutaneous tumors in C57BL/6J mice. Since PAR can activate effector T cells by reducing the expression of PD-L1, it theoretically complements the antitumor mechanism of anti-CTLA4. Therefore, we designed an additional treatment regimen involving the combination of PAR with anti-CTLA4. As shown in Fig. 7A-H and S9A-S9E, PAR, anti-PD-1, and anti-CTLA4 exhibited similar antitumor effects, while the combination of PAR with anti-CTLA4 further suppressed the growth of colon and lung cancer tumors, which was also reflected in the tumor growth curves and the statistical graphs of the tumor weights. In addition, the body weight graphs (Fig. 7D and H) and H&E staining (Figure S10A and S10B) of the viscera indicated no significant toxic effects from the administration groups on the mice.

Next, we examined the levels of activated MDSCs (CD11b^+^Gr-1^+^), Treg cells (CD4^+^CD25^+^Foxp3^+^), and granzyme B in tumor tissues from different treatment groups via flow cytometry. As shown in Fig. 7I-N and Figure S9F-S9K, the combined use of PAR and anti-CTLA4 antibodies significantly increased the number of activated MDSCsd (CD11bGr-1^+^) and reduced the number of Treg cells (CD4^+^CD25^+^Foxp3^+^); however, the granzyme B levels rose significantly, which indicates that the combined therapy has, to some extent, reversed immune suppression and enhanced the cytotoxicity of T cells to tumor cells.

To investigate changes in immune cells within the tumor microenvironment after various treatments, we analyzed T cells, NK cells, macrophages, and dendritic cells (DCs) via immunohistochemistry. As shown in Figures S11 and Figure S12, treatment with PAR significantly increased the numbers of CD3^+^, CD8^+^, and C-caspase-positive cells, as well as TUNEL-positive cells, while it decreased the proportions of Ki-67^+^ and Foxp3^+^ cells. Additionally, we observed a significant increase in tumor-infiltrating NK (NK1.1^+^) cells and M1-type macrophages (CD86^+^, F4/80^+^) and a decrease in immune-suppressing M2-type macrophages (CD206^+^). Furthermore, the proportion of antigen-presenting DCs (CD11c^+^) in the PAR plus anti-CTLA4 group significantly exceeded that in the other treatment groups. Immunohistochemical analyses also revealed negative regulation of PD-L1 and positive regulation of SPOP by PAR in tumor tissues (Figs. 7O and S9L). These findings suggest that PAR can serve as an alternative to anti-PD-1 or anti-CTLA4, when combined with anti-CTLA4, resulting in synergistic anticancer effects and suggesting a new treatment option for colon and lung cancers.

### SPOP levels are related to PD-1 mAb effectiveness in colon cancer and NSCLC

**Fig. 8:**
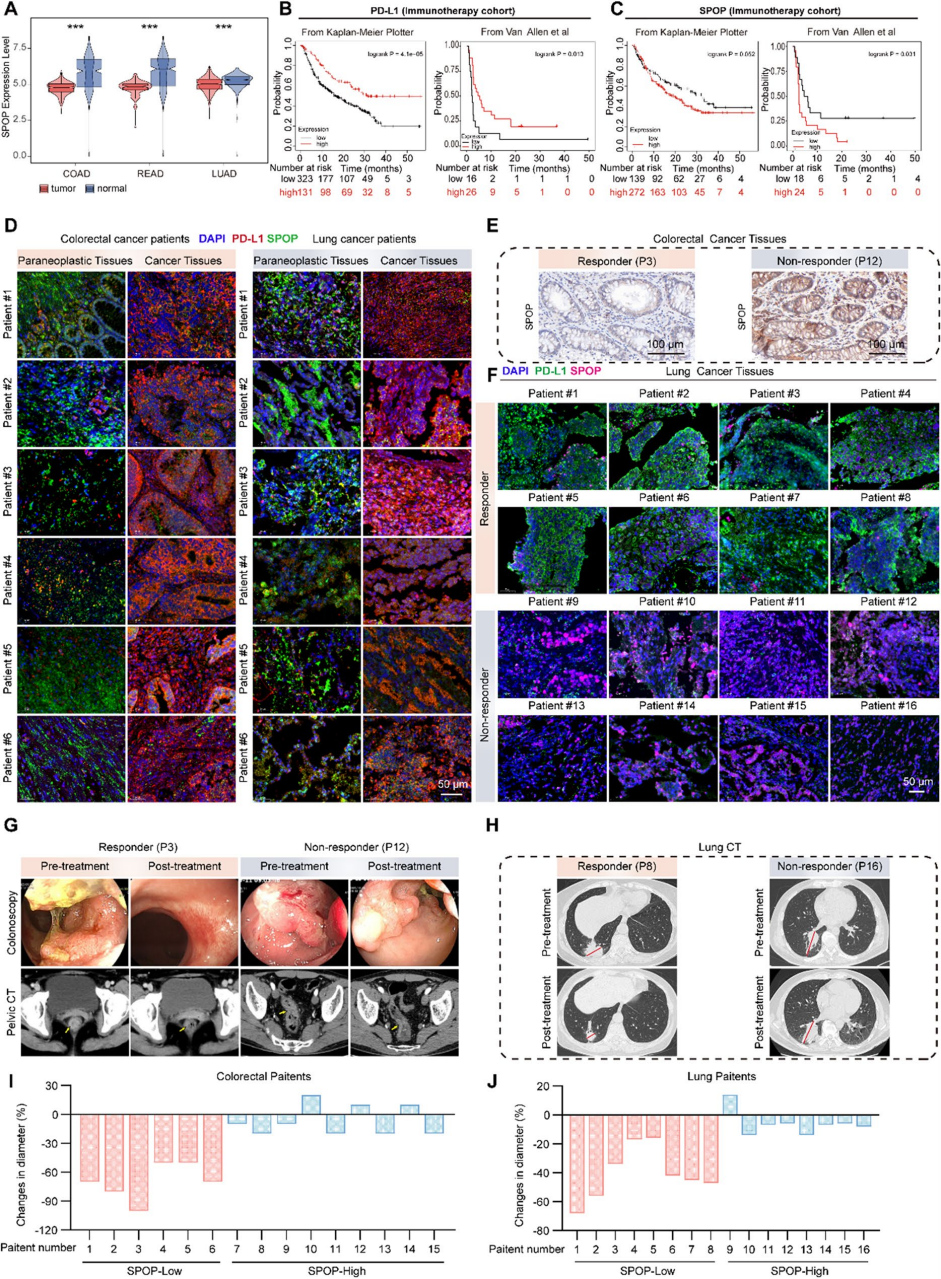
Clinical significance of SPOP in immunotherapy for colon and lung cancer patients. **A** Analysis of SPOP expression in cancer versus normal tissues, incorporating data from the TCGA and GTEx databases. **B-C** The survival of cancer patients stratified by the expression of PD-L1 or SPOP was compared by two-sided log rank analysis in immunotherapy cohorts. **D** Detection of PD-L1 and SPOP expression levels in paraneoplastic (PT) and carcinomatous (CT) tissues from colorectal and lung cancer patients via fluorescent double-stained immunohistochemistry (IHC). **E** Representative images of immunohistochemical staining for SPOP expression in rectal cancer tissues. Scale bars, 100 μm. **F** Representative images of IF staining for SPOP and PD-L1 expression in lung tissue. Scale bar, 50 μm. **G** and **H** Tumor image maps of two colon (**G**) and lung (**H**) cancer patients receiving PD-1 mAb therapy were analyzed before and after treatment, with arrows indicating the tumors. **I** and **J** Differences in tumor diameter between colon (**I**) and lung (**J**) cancer patients, respectively

The expression pattern of SPOP was explored, and analysis of the TCGA and GTEx databases revealed significantly lower expression levels of SPOP in COAD, READ, and LUAD tissues than in corresponding normal tissues **(**Fig. 8A**)**. Moreover, in immunotherapy-treated patients, higher PD-L1 levels were associated with better outcomes, whereas higher SPOP levels were linked to poorer outcomes. Specifically, patients with elevated PD-L1 and reduced SPOP expression experienced the greatest improvement in overall survival (Kaplan-Meier Plotter anti-PD-1 dataset; Van Allen et al. dataset; log-rank test *P* < 0.05 was considered significant), as depicted in Fig. 8B and C. We also analyzed PD-L1 and SPOP expression levels in paracancerous and cancerous tissues from colon and lung cancer patients. As shown in Figs. 8D and Figure S13A-S13B, PD-L1 expression was significantly higher in cancer tissues than in paracancerous tissues, whereas SPOP expression exhibited the opposite trend. These results indicate that elevated SPOP expression in tumors may inhibit PD-L1, enhancing anticancer efficacy.

We further assessed the protein expression levels of SPOP and PD-L1 in tumor biopsies from colon and NSCLC patients treated with PD-1 mAb using immunohistochemistry **(**Fig. 8E, Table S4-S5) and IF staining **(**Fig. 8F and Figure S13C-S13D, Table S6-S7). Patients were categorized as responders or non-responders based on their response to PD-1 mAb therapy. Compared with nonresponders, responders presented greater PD-L1 expression and lower SPOP expression in their biopsies. These findings align with previous findings indicating that SPOP is negatively correlated with PD-L1 expression and that patients with elevated PD-L1 levels tend to respond better to immunotherapy. Additionally, radiographic images from two representative cases from our colon and lung cancer patients supported these findings **(**Fig. 8G and H, Table S4-S7). The analysis of tumor diameter changes in all patients, illustrated in Fig. 8I and J, revealed that the reduction in tumor diameter was negatively correlated with SPOP expression.

We then investigated the relationships between SPOP and various immunomodulators. Our results revealed a positive correlation between SPOP and most immunomodulators in COAD, READ, and LUAD (Figure S14A), indicating an increased capacity for antigen presentation and processing in the high-SPOP group. For example, the chemokines CXCL9, CXCL10, and CCR3 were strongly positively correlated with SPOP, suggesting their involvement in recruiting CD8^+^ T cells into the tumor microenvironment. Moreover, we evaluated the infiltration levels of tumor-infiltrating immune cells via two independent algorithms. Consistent with earlier results, SPOP was positively associated with the infiltration levels of CD8^+^ T cells, NK cells, macrophages, and dendritic cells across all datasets (Figure S14A and S14B). Additionally, the activities within the cancer immune cycle serve as a direct and comprehensive reflection of the functions of the chemokine system and other immunomodulators [61, 62]. We observed a positive correlation between SPOP and several key steps of the cancer immunity cycle, including the release of cancer cell antigens (Step 1); antigen presentation (Step 2); immune cell trafficking to tumors (Step 4), such as the recruitment of T cells, CD8^+^ T cells, Th17 cells, and monocytes; and immune cell infiltration into tumors (Step 5) in COAD, READ, or LUAD (Figure S14C). In conclusion, these results indicate that patients with high PD-L1 and low SPOP are more likely to benefit from PAR treatment.

## Discussion

Increasing research is being focused on understanding the regulatory mechanisms of PD-L1 and identifying small molecule compounds that can modulate PD-L1 expression [36]. Here, we identified PAR, a commonly used antidepressant in clinical practice, significantly downregulates PD-L1 protein expression in colorectal and lung cancer cells. Mechanistically, PAR specifically targets the SPOP protein to promote ubiquitin-mediated degradation of PD-L1. In subcutaneous tumor models, PAR effectively remodels the tumor immune microenvironment and suppresses tumor growth by reducing PD-L1 levels, increasing CD8⁺ T-cell infiltration, and diminishing the presence of myeloid-derived suppressor cells (MDSCs) and regulatory T cells (Tregs).

SPOP (Speckle-type POZ protein) is a key substrate-binding adaptor protein of the Cullin 3-RING E3 ubiquitin ligase complex (CRL3) and plays an important role in regulating the stability of PD-L1 [63, 64]. Previous studies have confirmed that SPOP can specifically recognize PD-L1 and mediate its ubiquitination via the E3 ubiquitin ligase complex, thereby promoting the proteasomal degradation of PD-L1 and significantly reducing the levels of PD-L1 on the surface of tumor cells [53, 65]. In this study, we discovered that PAR can target SPOP, thereby promoting the degradation of PD-L1 and inhibiting tumor growth. Compared with traditional PD-1/PD-L1 antibody therapies, the mechanism of action of PAR not only eliminates PD-L1 at the protein level but also effectively overcomes issues such as drug resistance, poor tissue penetration, and membrane protein mutations that are encountered in the clinical application of antibody drugs [14, 66]. Traditional antibody drugs primarily inhibit immune evasion by blocking the interaction between PD-1 and PD-L1, whereas the strategy of targeting SPOP degrades PD-L1 protein, fundamentally reducing its expression on the surface of tumor cells and thereby more effectively activating the antitumor immune response [67, 68].Therefore, targeting SPOP to promote the degradation of PD-L1 represents an emerging and promising antitumor strategy that holds potential for clinical translation in cancer immunotherapy.

Paroxetine (PAR) is an FDA-approved selective serotonin reuptake inhibitor (SSRI) primarily used for treating depression, anxiety, and obsessive-compulsive disorder [69]. Previous research has shown that PAR can exert anti-breast cancer effects by inducing the mitochondrial apoptosis pathway [70]. Studies on SSRIs have suggested that their immunomodulatory effects primarily stem from enhancing serotonin signaling in T cells, which may improve T cell function within the immunosuppressive tumor microenvironment [71]. However, our study reveals that PAR acts through a distinct, tumor‑autonomous mechanism to exert anti‑tumor immune activity—it directly targets PD‑L1 on cancer cells and promotes its ubiquitination and degradation. This process is independent of serotonin reuptake inhibition. Furthermore, our data rule out any significant effect of PAR on other common immune checkpoint molecules such as FGL1 and CD47. In addition, another SSRI, fluoxetine, did not induce comparable downregulation of PD‑L1. Together, these findings delineate a specific and drug‑selective pathway for paroxetine, underscoring its unique potential in cancer immunotherapy. Meanwhile, we report that PAR binds to the Asp130 site of SPOP, triggering PD-L1 degradation and inhibiting tumor growth, indicating that its antitumor effects are associated with immune responses. In the tumor microenvironment, blocking the interaction between PD-L1 on tumor cells and PD-1 on T cells to re-activate T cell-mediated antitumor immunity is a promising clinical cancer treatment strategy [72]. Our animal experimental results demonstrate that PAR not only reduces PD-L1 expression in tumor cells but also significantly increases CD8^+^ T cell infiltration, thereby promoting antitumor immune responses. Moreover, our data reveal that PAR indirectly activates T cells to exert antitumor immune effects by inhibiting the infiltration and accumulation of myeloid-derived suppressor cells (MDSCs) and regulatory T cells (Tregs). Studies have shown that Tregs and MDSCs form an immunosuppressive network by secreting inhibitory cytokines such as IL-10, TGF-β, and IL-35, which significantly weaken the antitumor functions of effector T cells (Teff), natural killer cells (NK cells), and dendritic cells (DCs) [73, 74]. Our research indicates that PAR alleviates the accumulation of MDSCs and Tregs in the tumor microenvironment by inhibiting SPOP-dependent PD-1/PD-L1 interactions, thereby improving the immune microenvironment. Notably, in immunodeficient nude mouse models, the antitumor effects of PAR disappeared, suggesting that its action relies on an intact host immune system. This finding differs from previous studies using intraperitoneal administration, likely due to the lower bioavailability of PAR *via* oral administration, resulting in insufficient local drug concentrations in tumors to directly kill cancer cells [16].

The combination of PD-1 and CTLA-4 immune checkpoint inhibitors has been clinically validated for cancer treatment, leveraging their distinct mechanisms to achieve synergistic antitumor efficacy [19]. In this study, we explored an alternative therapeutic strategy by combining PAR with anti-CTLA-4, substituting anti-PD-1 blockade, in C57BL/6J mice bearing MC38 or Lewis transplanted tumors. Results demonstrated that, compared with PAR or anti-CTLA-4 monotherapy, the PAR/anti-CTLA-4 combination therapy significantly enhanced CD8^+^ T cell infiltration and induced marked tumor regression, accompanied by extensive tumor necrosis. This indicates that PAR has the potential to serve as a anti-PD-1 alternative in the clinic, which is of great significance for overcoming resistance to traditional PD-1/CTLA-4-based regimens. The AOM/DSS model accurately recapitulates the transition from early benign polyps to invasive malignant tumors, providing a reliable experimental basis for evaluating the therapeutic potential of drugs [75]. Our study demonstrated that PAR significantly inhibits AOM/DSS-induced colon cancer in mice, further highlighting its clinical significance. Analysis of the TCGA and GTEx databases revealed that SPOP expression levels are significantly lower in colorectal cancer (COAD/READ) and lung adenocarcinoma (LUAD) tissues compared to corresponding normal tissues. Additionally, in patients receiving immunotherapy, high PD-L1 expression is associated with better therapeutic outcomes, whereas high SPOP expression is linked to poorer prognosis. These findings further suggest that PAR, by targeting SPOP, holds potential clinical value in cancer treatment.

## Conclusions

Our research has uncovered a new application for the commercially available drug PAR. By targeting the SPOP protein, PAR reduces the expression of PD-L1 and alleviates the immunosuppressive effect on T cells, thereby contributing to antitumor therapy. This finding offers a novel perspective on the development and application of immune checkpoint inhibitors and presents a new therapeutic option for patients with colon and lung cancer.

## Supplementary Information

Supplementary Material 1.

Supplementary Material 2.

## Data Availability

The data that support the findings of this study are available from the corresponding author upon reasonable request.

## References

[1] Xu-Monette ZY, Zhou J, Young KH. PD-1 expression and clinical PD-1 Blockade in B-cell lymphomas. Blood. 2018;131:68–83. 29118007 10.1182/blood-2017-07-740993 PMC5755041

[2] Pauken KE, Torchia JA, Chaudhri A, Sharpe AH, Freeman GJ. Emerging concepts in PD-1 checkpoint biology. Semin Immunol. 2021;52:101480. 34006473 10.1016/j.smim.2021.101480 PMC8545711

[3] Chu X, Tian W, Wang Z, Zhang J, Zhou R. Co-inhibition of TIGIT and PD-1/PD-L1 in cancer immunotherapy: mechanisms and clinical trials. Mol Cancer. 2023;22:93. 37291608 10.1186/s12943-023-01800-3 PMC10249258

[4] Tang Q, et al. The role of PD-1/PD-L1 and application of immune-checkpoint inhibitors in human cancers. Front Immunol. 2022;13:964442. 36177034 10.3389/fimmu.2022.964442 PMC9513184

[5] Sun C, Mezzadra R, Schumacher TN. Regulation and function of the PD-L1 checkpoint. Immunity. 2018;48:434–52. 29562194 10.1016/j.immuni.2018.03.014 PMC7116507

[6] Darvin P, Toor SM, Sasidharan Nair V, Elkord E. Immune checkpoint inhibitors: recent progress and potential biomarkers. Exp Mol Med. 2018;50:1–11. 30546008 10.1038/s12276-018-0191-1 PMC6292890

[7] André T, et al. Nivolumab plus low-dose ipilimumab in previously treated patients with microsatellite instability-high/mismatch repair-deficient metastatic colorectal cancer: 4-year follow-up from checkmate 142. Ann Oncol. 2022;33:1052–60. 35764271 10.1016/j.annonc.2022.06.008

[8] Li J, et al. Biomarkers of pathologic complete response to neoadjuvant immunotherapy in mismatch Repair-Deficient colorectal cancer. Clin Cancer Res. 2024;30:368–78. 37906636 10.1158/1078-0432.CCR-23-2213

[9] Lin A, Wei T, Meng H, Luo P, Zhang J. Role of the dynamic tumor microenvironment in controversies regarding immune checkpoint inhibitors for the treatment of non-small cell lung cancer (NSCLC) with EGFR mutations. Mol Cancer. 2019;18:139. 31526368 10.1186/s12943-019-1062-7 PMC6745797

[10] Dunn GP, Old LJ, Schreiber RD. The immunobiology of cancer immunosurveillance and immunoediting. Immunity. 2004;21:137–48. 15308095 10.1016/j.immuni.2004.07.017

[11] Ettinger DS, et al. Non-small cell lung cancer, version 2.2013. J Natl Compr Canc Netw. 2013;11:645–53. quiz 653. 23744864 10.6004/jnccn.2013.0084

[12] Pitt JM, et al. Resistance mechanisms to Immune-Checkpoint Blockade in cancer: Tumor-Intrinsic and -Extrinsic factors. Immunity. 2016;44:1255–69. 27332730 10.1016/j.immuni.2016.06.001

[13] Awadasseid A, Wu Y, Zhang W. Advance investigation on synthetic small-molecule inhibitors targeting PD-1/PD-L1 signaling pathway. Life Sci. 2021;282:119813. 34256042 10.1016/j.lfs.2021.119813

[14] Wu Q, Jiang L, Li SC, He QJ, Yang B, Cao J. Small molecule inhibitors targeting the PD-1/PD-L1 signaling pathway. Acta Pharmacol Sin. 2021;42:1–9. 32152439 10.1038/s41401-020-0366-x PMC7921448

[15] Meyer JH, et al. The effect of Paroxetine on 5-HT(2A) receptors in depression: an [(18)F]setoperone PET imaging study. Am J Psychiatry. 2001;158:78–85. 11136637 10.1176/appi.ajp.158.1.78

[16] Jang WJ, Jung SK, Vo TTL, Jeong CH. Anticancer activity of Paroxetine in human colon cancer cells: involvement of MET and ERBB3. J Cell Mol Med. 2019;23:1106–15. 30421568 10.1111/jcmm.14011 PMC6349215

[17] Wang T, et al. Reversing T cell dysfunction to boost glioblastoma immunotherapy by Paroxetine-Mediated GRK2 Inhibition and Blockade of multiple checkpoints through biomimetic nanoparticles. Adv Sci (Weinh). 2023;10:e2204961. 36698265 10.1002/advs.202204961 PMC10037995

[18] Wang Q, et al. Paroxetine alleviates T lymphocyte activation and infiltration to joints of collagen-induced arthritis. Sci Rep. 2017;7:45364. 28349925 10.1038/srep45364 PMC5368980

[19] Zhang R et al. D-mannose facilitates immunotherapy and radiotherapy of triple-negative breast cancer via degradation of PD-L1. Proc Natl Acad Sci USA. 2022;119(8):e2114851119. 10.1073/pnas.2114851119 PMC8872783 35181605

[20] Xia J, et al. 5,7,4’-Trimethoxyflavone triggers cancer cell PD-L1 ubiquitin-proteasome degradation and facilitates antitumor immunity by targeting HRD1. MedComm. 2020;5:e611. (2024). 10.1002/mco2.611 PMC11208742 38938284

[21] Ding L et al. Canagliflozin primes antitumor immunity by triggering PD-L1 degradation in endocytic recycling. J Clin Invest. 2023;133(1):e154754. 10.1172/JCI154754 PMC9797339 36594471

[22] Zhuang M, et al. Structures of SPOP-substrate complexes: insights into molecular architectures of BTB-Cul3 ubiquitin ligases. Mol Cell. 2009;36:39–50. 19818708 10.1016/j.molcel.2009.09.022 PMC2847577

[23] Hettich M, Braun F, Bartholomä MD, Schirmbeck R, Niedermann G. High-Resolution PET imaging with therapeutic Antibody-based PD-1/PD-L1 checkpoint tracers. Theranostics. 2016;6:1629–40. 27446497 10.7150/thno.15253 PMC4955062

[24] Liu Y, et al. Berberine diminishes cancer cell PD-L1 expression and facilitates antitumor immunity via inhibiting the deubiquitination activity of CSN5. Acta Pharm Sinica B. 2020;10:2299–312. 10.1016/j.apsb.2020.06.014 PMC7745128 33354502

[25] Gong Y, et al. PUMILIO proteins promote colorectal cancer growth via suppressing p21. Nat Commun. 2022;13:1627. 35338151 10.1038/s41467-022-29309-1 PMC8956581

[26] Hu J, et al. Siglec15 shapes a non-inflamed tumor microenvironment and predicts the molecular subtype in bladder cancer. Theranostics. 2021;11:3089–108. 33537076 10.7150/thno.53649 PMC7847675

[27] Li T, et al. A web server for comprehensive analysis of Tumor-Infiltrating immune cells. Cancer Res. 2017;77:e108–10. 29092952 10.1158/0008-5472.CAN-17-0307 PMC6042652

[28] Becht E, et al. Estimating the population abundance of tissue-infiltrating immune and stromal cell populations using gene expression. Genome Biol. 2016;17:218. 27765066 10.1186/s13059-016-1070-5 PMC5073889

[29] Kovács SA, Fekete JT, Győrffy B. Predictive biomarkers of immunotherapy response with Pharmacological applications in solid tumors. Acta Pharmacol Sin. 2023;44:1879–89. 37055532 10.1038/s41401-023-01079-6 PMC10462766

[30] Wang Q, et al. Benzosceptrin C induces lysosomal degradation of PD-L1 and promotes antitumor immunity by targeting DHHC3. Cell Rep Med. 2024;5:101357. 38237597 10.1016/j.xcrm.2023.101357 PMC10897506

[31] Kumar V, Patel S, Tcyganov E, Gabrilovich DI. The nature of Myeloid-Derived suppressor cells in the tumor microenvironment. Trends Immunol. 2016;37:208–20. 26858199 10.1016/j.it.2016.01.004 PMC4775398

[32] Noman MZ, et al. PD-L1 is a novel direct target of HIF-1α, and its Blockade under hypoxia enhanced MDSC-mediated T cell activation. J Exp Med. 2014;211:781–90. 24778419 10.1084/jem.20131916 PMC4010891

[33] Dieterich LC, Ikenberg K, Cetintas T, Kapaklikaya K, Hutmacher C, Detmar M. Tumor-Associated lymphatic vessels upregulate PDL1 to inhibit T-Cell activation. Front Immunol. 2017;8:66. 28217128 10.3389/fimmu.2017.00066 PMC5289955

[34] Richardson KC, Jung K, Matsubara JA, Choy JC, Granville DJ. Granzyme B in aging and age-related pathologies. Trends Mol Med. 2024;30(12):1165–79. 10.1016/j.molmed.2024.07.010 39181801

[35] Chitsamankhun C, et al. Cathepsin C in health and disease: from structural insights to therapeutic prospects. J Transl Med. 2024;22:777. 39164687 10.1186/s12967-024-05589-7 PMC11337848

[36] Yamaguchi H, Hsu JM, Yang WH, Hung MC. Mechanisms regulating PD-L1 expression in cancers and associated opportunities for novel small-molecule therapeutics. Nat Rev Clin Oncol. 2022;19:287–305. 35132224 10.1038/s41571-022-00601-9

[37] Gou Q, et al. PD-L1 degradation pathway and immunotherapy for cancer. Cell Death Dis. 2020;11:955. 33159034 10.1038/s41419-020-03140-2 PMC7648632

[38] Burr ML, et al. CMTM6 maintains the expression of PD-L1 and regulates anti-tumour immunity. Nature. 2017;549:101–5. 28813417 10.1038/nature23643 PMC5706633

[39] Wang H, et al. HIP1R targets PD-L1 to lysosomal degradation to alter T cell-mediated cytotoxicity. Nat Chem Biol. 2019;15:42–50. 30397328 10.1038/s41589-018-0161-x

[40] Mezzadra R, et al. Identification of CMTM6 and CMTM4 as PD-L1 protein regulators. Nature. 2017;549:106–10. 28813410 10.1038/nature23669 PMC6333292

[41] Ren Y, et al. TRAPPC4 regulates the intracellular trafficking of PD-L1 and antitumor immunity. Nat Commun. 2021;12:5405. 34518538 10.1038/s41467-021-25662-9 PMC8438078

[42] Maher CM, et al. Small-Molecule Sigma1 modulator induces autophagic degradation of PD-L1. Mol Cancer Res. 2018;16:243–55. 29117944 10.1158/1541-7786.MCR-17-0166

[43] Qian G, et al. Membrane-Associated RING-CH 8 functions as a novel PD-L1 E3 ligase to mediate PD-L1 degradation induced by EGFR inhibitors. Mol Cancer Res. 2021;19:1622–34. 34183449 10.1158/1541-7786.MCR-21-0147 PMC8492505

[44] Behera A, Sachan D, Barik GK, Reddy ABM. Role of MARCH E3 ubiquitin ligases in cancer development. Cancer Metastasis Rev. 2024;43(4):1257–77. 10.1007/s10555-024-10201-x 39037545

[45] Qi W et al. The critical role of BTRC in hepatic steatosis as an ATGL E3 ligase. J Mol Cell Biol. 2024;15(10):mjad064. 10.1093/jmcb/mjad064 PMC10993717 37873692

[46] Deng R, et al. MAPK1/3 kinase-dependent ULK1 degradation attenuates mitophagy and promotes breast cancer bone metastasis. Autophagy. 2021;17:3011–29. 33213267 10.1080/15548627.2020.1850609 PMC8526010

[47] Wang D, et al. YAP promotes the activation of NLRP3 inflammasome via blocking K27-linked polyubiquitination of NLRP3. Nat Commun. 2021;12:2674. 33976226 10.1038/s41467-021-22987-3 PMC8113592

[48] Fan Y, et al. CircNR3C2 promotes HRD1-mediated tumor-suppressive effect via sponging miR-513a-3p in triple-negative breast cancer. Mol Cancer. 2021;20:25. 33530981 10.1186/s12943-021-01321-x PMC7851937

[49] Doroudgar S, et al. Hrd1 and ER-Associated protein Degradation, ERAD, are critical elements of the adaptive ER stress response in cardiac myocytes. Circ Res. 2015;117:536–46. 26137860 10.1161/CIRCRESAHA.115.306993 PMC4670262

[50] Li K, et al. Hrd1-mediated ACLY ubiquitination alleviate NAFLD in db/db mice. Metabolism. 2021;114:154349. 32888949 10.1016/j.metabol.2020.154349

[51] Yamasaki S, et al. Cytoplasmic destruction of p53 by the Endoplasmic reticulum-resident ubiquitin ligase ‘Synoviolin’. Embo J. 2007;26:113–22. 17170702 10.1038/sj.emboj.7601490 PMC1782373

[52] Wu Y, et al. ARIH1 signaling promotes anti-tumor immunity by targeting PD-L1 for proteasomal degradation. Nat Commun. 2021;12:2346. 33879767 10.1038/s41467-021-22467-8 PMC8058344

[53] Zhang J, et al. Cyclin D-CDK4 kinase destabilizes PD-L1 via Cullin 3-SPOP to control cancer immune surveillance. Nature. 2018;553:91–5. 29160310 10.1038/nature25015 PMC5754234

[54] Tang Z, et al. ATR Inhibition induces CDK1-SPOP signaling and enhances Anti-PD-L1 cytotoxicity in prostate cancer. Clin Cancer Res. 2021;27:4898–909. 34168048 10.1158/1078-0432.CCR-21-1010 PMC8456453

[55] Geng C, et al. SPOP mutations target STING1 signaling in prostate cancer and create therapeutic vulnerabilities to PARP Inhibitor-Induced growth suppression. Clin Cancer Res. 2023;29:4464–78. 37581614 10.1158/1078-0432.CCR-23-1439 PMC11017857

[56] Martinez Molina D, et al. Monitoring drug target engagement in cells and tissues using the cellular thermal shift assay. Science. 2013;341:84–7. 23828940 10.1126/science.1233606

[57] Tu Y, Tan L, Tao H, Li Y, Liu H. CETSA and thermal proteome profiling strategies for target identification and drug discovery of natural products. Phytomedicine. 2023;116:154862. 37216761 10.1016/j.phymed.2023.154862

[58] Sohn OS, Fiala ES, Requeijo SP, Weisburger JH, Gonzalez FJ. Differential effects of CYP2E1 status on the metabolic activation of the colon carcinogens azoxymethane and Methylazoxymethanol. Cancer Res. 2001;61:8435–40. 11731424

[59] Gobert AP, et al. Protective role of spermidine in colitis and colon carcinogenesis. Gastroenterology. 2022;162:813–e827818. 34767785 10.1053/j.gastro.2021.11.005 PMC8881368

[60] Wei SC, et al. Distinct cellular mechanisms underlie Anti-CTLA-4 and Anti-PD-1 checkpoint Blockade. Cell. 2017;170:1120–e11331117. 28803728 10.1016/j.cell.2017.07.024 PMC5591072

[61] Chen DS, Mellman I. Oncology Meets immunology: the cancer-immunity cycle. Immunity. 2013;39:1–10. 23890059 10.1016/j.immuni.2013.07.012

[62] Xu L, et al. TIP: A web server for resolving tumor immunophenotype profiling. Cancer Res. 2018;78:6575–80. 30154154 10.1158/0008-5472.CAN-18-0689

[63] Zhang H, Jin X, Huang H. Deregulation of SPOP in cancer. Cancer Res. 2023;83:489–99. 36512624 10.1158/0008-5472.CAN-22-2801

[64] Zhou Q, et al. Neddylation Inhibition induces glutamine uptake and metabolism by targeting CRL3(SPOP) E3 ligase in cancer cells. Nat Commun. 2022;13:3034. 35641493 10.1038/s41467-022-30559-2 PMC9156729

[65] Gao K, et al. SPOP mutations promote tumor immune escape in endometrial cancer via the IRF1-PD-L1 axis. Cell Death Differ. 2023;30:475–87. 36481790 10.1038/s41418-022-01097-7 PMC9950446

[66] Dong M, Qian M, Ruan Z. CUL3/SPOP complex prevents immune escape and enhances chemotherapy sensitivity of ovarian cancer cells through degradation of PD-L1 protein. J Immunother Cancer. 2022;10(10):e005270. 10.1136/jitc-2022-005270 PMC9535172 36198437

[67] Liu C, Seeram NP, Ma H. Small molecule inhibitors against PD-1/PD-L1 immune checkpoints and current methodologies for their development: a review. Cancer Cell Int. 2021;21:239. 33906641 10.1186/s12935-021-01946-4 PMC8077906

[68] Yi M, Zheng X, Niu M, Zhu S, Ge H, Wu K. Combination strategies with PD-1/PD-L1 blockade: current advances and future directions. Mol Cancer. 2022;21:28. 35062949 10.1186/s12943-021-01489-2 PMC8780712

[69] Wang B, et al. Paroxetine is an effective treatment for refractory erythema of rosacea: primary results from the prospective rosacea refractory erythema randomized clinical trial. J Am Acad Dermatol. 2023;88:1300–7. 36806645 10.1016/j.jaad.2023.01.044

[70] Cho YW et al. Paroxetine induces apoptosis of human breast cancer MCF-7 cells through Ca(2+)-and p38 MAP Kinase-Dependent ROS generation. Cancers (Basel). 2019;11(1):64. 10.3390/cancers11010064 PMC6356564 30634506

[71] Li B, et al. Serotonin transporter inhibits antitumor immunity through regulating the intratumoral serotonin axis. Cell. 2025;188:3823–e38423821. 40403728 10.1016/j.cell.2025.04.032 PMC12255530

[72] Pang K, et al. Research progress of therapeutic effects and drug resistance of immunotherapy based on PD-1/PD-L1 Blockade. Drug Resist Updat. 2023;66:100907. 36527888 10.1016/j.drup.2022.100907

[73] Cervantes-Villagrana RD, Albores-García D, Cervantes-Villagrana AR, García-Acevez SJ. Tumor-induced neurogenesis and immune evasion as targets of innovative anti-cancer therapies. Signal Transduct Target Therapy. 2020;5:99. 10.1038/s41392-020-0205-z PMC7303203 32555170

[74] Hiam-Galvez KJ, Allen BM, Spitzer MH. Systemic immunity in cancer. Nat Rev Cancer. 2021;21:345–59. 33837297 10.1038/s41568-021-00347-z PMC8034277

[75] Dzhalilova D, Zolotova N, Fokichev N, Makarova O. Murine models of colorectal cancer: the azoxymethane (AOM)/dextran sulfate sodium (DSS) model of colitis-associated cancer. PeerJ. 2023;11:e16159. 37927787 10.7717/peerj.16159 PMC10624171

