# Supplementary material for: Paroxetine repurposing enhances antitumor immunity via SPOP-mediated PD-L1 ubiquitination and proteasomal degradation
Source: J Exp Clin Cancer Res. 2026 Jan 27;45:57. doi: 10.1186/s13046-026-03648-z (PMC12918487; doi:10.1186/s13046-026-03648-z)
Supplement: Supplementary file 1 — Supplementary Material 1. [file 13046_2026_3648_MOESM1_ESM.docx]

Supplementary Materials for

**Paroxetine repurposing enhances antitumor immunity via SPOP-mediated PD-L1 ubiquitination and proteasomal degradation**

Mengting Xu^1†^, Saisai Tian^2†^, Hanchi Xu^1†^, Xinying Xue^3^, Qing Zhang^1^, Hongmei Hu^1^, Gaosong Wu^1^, Xiangxin Geng^1^, Dianping Yu^1^, Hanchen Xu^4^, Mei Xie^3^, Linyang Li^1^, Xinru Li^1^, Simeng Li^1^, Shize Xie^2^, Xuwen Lin^3^, Shuzhen Lyu^5^, Yutong Xie^6^, Biao Zhang^7*^, Haiyang Zhou^8^*, Qun Wang^1*^, Weidong Zhang^2,9*^, Sanhong Liu^1,10*^

^†^ Authors contributed equally to this work

Corresponding to: Sanhong Liu [(liush@shutcm.edu.cn),](mailto:(liush@shutcm.edu.cn),) Weidong Zhang [(wdzhangy@hotmail.com)](mailto:(wdzhangy@hotmail.com),), Qun Wang (qunwang0523@163.com), Haiyang Zhou (haiyang1985_1@aliyun.com), Biao Zhang (zhangb@huitianjinze.com)

**This PDF file includes:**

Figures S1 to S14

Tables S1 to S7

**
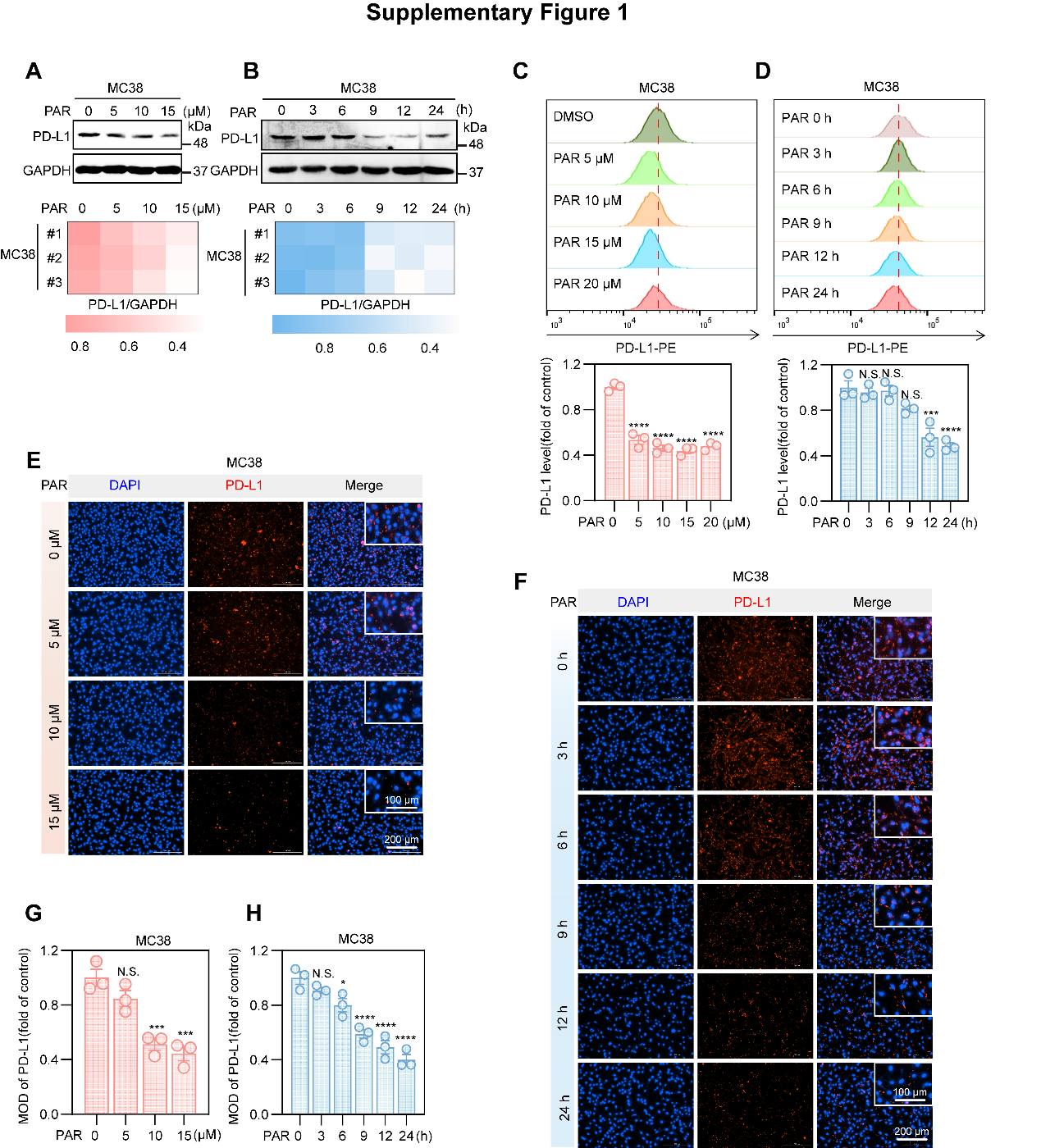
**

**Figure S1. PAR downregulates PD-L1 levels in MC38 cells.** (**A and B**) Western blotting analysis of PD-L1 protein expression in MC38 cells treated with PAR at different concentrations (A) or for different durations (B). (**C and D**) The effects of the PAR concentration (C) or time (D) on PD-L1 expression on the MC38 cell membrane were analyzed *via* flow cytometry. (**E-H**) Expression of membrane PD-L1 detected by immunofluorescence after treatment with different concentrations of PAR (E) or 10 μM PAR for 24 h (F) in MC38 cells. DAPI staining in blue revealed cell nucleus, while red fluorescence indicated PD-L1 on cell membranes, and the scale bar represents 200 μm. (G) and (H) Quantitative statistical plots of (E) and (F), respectively. Data are presented as mean ± SEM. Statistical significance was determined by one-ANOVA with Dunnett's test (*p < 0.05, **p < 0.01, ***p < 0.001, ****p < 0.0001; N.S., not significant).


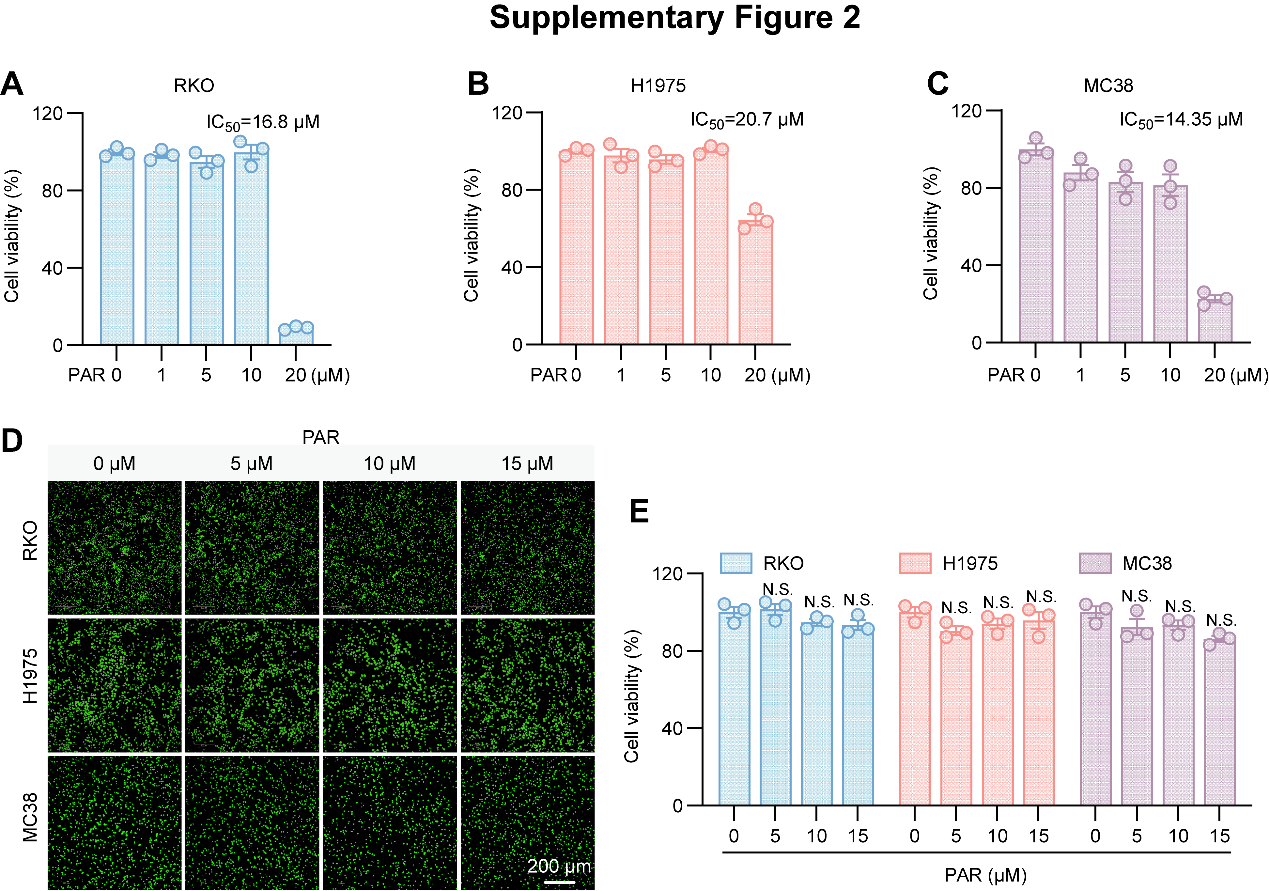


**Figure S2. Assessing the cytotoxicity of PAR in cancer cells.** (**A-C**) A CCK-8 assay was used to evaluate the inhibitory effects of PAR on RKO (A), H1975 (B), and MC38 (C) cells. (**D**) An EdU kit was used to determine the toxicity of PAR at various concentrations in RKO, H1975, and MC38 cells. (**E**) Graphical representation of the quantification of the green fluorescence intensity in (D). Data are presented as mean ± SEM. Statistical significance was determined by one-ANOVA with Dunnett's test (*p < 0.05, **p < 0.01, ***p < 0.001, ****p < 0.0001; N.S., not significant).


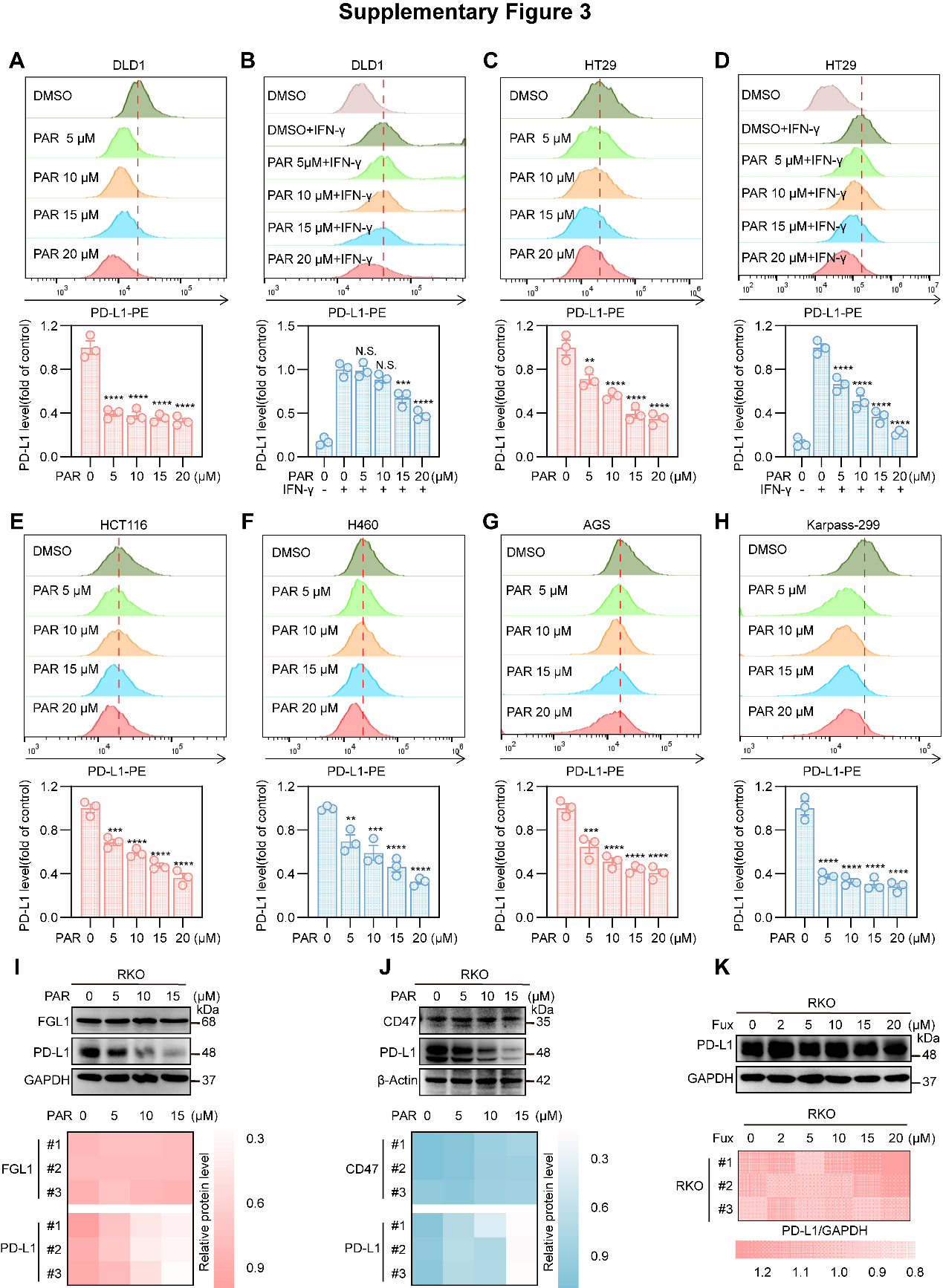


**Figure S3. PAR broadly and selectively downregulates PD-L1. (A-H)** Flow cytometry analysis of PD‑L1 expression (median fluorescence intensity, MFI) in various cancer cell lines treated with increasing concentrations of PAR: colorectal cancer lines DLD1 (A), IFN‑γ‑stimulated DLD1 (B), HT29 (C), IFN‑γ‑stimulated HT29 (D), HCT116 (E), lung cancer H460 (F), gastric cancer AGS (G), and anaplastic large‑cell lymphoma Karpas‑299 (H). **(I-J)** Western blot analysis of FGL1 (I) and CD47 (J) protein levels in RKO cells after 24 h treatment with 0, 5, 10, or 15 μM PAR; quantification showed no significant downregulation of either protein. **(K)** PD‑L1 protein expression in RKO cells treated with fluoxetine (0, 2, 5, 10, 15, 20 μM) for 24 h, assessed by Western blot; no marked reduction was observed. Statistical significance was determined by one-ANOVA with Dunnett’s test (*p < 0.05, **p < 0.01, ***p < 0.001, ****p < 0.0001; N.S., not significant).


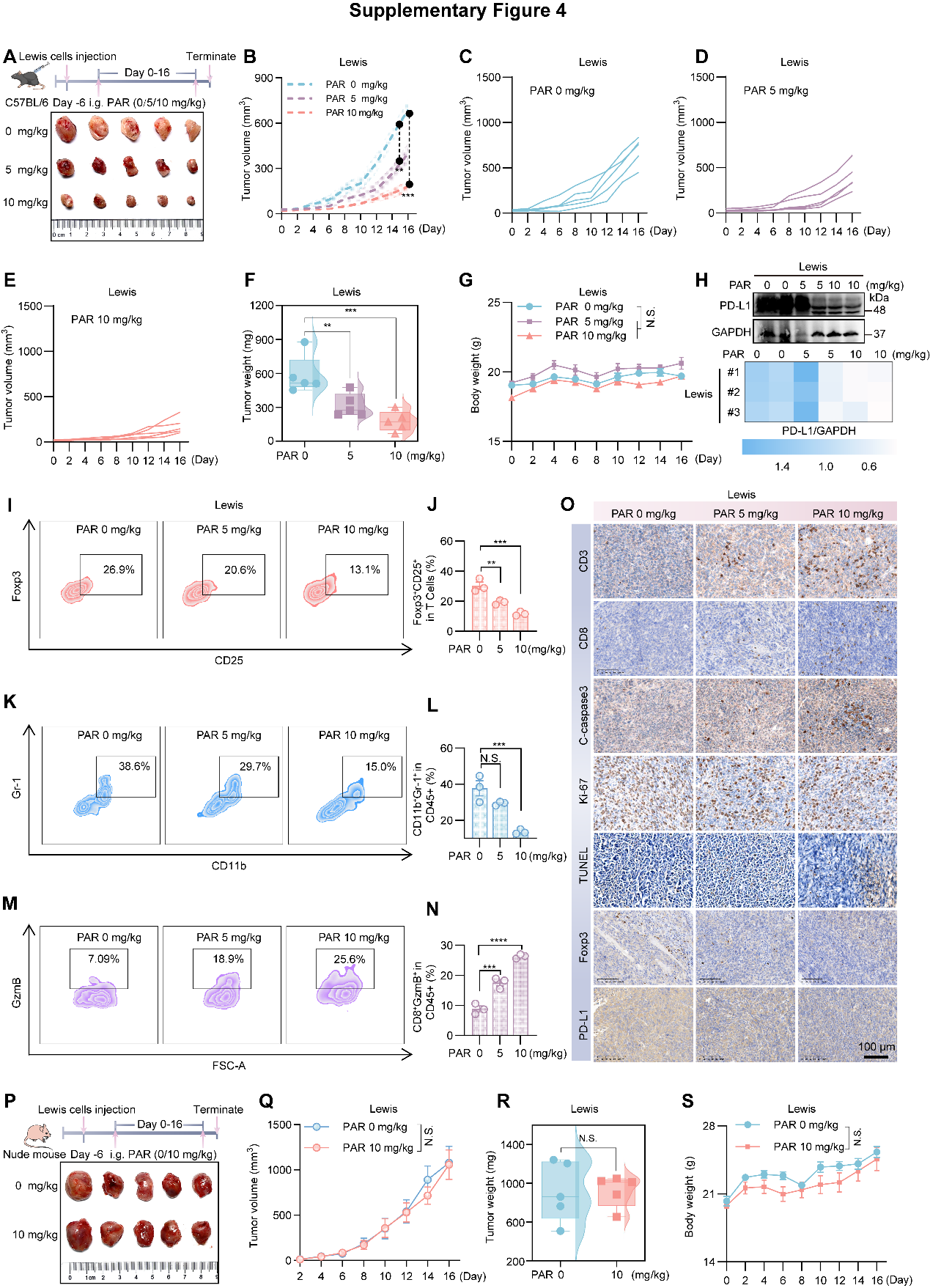


**Figure S4. PAR inhibits the growth of lung cancer *in vivo*** **primarily by activating T cell immunity.** C57BL/6J (female) mice were injected subcutaneously with Lewis lung cancer cells (3 × 10^7^ cells/mouse) and treated by gavage with PAR dissolved in corn oil; n = 5 mice per group. (**A**) Schematic diagram of the experimental procedure used to establish the transplanted tumor model of lung cancer and representative solid tumors excised from different groups of mice. (**B-E**) (B) Tumor growth curves of different groups of mice. (C), (D) and (E) Tumor growth curves of mice administered 0 mg/kg, 5 mg/kg or 10 mg/kg PAR, respectively. (**F**) Tumor weights of the mice in the different groups. (**G**) Changes in the body weights of the mice in different groups during drug administration were recorded. (**H**) PD-L1 expression in the tumor tissues of the mice in the different treatment groups was detected via western blotting, and the results were quantified. (**I-N**) Flow cytometry analysis of CD4^+^CD25^+^Foxp3^+^ (I), CD11b^+^Gr-1^+^ (K), and CD8^+^GzmB^+^ (M), which represent Treg cells, MDSCs, and granzymes, respectively, in the subcutaneous Lewis graft tumor tissues of the mice treated with different doses of PAR; the results are quantified in (J), (L), and (N), respectively. (**O**) Immunohistochemical staining showing the expression of CD3, CD8, C-caspase-3, Ki-67, TUNEL, Foxp3 and PD-L1 in Lewis tumor tissues from different groups of mice; scale bar, 100 μm. (**P-S**) Subcutaneous tumor models were created by injecting 3 × 10^7^ Lewis lung cancer cells into the axilla of female nude mice. The mice received oral treatment with either 0 mg/kg or 10 mg/kg PAR, with 5 mice per group. The study includes (P) a schematic of the experimental procedure and representative solid tumors from the different dosing groups, (Q) growth curves of Lewis transplanted tumors across the dosing groupI(R) statistical graphs of tumor weights in the transplanted tumors, and (S) changes in body weights of the nude mice on the basis of PAR dosage. Data are presented as mean ± SEM. Statistical significance was determined by ANOVA with D’nnett's test (*p < 0.05, **p < 0.01, ***p < 0.001, ****p < 0.0001; N.S., not significant).

**
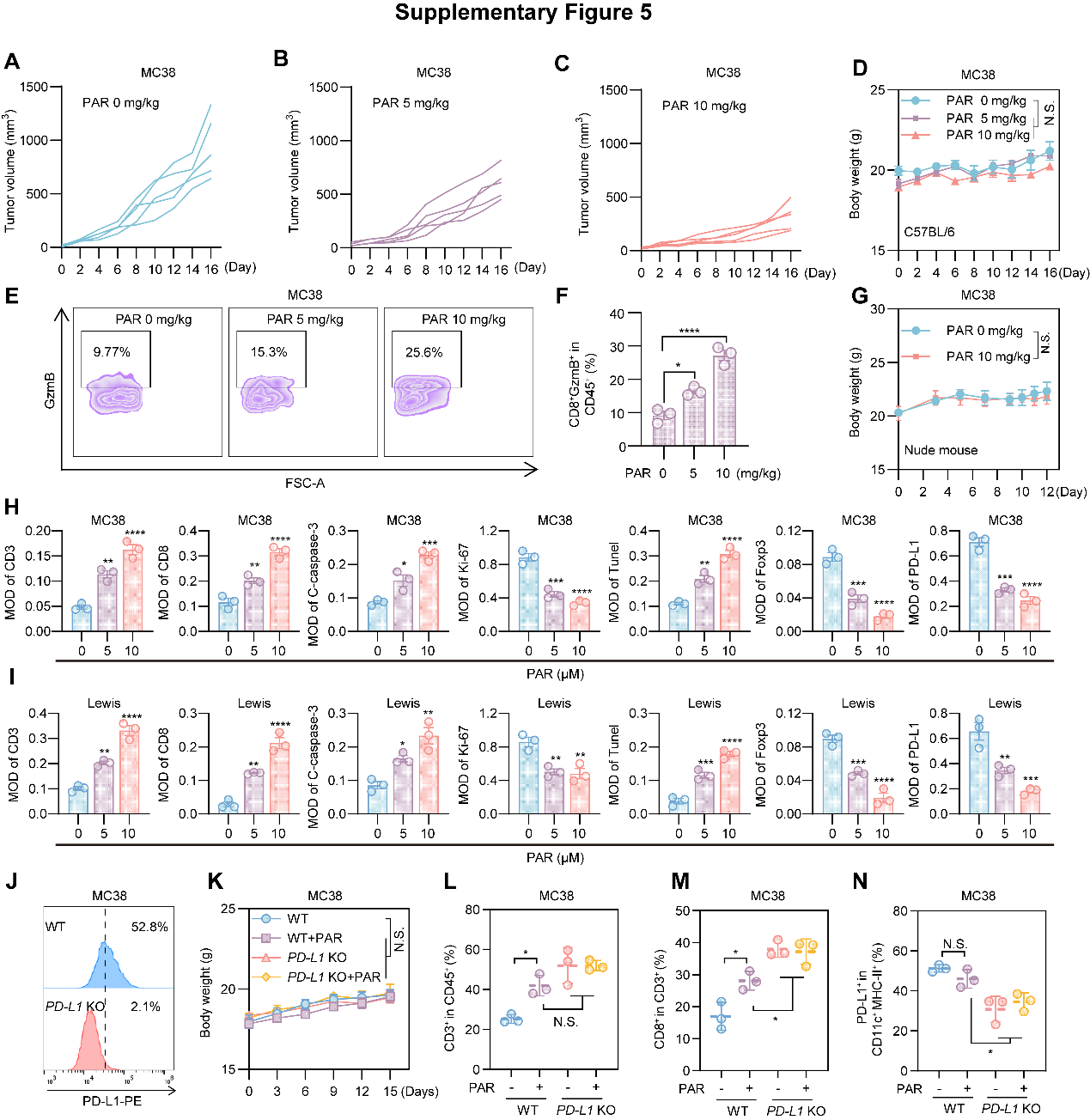
**

**Figure S5. PAR exerts antitumor effects by relieving immune suppression. (A-F)** Female C57BL/6J mice were subcutaneously inoculated with MC38 colon cancer cells (8 × 10⁵ cells per mouse) and administered PAR dissolved in corn oil by oral gavage. Tumor growth curves are shown for mice treated with (A) 0 mg/kg, (B) 5 mg/kg, or (C) 10 mg/kg PAR. (D) Body weight changes of mice in each group during treatIt. (E) Flow cytometry analysis of CD8⁺GzmB⁺ cytotoxic T cells in MC38 allografts from mice treated with different doses of PAR. (F) Quantitative result of (E). **(G)** Subcutaneous tumor models were established in female nude mice by axillary injection of MC38 colorectal cancer cells (8 × 10⁵ cells per mouse). Body weight changes are shown for nude mice orally treated with 0 mg/kg or 10 mg/kg PAR. **(H, I)** Immunohistochemical quantification of CD3, CD8, cleaved caspase-3, Ki-67, TUNEL, Foxp3, and PD-L1 expression in tumor tissues, with results corresponding to Figure 2I and Figure S4O, respectively. **(J)** Flow cytometry verification of PD-L1 knockout efficiency in MC38 cells. **(K-N)** C57BL/6 mice were subcutaneously injected with 1 × 10⁶ wild-type or PD-L1-knockout MC38 cells and treated with PAR or vehicle control. (K) Body weight changes across treatment groups. (L-N) Flow cytometric analysis of tumor-infiltrating (L) CD3⁺ T cells, (M) CD8⁺ T cells, and (N) CD11c⁺MHC-II⁺PD-L1⁺ dendritic cells. Data are presented as mean ± SEM. Statistical significance was determined by ANOVA with D’nnett's test (*p < 0.05, **p < 0.01, ***p < 0.001, ****p < 0.0001; N.S., not significant).

**
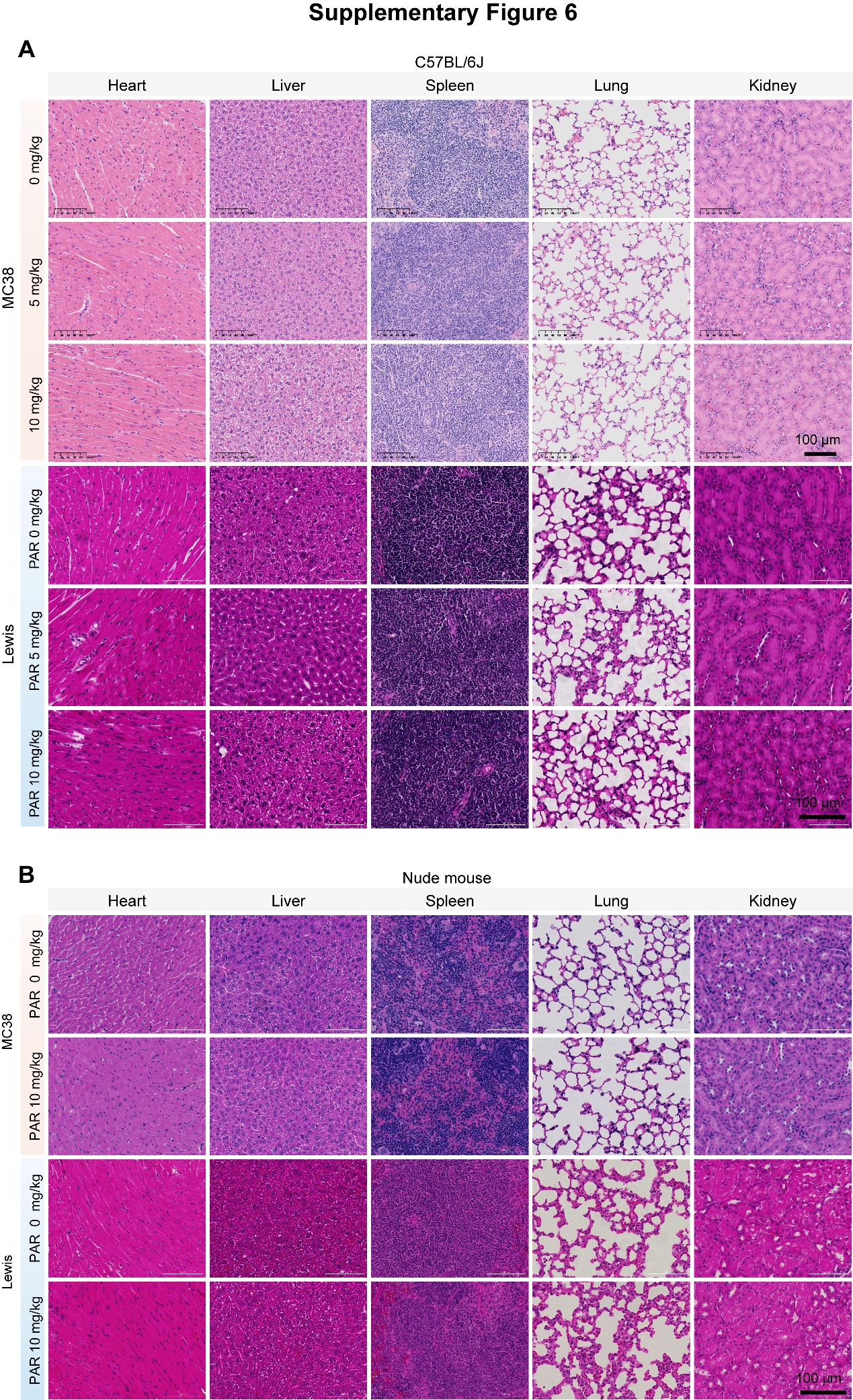
**

**Figure S6.** **PAR has no toxic side effects on mice.** Safety of oral administration of PAR. MC38 colon cancer cells or Lewis lung cancer cells were injected subcutaneously into C57BL/6J (female) or immunodeficient nude mice (female) and treated orally with different doses of PAR. (**A**) H&E staining of the heart, liver, spleen, lungs and kidneys of different groups of C57BL/6J mice. (**B**) H&E staining of the viscera of immunodeficient nude mice from different groups treated with drugs; scale bar, 100 μm.


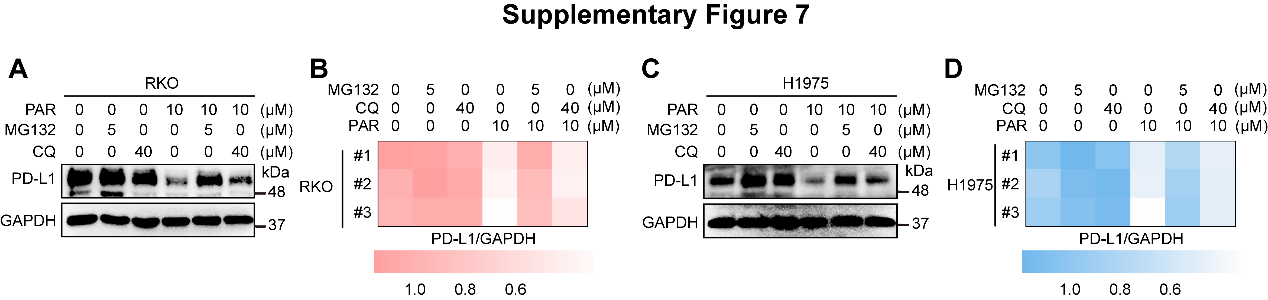


**Figure S7.** **Proteasome inhibitors reverse PAR-induced PD-L1 degradation.** (**A-D**) Comparison of the effects of MG132 and chloroquine on the downregulation of PD-L1 expression via Western blotting in RKO (A) and H1975 (C) cells. (B) and (D) Quantitative statistical plots of (A) and (C), respectively. Data are presented as mean ± SEM. Statistical significance was determined by one-ANOVA with D’nnett's test (*p < 0.05, **p < 0.01, ***p < 0.001, ****p < 0.0001; N.S., not significant).


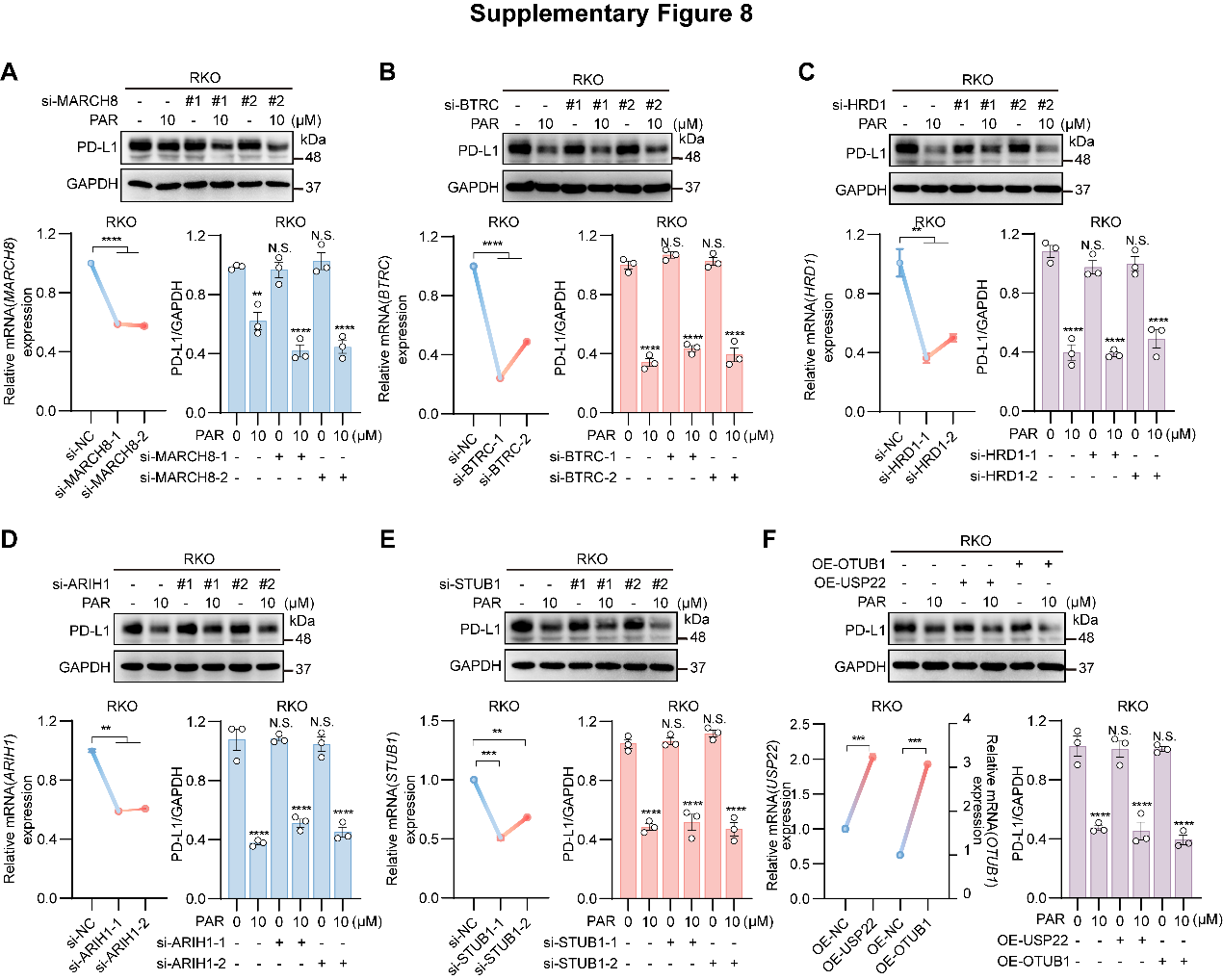


**Figure S8.** **Only SPOP knockdown reversed the downregulation of PD-L1 expression by PAR.** (**A-E**) MARCH8 (A), BTRC (B), HRD1 (C), ARIH1 (D) or IB1 (E) were interfered with by siRNA in RKO cells, and the knockdown efficiency was determined by RT-qPCR. Then, the interfered RKO cells were treated with PAR, and Western blotting was used to detect and quantify the PD-L1 protein. (**F**) To observe whether PD-L1 continues to be downregulated after PAR action on RKO cells overexpressing OTUB1 or USP22, we assessed the knockdown efficiency via RT-qPCR. Data are presented as mean ± SEM. Statistical significance was determined by one-ANOVA wit’ Dunnett's test (*p < 0.05, **p < 0.01, ***p < 0.001, ****p < 0.0001; N.S., not significant).


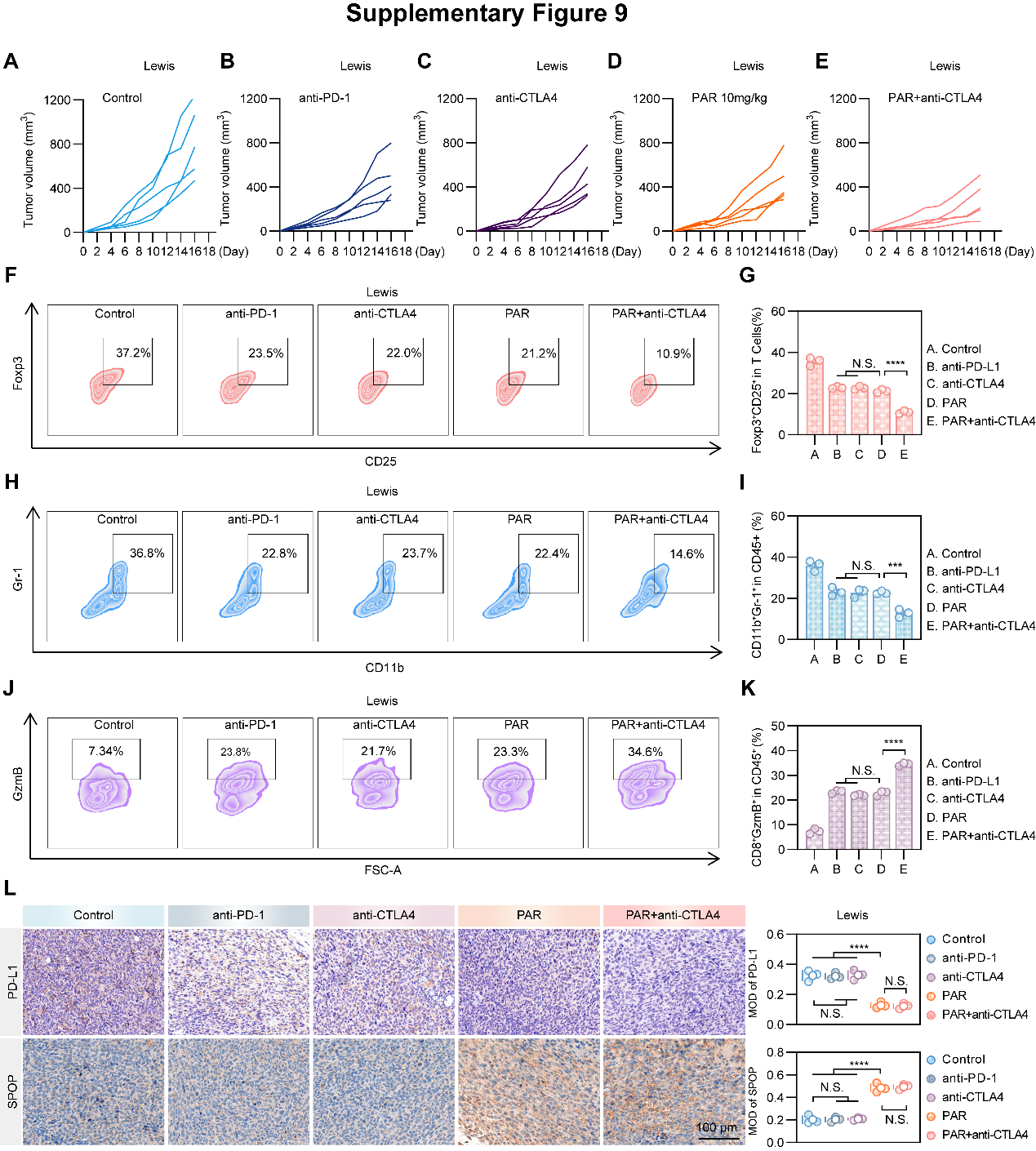


**Figure S9. Combination therapy using PAR and anti-CTLA4 enhances the efficacy in lung cancer.** Lung cancer cells (Lewis, 3 × 10^7^ cells/mouse) were injected subcutaneously into female C57BL/6J mice, which were divided into five groups and treated with corn oil, anti-PD-1, anti-CTLA4, PAR (10 mg/kg), or a combination of PAR with anti-CTLA4. (**A-E**) Growth curves for representative mice in the control group (A), anti-PD-1 (B), anti-CTLA4 (C), PAR (D), and PAR combined with anICTLA4 (E) are shown. (**F-K)** Flow cytometry analysis of the levels of CD4^+^CD25^+^Foxp3^+^ (F), CD11b^+^Gr-1^+^ (H), and CD8^+^GzmB^+^ (J) in Lewis tumor tissues posttreatment, with (G), (I), and (K) providing quantitative results for (F), (H), and (J), respectively. (**L**) Immunohistochemistry results showing PD-L1 and SPOP levels in lung cancer tissues across the treatment groups; scale bar = 100 μm. The data shown are the mean value ± standard error of the mean (SEM). Data are presented as mean ± SEM. Statistical significance was determined by ANOVA ’ith Dunnett's test (*p < 0.05, **p < 0.01, ***p < 0.001, ****p < 0.0001; N.S., not significant).


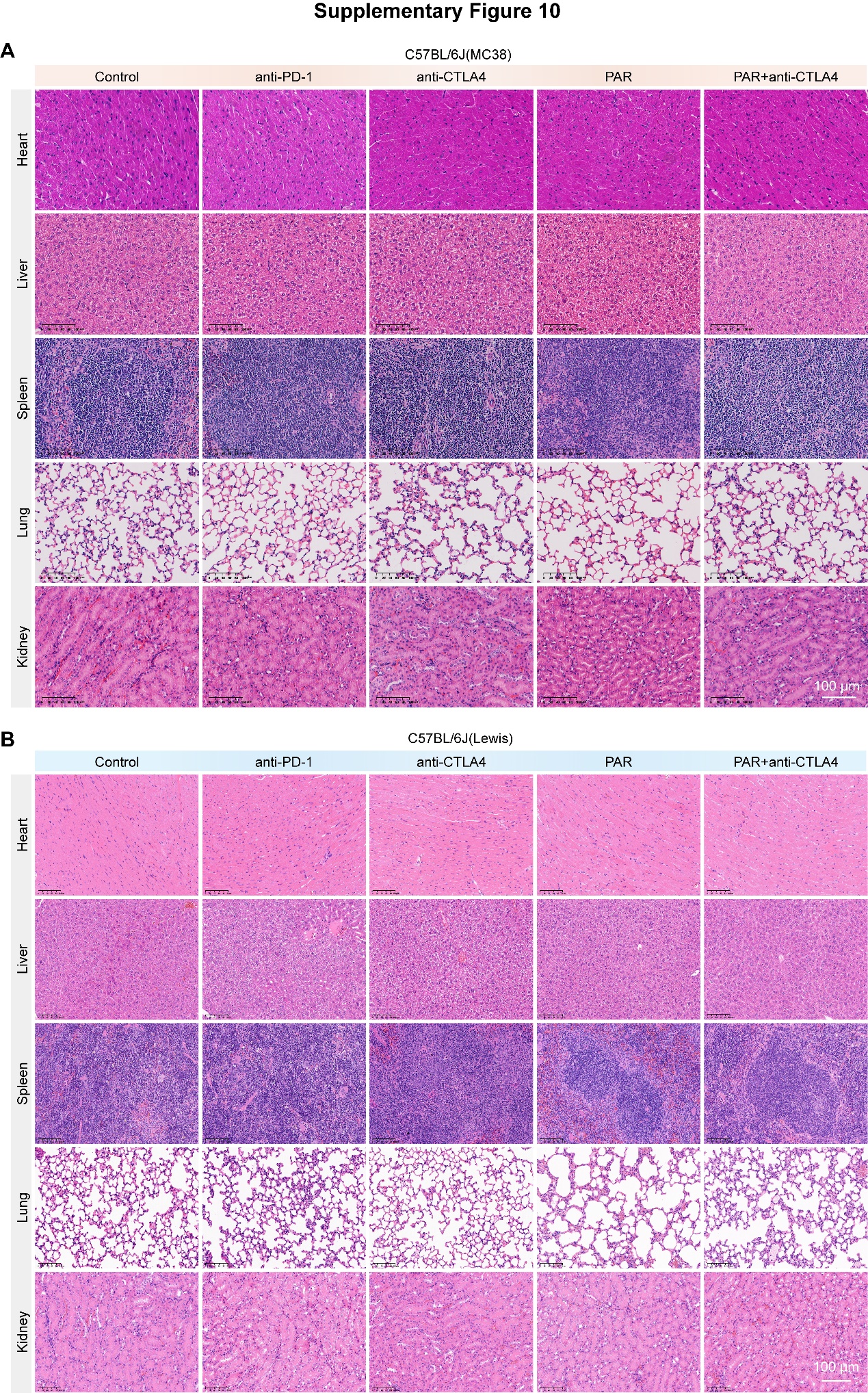


**Figure S10.** **PAR was not toxic to mice.** MC38 colon cancer cells or Lewis lung cancer cells were inoculated subcutaneously into C57BL/6J (female) mice, which were treated with corn oil, anti-PD-1, anti-CTLA4, PAR, or PAR combined with anti-CTLA4. **(A and B**) H&E staining of the heart, liver, spleen, lungs and kidneys of different groups of representative mice with rectal cancer MC38 subcutaneous tumors (A) or lung cancer Lewis subcutaneous tumors (B), scale bar = 100 μm.

**
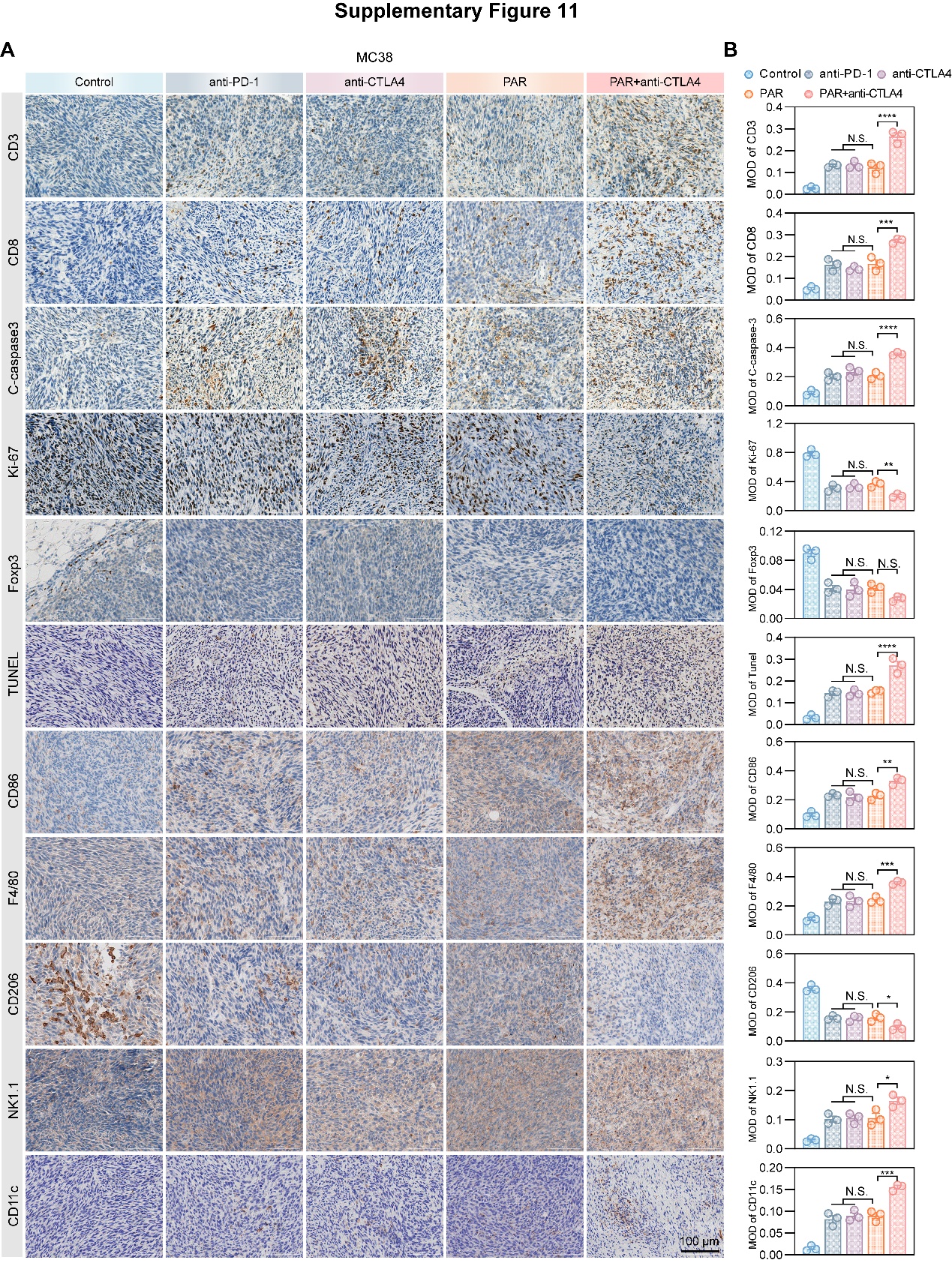
**

**Figure S11. PAR activates the immune microenvironment of colon cancer tumors in mice.** C57BL/6J (female) mice were treated with corn oil, anti-PD-1, anti-CTLA4, PAR, or PAR combined with anti-CTLA4 after subcutaneous injection of MC38 colon cancer cells (8 × 10^5^ cells/mouse). **(A**) Immunohistochemical staining was performed to analyze the expression of CD3, CD8, Ki-67, C-caspase-3, Foxp3, TUNEL, NK1.1, CD206, CD86, F4/80 and CD11c in the tumor tissues of the mice in each group. (**B**) Quantification of (A). Data are presented as mean ± SEM. Statistical significance was determined by one-ANOVA with Dunnett's test (*p < 0.05, **p < 0.01, ***p < 0.001, ****p < 0.0001; N.S., not significant).


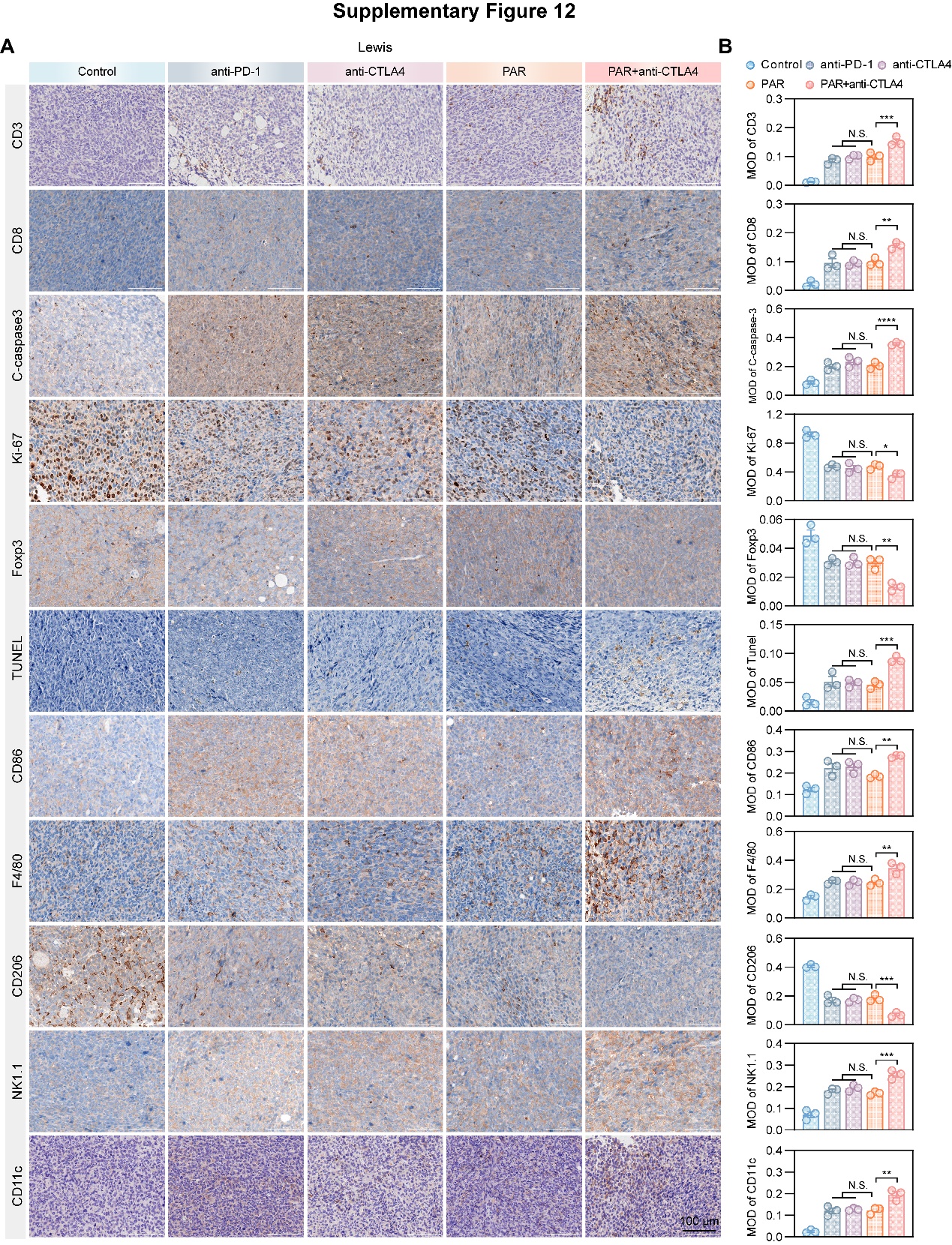


**Figure S12. Enhancement of the lung cancer immune microenvironment by PAR in Mice.** Female C57BL/6J mice were inoculated subcutaneously with Lewis lung cancer cells (3×10^7^ cells/mouse) and subsequently treated with corn oil, anti-PD-1, anti-CTLA4, PAR, or a combination of PAR and anti-CTLA4. (**A**) Immunohistochemical analysis was conducted to assess the expression of the immune markers CD3, CD8, Ki-67, cleaved caspase-3, Foxp3, TUNEL, NK1.1, CD206, CD86, F4/80, and CD11c in tumor tissues across treatment groups. (**B**) Quantitative analysis of the immunohistochemical staining results from (A). Data are presented as mean ± SEM. Statistical significance was determined by ANOVA with Dunnett's test (*p < 0.05, **p < 0.01, ***p < 0.001, ****p < 0.0001; N.S., not significant).


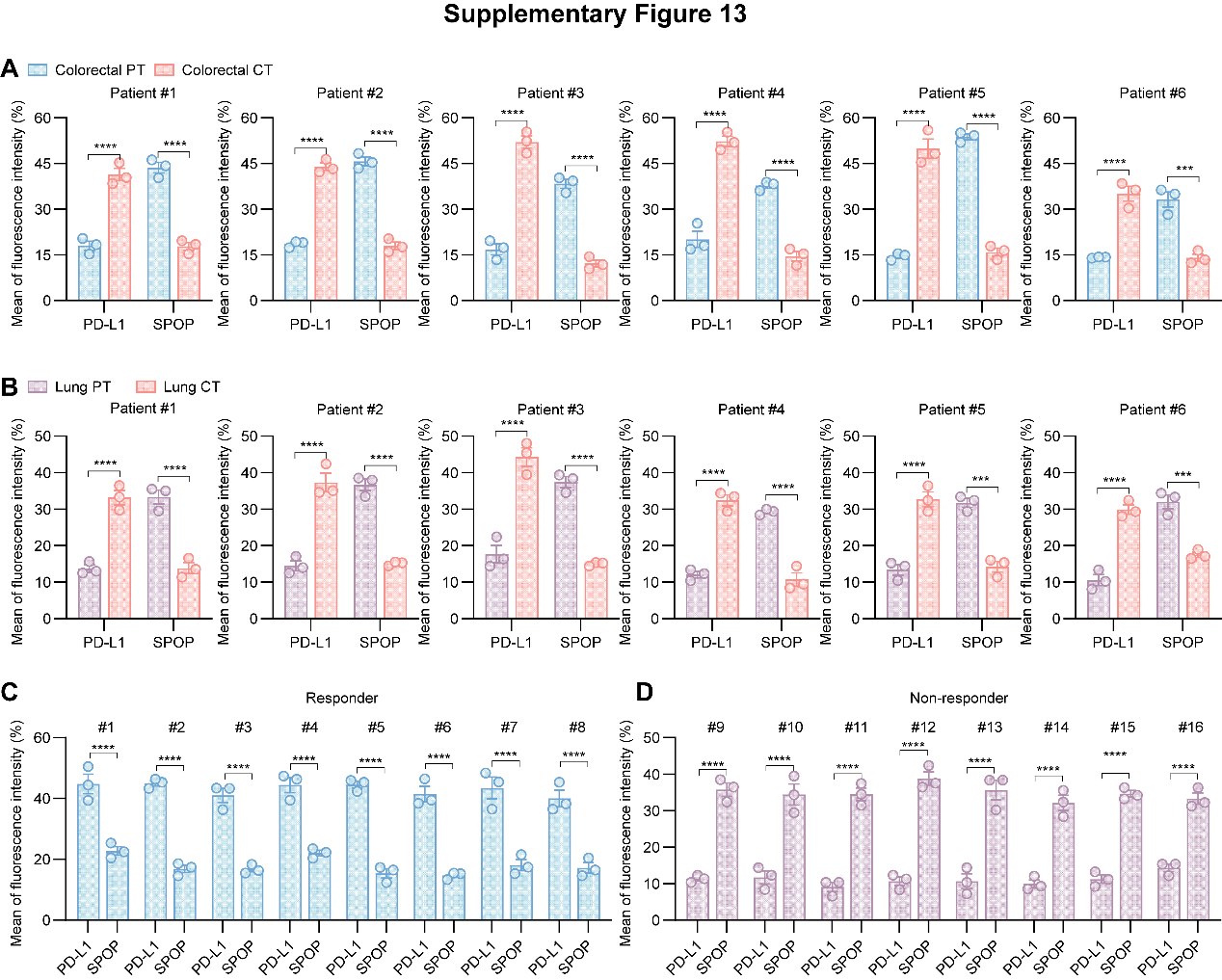


**Figure S13. Clinical correlation of SPOP with colon and lung cancer. (A and B)** (A) and (B) present the quantitative data from Figures 8D. **(C and D)** The quantitative data from Figures 8F. Data are presented as mean ± SEM. Statistical significance was determined by one-ANOVA with Dunnett's test (*p < 0.05, **p < 0.01, ***p < 0.001, ****p < 0.0001; N.S., not significant).


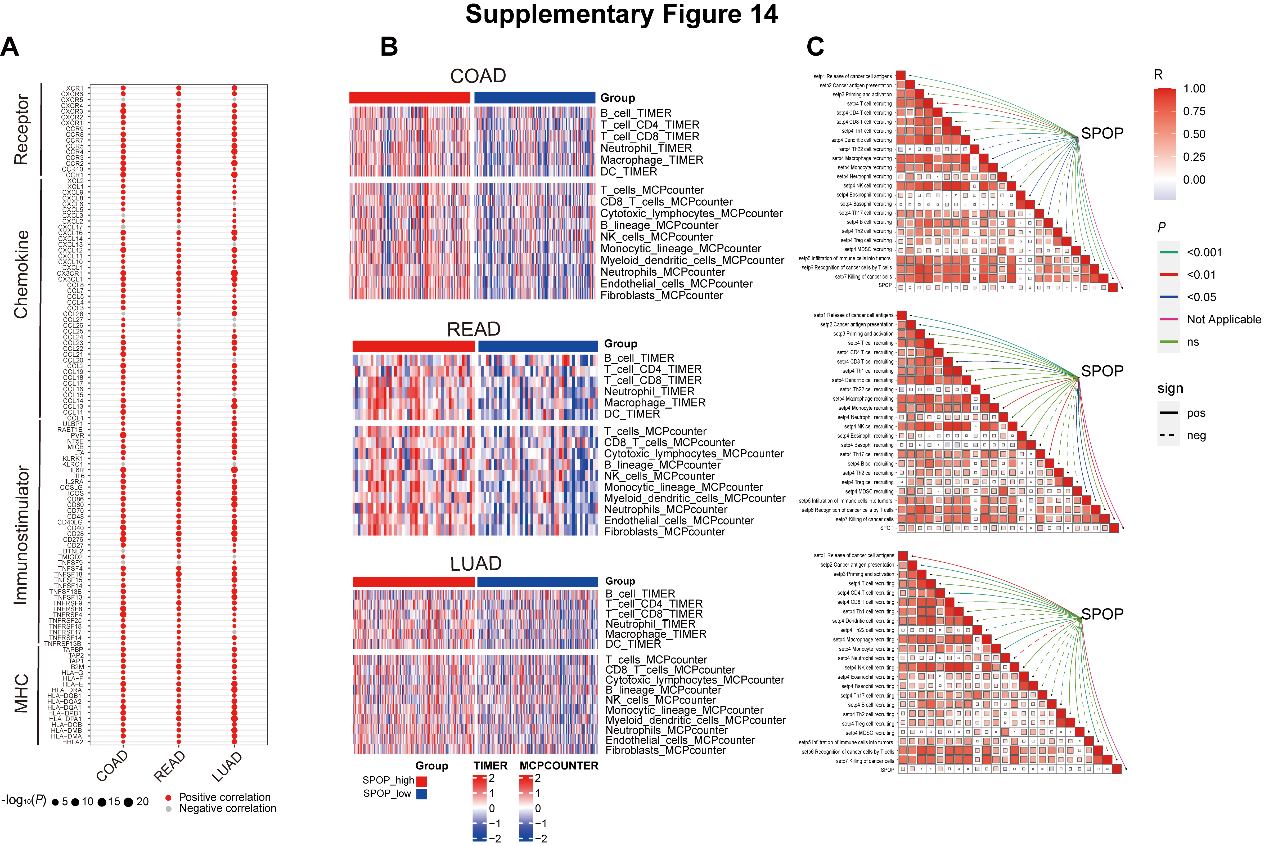


**Figure S14. Relevance of SPOP to the treatment of colon and lung cancer. (A)** Correlations between SPOP and immunomodulators (chemokines, receptors, MHC, and immunostimulators) in COAD, READ and LUAD. **(B)** Differences in the infiltration level of tumor-infiltrating immune cells between the high- and low-SPOP groups were determined via the TIMER and MCPcounter algorithms. **(C)** Correlations between SPOP and the cancer immune cycle in COAD, READ and LUAD.

| **Table S1. Antibody** | | |
| --- | --- | --- |
| **Antibody** | **Source** | **Category No.** |
| Anti-PD-L1 | Proteintech | 66248-1-Ig |
| Anti-PD-L1 | Abcam | ab203103 |
| SPOP | Proteintech | 16750-1-AP |
| Ubiquitin | Abcam | ab7245 |
| Anti-mouse PD-1 (CD279) | Invivogen | BE0146 |
| Anti-mouse CTLA-4(CD152) | Invivogen | BP0032 |
| GAPDH | Proteintech | 60004-1-Ig |
| CD47 | Proteintech | 20305-1-AP |
| FGL1 | Proteintech | 16000-1-AP |
| β-Actin | Proteintech | 66009-1-Ig |
| PE anti-human CD274 | Biolegend | 329706 |
| PE anti-mouse CD274 | Biolegend | 124307 |
| PE anti-mouse CD25 | Biolegend | 101904 |
| Alexa Fluor® 700 anti-human/mouse Granzyme B Recombinant | Biolegend | 372222 |
| Anti-Mouse CD3ε，APC-Cy7 | Multi Sciences | 70-F21003A06-100 |
| Anti-Mouse CD8α，PerCP-Cy5.5 | Multi Sciences | 70-F2100804-100 |
| Anti-Human/Mouse CD11b，mFluor 450 | Multi Sciences | 70-F41011b07-100 |
| Anti-Mouse CD4，PE-Cy7 | Multi Sciences | 70-F2100405/2-100 |
| Anti-Mouse Ly-6G(Gr-1)，FITC | Multi Sciences | 70-F21LY6G01-100 |
| Anti-Mouse Foxp3，APC | Multi Sciences | 70-F21FP303-100 |
| FITC Rat anti-Mouse CD45 Antibody(I3/2.3) | Absin | abs1890030 |
| Percp Rat anti-Mouse CD3 | Absin | abs1850066 |
| Pacific anti-Mouse CD4 | Absin | abs182367 |
| Anti-Mouse CD8α | Invivogen | 3004668 |
| APC Rat anti-Mouse CD25 | Absin | abs182338 |
| PE Rat anti-Mouse Foxp3 | BD Pharmingen^TM^ | 563101 |
| Anti-mouse Granzyme B | Invivogen | 2889689 |
| APC-Cy7 Mouse anti-Mouse NK1.1 | Absin | abs1841010 |
| Pacific Blue Armenian Hamster anti-mouse CD11c | Absin | abs1860009 |
| APC-Cy Rat anti-human/mouse CD11b | Absin | abs1850017 |
| PE Rat anti-mouse F4/80 | Absin | Abs1850177 |
| PE-Cy7 Rat anti-Mouse CD86 | Absin | Abs1850145 |
| RB705 Rat Anti-Mouse Ly6G/Ly-6C | BD Horizon^TM^ | 570633 |
| Alexa Fluor® 647 anti-mouse CD206 | Biolegend | 141712 |
| APC Rat Anti-Mouse I-A/I-E Antibody (S-R511 | STARTER | S0B1677-50T |
| Spark Red^TM^ 718 anti-mouse CD274 | Biolegend | 124348 |

| **Table S2. Sequences of siRNAs used in this study** | |
| --- | --- |
| **Name** | **Sense (5’-3’)** |
| si-PD-L1-1 | TCAATTGTCATATTGCTAC |
| si-PD-L1-2 | TTGACTCCATCTTTCTTCA |
| si-SPOP-1 | CACAAGGCUAUCUUAGCAGCU |
| si-SPOP-2 | CUCCUACAUGUGGACCAUCAA |
| si-MARCH8-1 | GGACATTTCATGAGT CATT |
| si-MARCH8-2 | GGAAGAGACTCAAGGCCTA |
| si-BTRC-1 | GCGUUGUAUUCGAUUUGAUAA |
| si-BTRC-2 | GCUGAACUUGUGUGCAAGGAA |
| si-STUB1-1 | GCAGUCUGUGAAGGCGCACUU |
| si-STUB1-2 | CCCAAGUUCUGCUGUUGGACU |
| si-ARIH1-1 | CGAGAUAUUUCCCAAGAUUUU |
| si-ARIH1-2 | CCAUGUUGUUAAAGUCCAAUA |
| si-HRD1-1 | CCAUGAGGCAGUUCAAGAAdTdT |
| si-HRD1-2 | UGUCUGGCCUUCACCGUUU |

| **Table S3. Sequences of primers used in this study** | | |
| --- | --- | --- |
| **Primer name** | **Forward 5’-3’** | **Reverse 5’-3’** |
| Human PD-L1 | GGCATTTGCTGAACGCAT | CAATTAGTGCAGCCAGGT |
| Human β-actin | ATTCCTATGTGGGCGACGAG | CCAGATTTTCTCCATGTCGTCC |
| Human SPOP | GCCAGTGAAATACGAGTTAGGG | CCTGGAGCGCTTAAAGGTCA |
| Human MARCH8 | AGTGACATTCCACGTCATTGC | GATCTCCTCAGCAGTACGGTC |
| Human BTRC | TGGCTCATCTGACAACACTATC | CGAATACAACGCACCAATTCC |
| Human STUB1 | CGAATACAACGCACCAATTCC | TCAAGGAGCAGGGCAATCGTCT |
| Human ARIH1 | GCATCTTCAGGTAGCACAAGGC | ACTTTGATGGAAACCTGGAGAA |
| Human HRD1 | TGCGTAACATCCACACACTG | CTTTGAGTTTGTATCTTGGATGCC |

**Table S4. Clinicopathologic characteristics of PD-1 mAb therapy in rectal cancer cohorts, related to Figure 9**

| **Patient No.** | **Response** | **Patients** | **Tumor types** | **TNM stage** | **TRG** | **PS score** | **Changes in diameter (%)** |
| --- | --- | --- | --- | --- | --- | --- | --- |
| 1 | PR | Adult patient | AC | T4N2M0 | 1 | 1 | -70 |
| 2 | CR | Adult patient | AC | T3N1M0 | 0 | 0 | -80 |
| 3 | CR | Adult patient | AC | T3N2M0 | 0 | 0 | -100 |
| 4 | PR | Adult patient | AC | T3N1M0 | 1 | 0 | -50 |
| 5 | PR | Adult patient | AC | T3N2M0 | 1 | 1 | -50 |
| 6 | CR | Adult patient | AC | T3N1M0 | 0 | 0 | -70 |
| 7 | SD | Adult patient | AC | T3N2M0 | 2 | 0 | -10 |
| 8 | SD | Adult patient | AC | T4N2M0 | 3 | 1 | -20 |
| 9 | SD | Adult patient | AC | T3N1M0 | 2 | 0 | -10 |
| 10 | PD | Adult patient | AC | T3N1M0 | 3 | 0 | 20 |
| 11 | SD | Adult patient | AC | T3N1M0 | 2 | 0 | -20 |
| 12 | PD | Adult patient | AC | T3N2M0 | 3 | 0 | 10 |
| 13 | SD | Adult patient | AC | T3N2M0 | 2 | 0 | -20 |
| 14 | PD | Adult patient | AC | T3N1M0 | 3 | 1 | 10 |
| 15 | SD | Adult patient | AC | T3N2M0 | 2 | 0 | -20 |

PD-1, programmed death-1; CR, complete response; PR, partial response; SD, stable disease; PD, progressive disease; AC, adenocarcinoma; TRG, tumor regression grade; PS, performance status.

**Table S5. Clinicopathologic characteristics of PD-1 mAb therapy in rectal cancer cohorts, related to Figure 9**

| **Patient Characteristics** | **Responders** | **Non-responders** |
| --- | --- | --- |
|  | (n=6) | (n=9) |
| Age (years) | 53.2±15.7 | 59±12.1 |
| mean±SD |  |  |
| Male | 3 | 4 |
| Female | 3 | 5 |
| Response, n (%) |  |  |
| CR | 3 (50%) | 0 |
| PR | 3 (50%) | 0 |
| SD | 0 | 6 (66.7%) |
| PD | 0 | 3 (33.3%) |

PD-1, programmed death-1; CR, complete response; PR, partial response; SD, stable disease; PD, progressive disease. Patient with CR and PR were classified as responders, while patients with SD and PD were classified as non-responders.

**Table S6. Clinicopathological characteristics of PD-1 mAb therapy in lung cancer, related to Figure 9**

| **Patient No.** | **Response** | **Patients** | **Tumor types** | **TNM stage** | **TRG** | **PS score** | **Changes in diameter (%)** |
| --- | --- | --- | --- | --- | --- | --- | --- |
| 1 | CPR | Adult patient | LUSC | T2aN0M1 | 0 | 0 | -16 |
| 2 | MPR | Adult patient | LUSC | T4N1M0 | 2 | 1 | -42 |
| 3 | MPR | Adult patient | LUSC | T4N2bM0 | 2 | 1 | -17 |
| 4 | CPR | Adult patient | LUSC | T2bN2bM0 | 0 | 1 | -34 |
| 5 | MPR | Adult patient | LUSC | T4N1N0 | 2 | 0 | -56 |
| 6 | CPR | Adult patient | LUSC | T2bN1M0 | 0 | 0 | -68 |
| 7 | CPR | Adult patient | LUSC | T3N1M0 | 0 | 0 | -45 |
| 8 | MPR | Adult patient | LUSC | T3N2bM0 | 2 | 0 | -47 |
| 9 | Non-MPR | Adult patient | LUSC | T4N2aM0 | 3 | 0 | -14 |
| 10 | Non-MPR | Adult patient | LUAD | T2aN2bM0 | 3 | 0 | -14 |
| 11 | Non-MPR | Adult patient | LUAD | T2aN0M0 | 3 | 0 | -6 |
| 12 | Non-MPR | Adult patient | LUSC | T3N2bM0 | 3 | 0 | -6 |
| 13 | Non-MPR | Adult patient | LUSC | T1cN1M0 | 3 | 0 | -7 |
| 14 | Non-MPR | Adult patient | LUAD | T1cN0M0 | 3 | 0 | 14 |
| 15 | Non-MPR | Adult patient | LUAD | T3N2bM0 | 3 | 0 | -7 |
| 16 | Non-MPR | Adult patient | LUSC | T3N2bM0 | 3 | 1 | -8 |

PD-1, programmed death-1; CPR, Complete Pathologic Response; MPR, Major Pathologic Response; Non-MPR, Non-Major Pathologic Response; LUAD, Lung adenocarcinoma; LUSC, lung squamous-cell carcinoma; TRG, tumor regression grade; PS, performance status.

**Table S7. Clinicopathological characteristics of PD-1 mAb therapy in lung cancer, related to Figure 9**

| **Patient Characteristics** | **Responders** | **Non-responders** |
| --- | --- | --- |
|  | (n=8) | (n=8) |
| Age (years) | 58.8±7.479 | 58.3±6.182 |
| mean±SD |  |  |
| Male | 7 | 6 |
| Female | 1 | 2 |
| Response, n (%) |  |  |
| CPR | 4 (25%) | 0 |
| MPR | 4 (25%) | 0 |
| Non-MPR | 0 | 8 (50%) |

PD-1, programmed death-1; CPR, Complete Pathologic Response; MPR, Major Pathologic Response; Non-MPR, Non-Major Pathologic Response. Patient with CPR and MPR were classified as responders, while patients with Non-MPR was classified as non-responders.
