# Supplementary material for: Paroxetine repurposing enhances antitumor immunity via SPOP-mediated PD-L1 ubiquitination and proteasomal degradation
Source: J Exp Clin Cancer Res. 2026 Jan 27;45:57. doi: 10.1186/s13046-026-03648-z (PMC12918487; doi:10.1186/s13046-026-03648-z)
Supplement: Supplementary file 2 — Supplementary Material 2. [file 13046_2026_3648_MOESM2_ESM.pptx]

## Slide 1
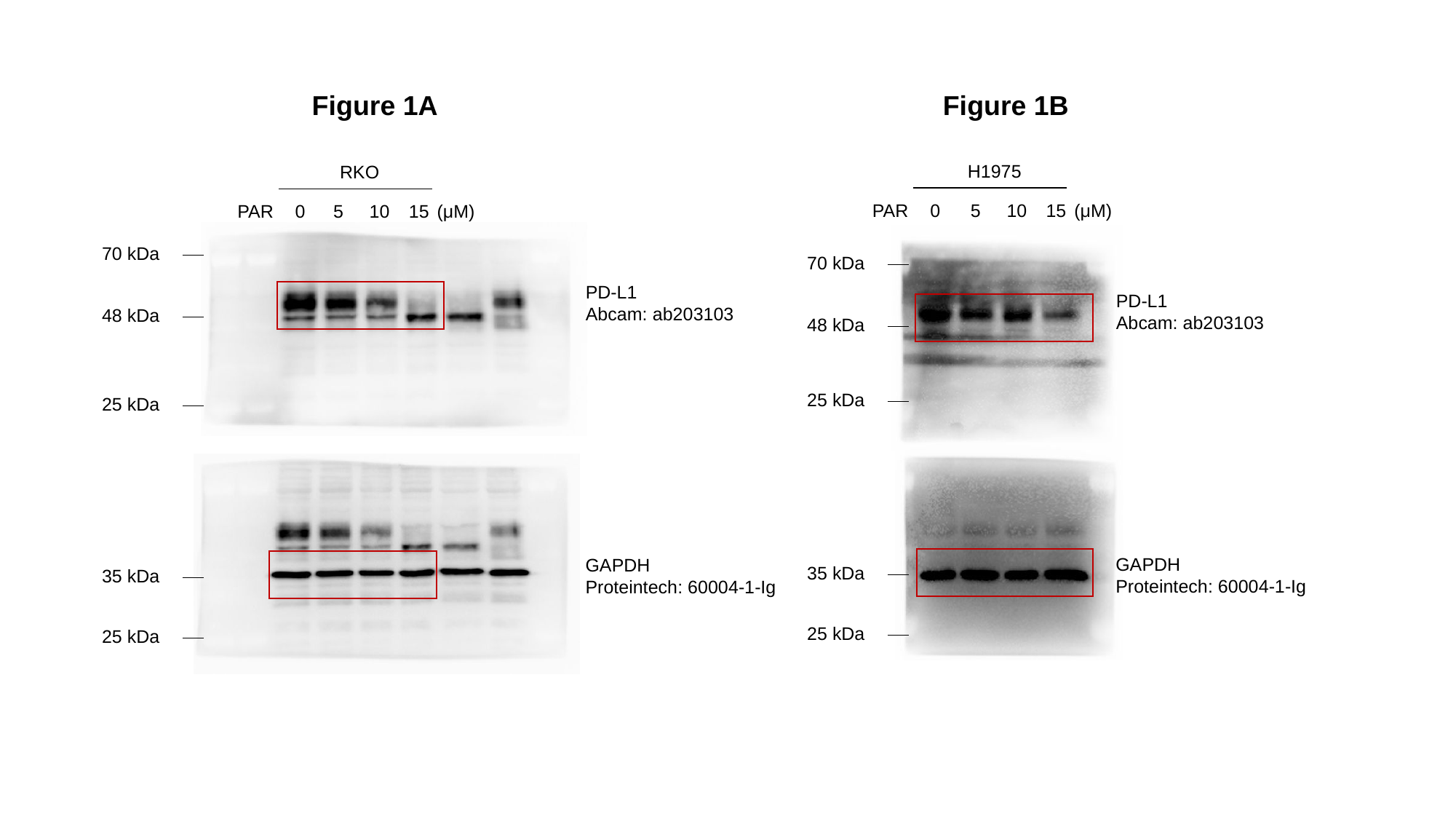

Figure 1A
Figure 1B
H1975
RKO
PAR
(μM)
0
5
10
15
PAR
(μM)
0
5
10
15
70 kDa
70 kDa
PD-L1
Abcam: ab203103
PD-L1
Abcam: ab203103
48 kDa
48 kDa
25 kDa
25 kDa
GAPDH
Proteintech: 60004-1-Ig
GAPDH
Proteintech: 60004-1-Ig
35 kDa
35 kDa
25 kDa
25 kDa

## Slide 2
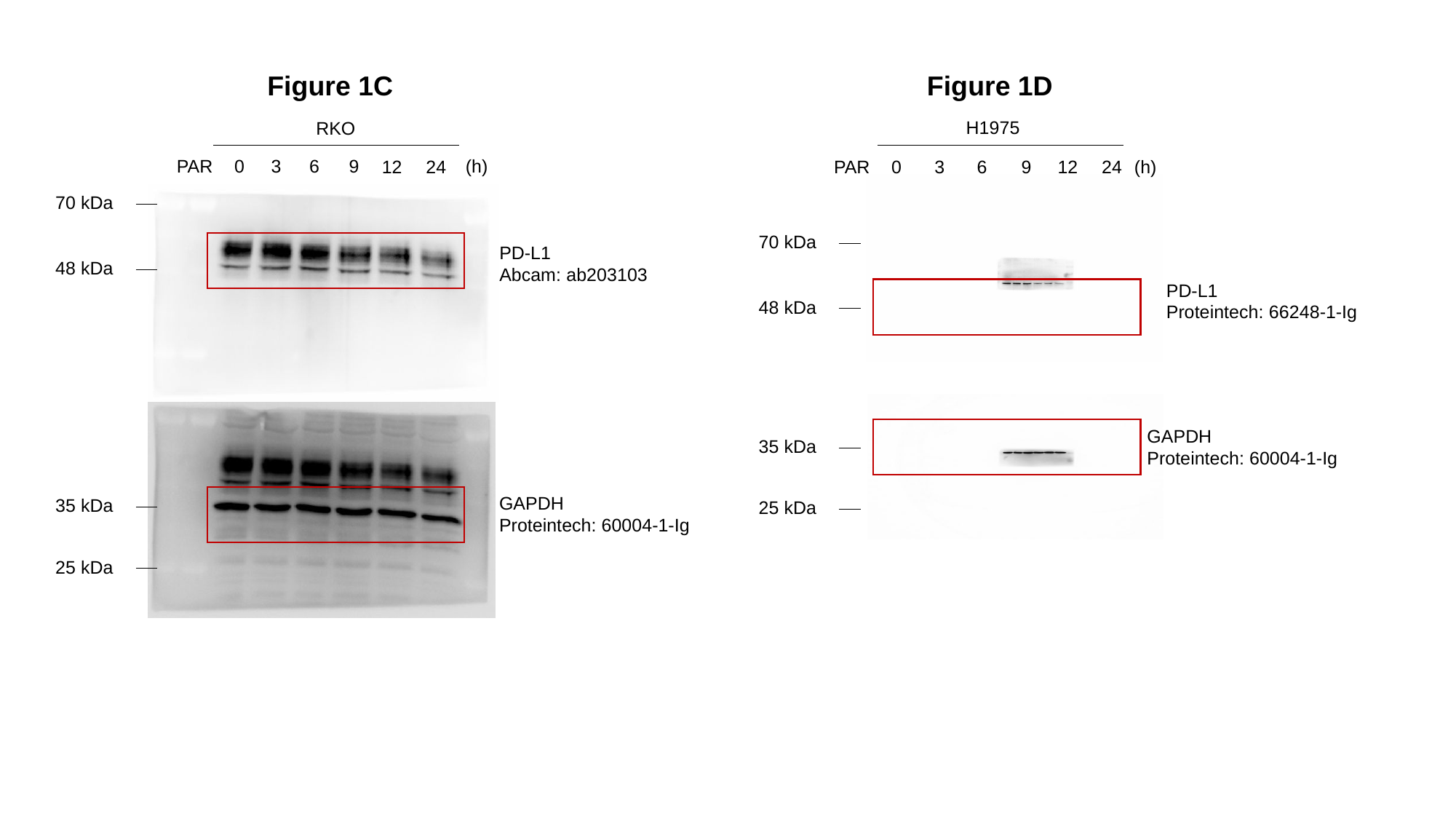

Figure 1C
Figure 1D
H1975
RKO
PAR
(h)
0
3
6
9
PAR
(h)
0
3
6
9
12
24
12
24
70 kDa
70 kDa
PD-L1
Abcam: ab203103
48 kDa
PD-L1
Proteintech: 66248-1-Ig
48 kDa
GAPDH
Proteintech: 60004-1-Ig
35 kDa
GAPDH
Proteintech: 60004-1-Ig
35 kDa
25 kDa
25 kDa

## Slide 3
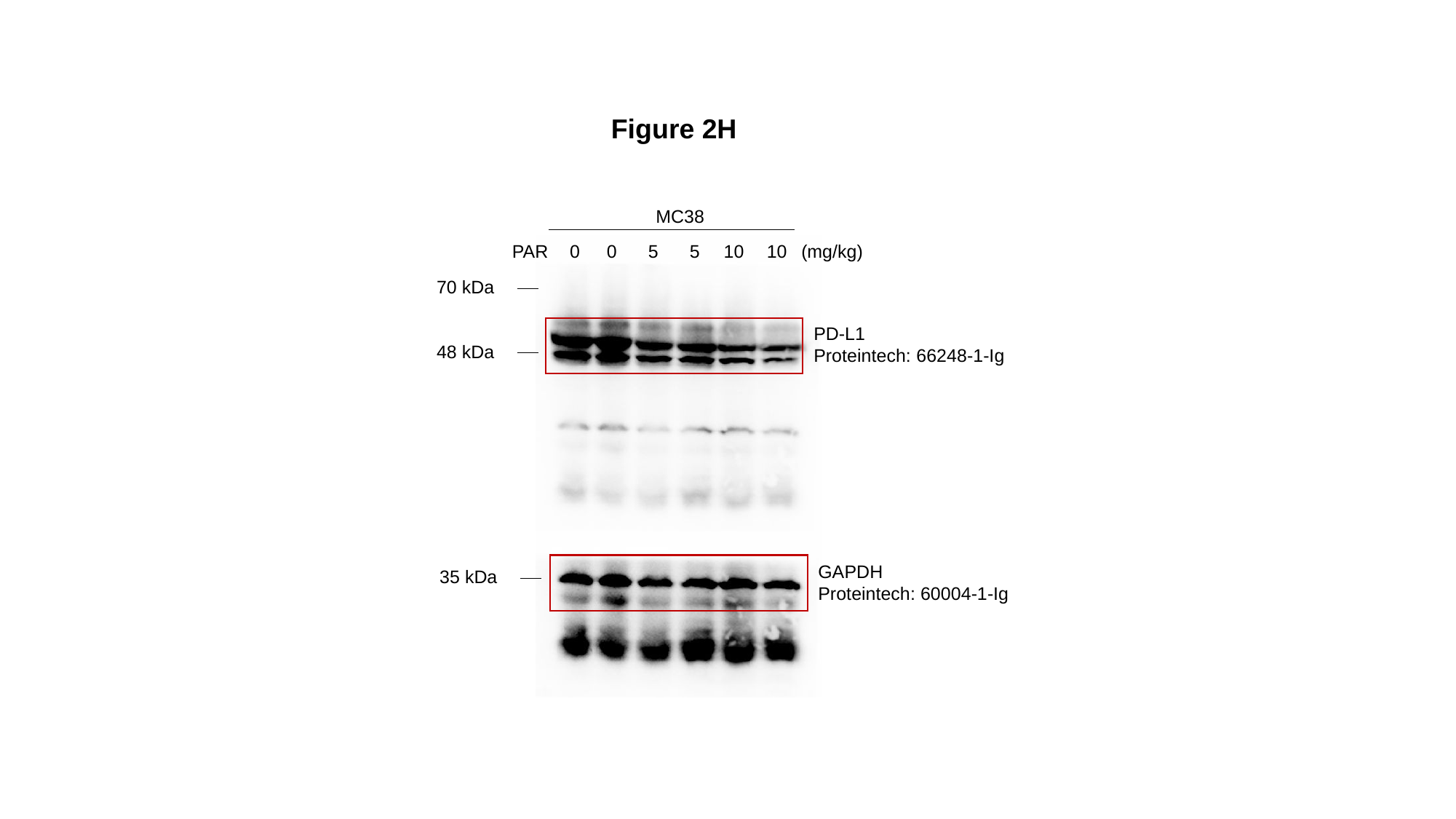

Figure 2H
MC38
PAR
(mg/kg)
0
0
5
5
10
10
70 kDa
PD-L1
Proteintech: 66248-1-Ig
48 kDa
GAPDH
Proteintech: 60004-1-Ig
35 kDa

## Slide 4
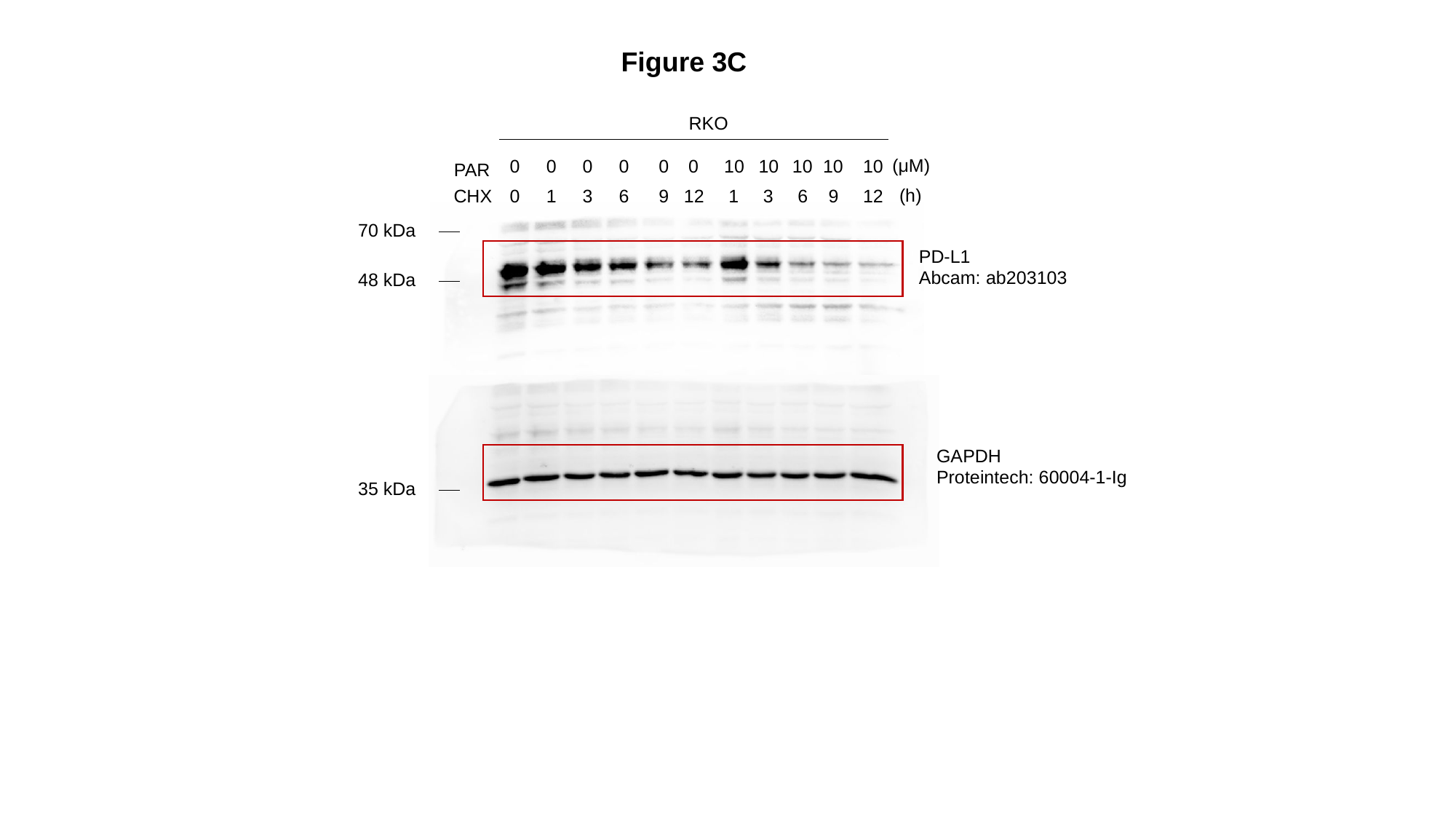

Figure 3C
RKO
(μM)
0
0
0
0
0
0
10
10
10
10
10
PAR
(h)
CHX
0
1
3
6
9
12
1
3
6
9
12
70 kDa
PD-L1
Abcam: ab203103
48 kDa
GAPDH
Proteintech: 60004-1-Ig
35 kDa

## Slide 5
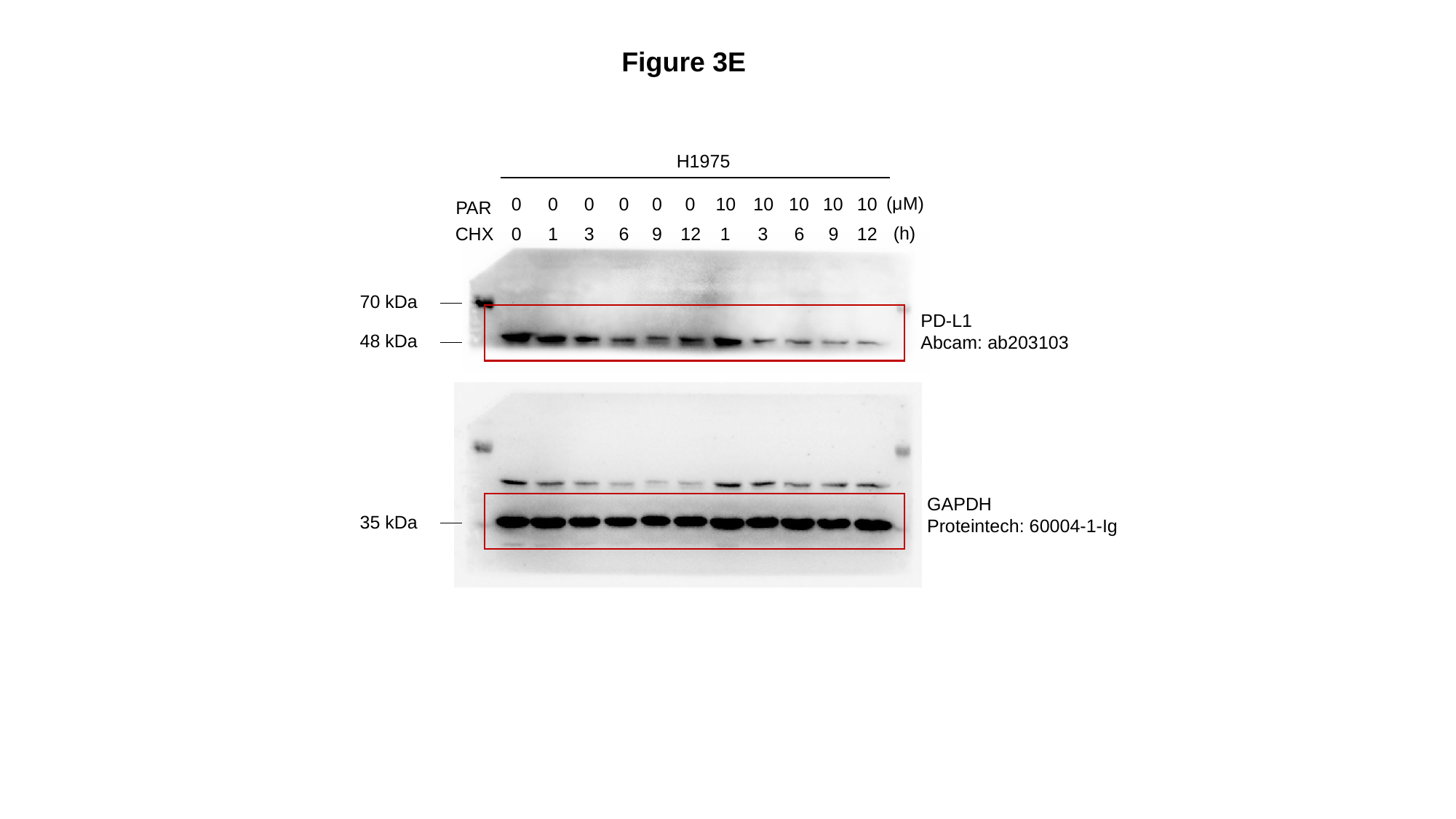

Figure 3E
H1975
(μM)
0
0
0
0
0
0
10
10
10
10
10
PAR
(h)
CHX
0
1
3
6
9
12
1
3
6
9
12
70 kDa
PD-L1
Abcam: ab203103
48 kDa
GAPDH
Proteintech: 60004-1-Ig
35 kDa

## Slide 6
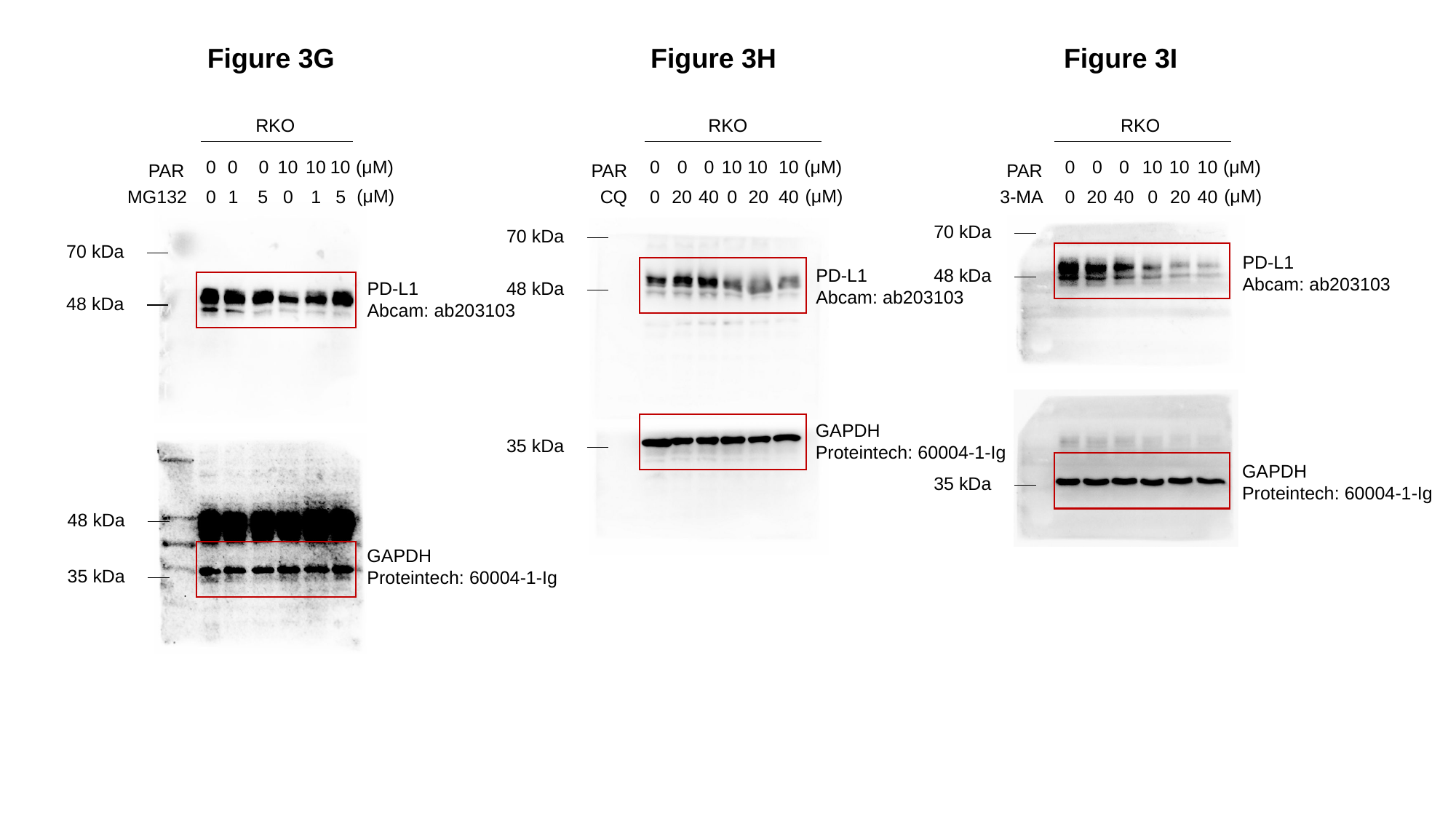

Figure 3G
Figure 3H
Figure 3I
RKO
RKO
RKO
0
0
0
10
10
10
(μM)
0
0
0
10
10
10
(μM)
0
0
0
10
10
10
(μM)
PAR
PAR
PAR
(μM)
(μM)
(μM)
MG132
CQ
0
1
5
0
1
5
0
20
40
0
20
40
3-MA
0
20
40
0
20
40
70 kDa
70 kDa
70 kDa
PD-L1
Abcam: ab203103
PD-L1
Abcam: ab203103
48 kDa
PD-L1
Abcam: ab203103
48 kDa
48 kDa
GAPDH
Proteintech: 60004-1-Ig
35 kDa
GAPDH
Proteintech: 60004-1-Ig
35 kDa
48 kDa
GAPDH
Proteintech: 60004-1-Ig
35 kDa

## Slide 7
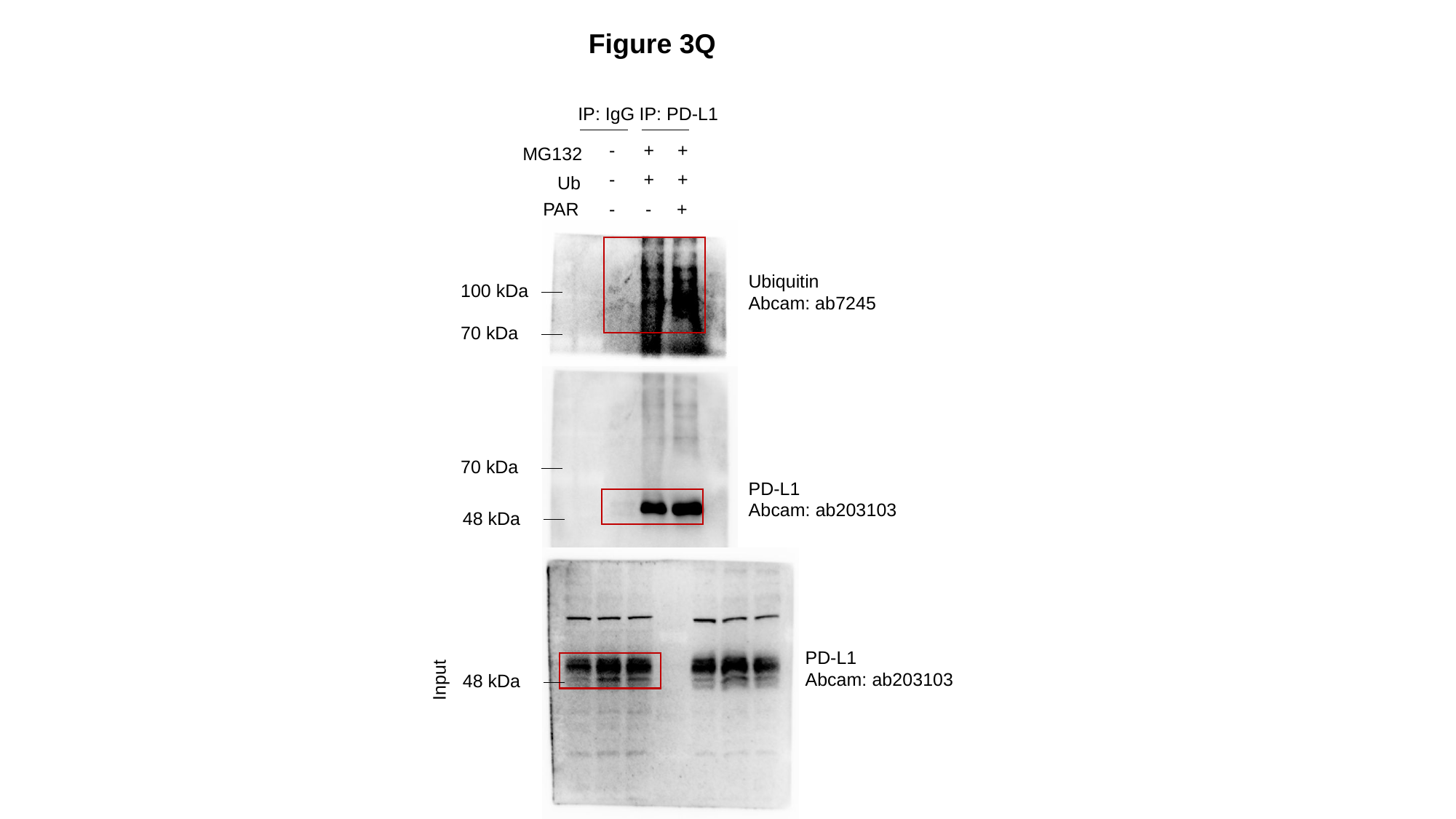

Figure 3Q
IP: IgG
IP: PD-L1
-
+
+
MG132
-
+
+
Ub
PAR
-
-
+
Ubiquitin
Abcam: ab7245
100 kDa
70 kDa
70 kDa
PD-L1
Abcam: ab203103
48 kDa
PD-L1
Abcam: ab203103
Input
48 kDa

## Slide 8
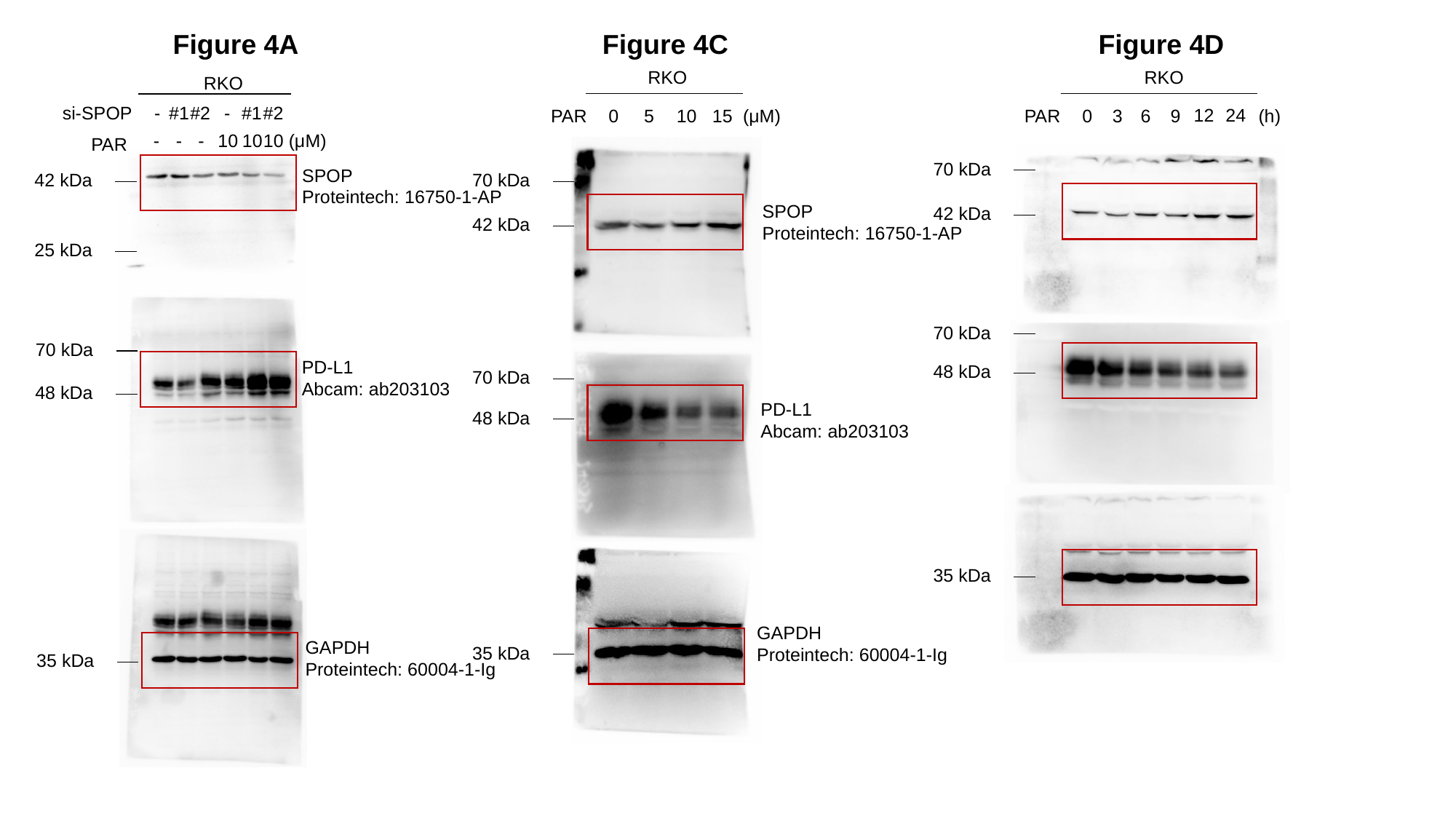

Figure 4A
Figure 4C
Figure 4D
RKO
RKO
RKO
si-SPOP
-
#1
#2
-
#1
#2
12
24
PAR
(μM)
0
5
10
15
PAR
(h)
0
3
6
9
-
-
-
10
10
10
(μM)
PAR
70 kDa
SPOP
Proteintech: 16750-1-AP
70 kDa
42 kDa
SPOP
Proteintech: 16750-1-AP
42 kDa
42 kDa
25 kDa
70 kDa
70 kDa
PD-L1
Abcam: ab203103
48 kDa
70 kDa
48 kDa
PD-L1
Abcam: ab203103
48 kDa
35 kDa
GAPDH
Proteintech: 60004-1-Ig
GAPDH
Proteintech: 60004-1-Ig
35 kDa
35 kDa

## Slide 9
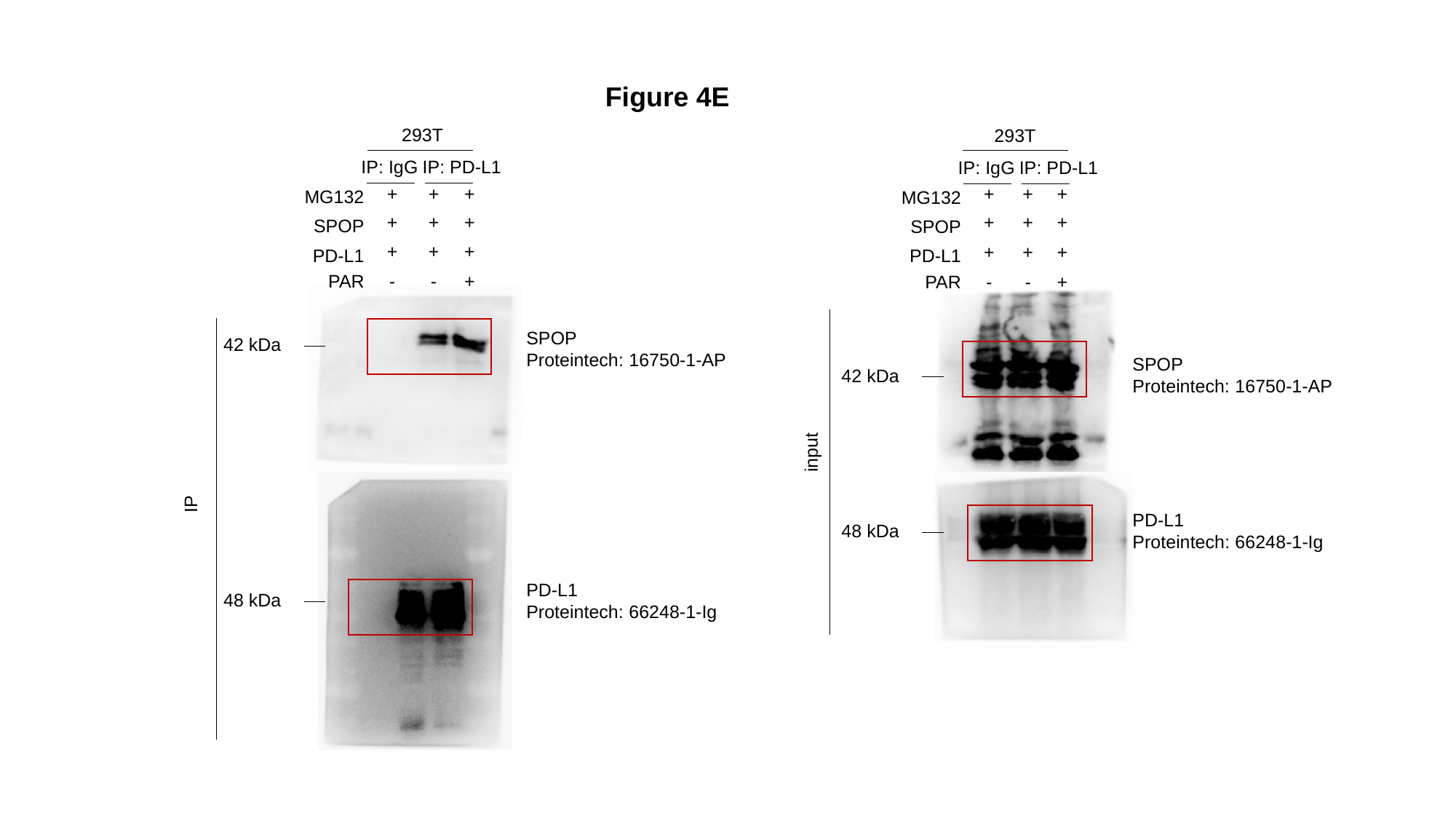

Figure 4E
293T
293T
IP: IgG
IP: PD-L1
IP: IgG
IP: PD-L1
+
+
+
+
+
+
MG132
MG132
+
+
+
+
+
+
SPOP
SPOP
+
+
+
+
+
+
PD-L1
PD-L1
PAR
-
-
+
PAR
-
-
+
SPOP
Proteintech: 16750-1-AP
42 kDa
SPOP
Proteintech: 16750-1-AP
42 kDa
input
IP
PD-L1
Proteintech: 66248-1-Ig
48 kDa
PD-L1
Proteintech: 66248-1-Ig
48 kDa

## Slide 10
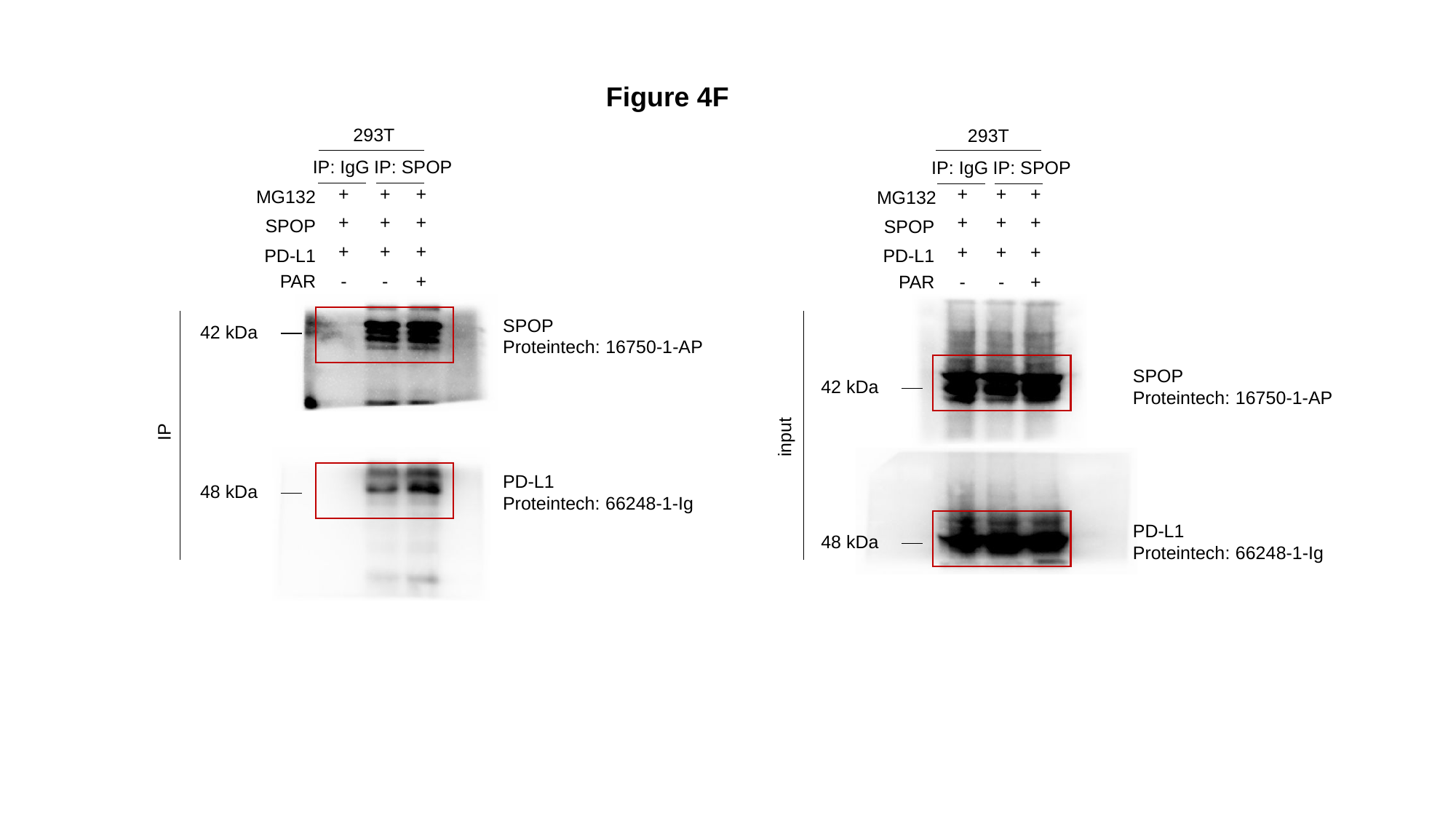

Figure 4F
293T
293T
IP: IgG
IP: SPOP
IP: IgG
IP: SPOP
+
+
+
+
+
+
MG132
MG132
+
+
+
+
+
+
SPOP
SPOP
+
+
+
+
+
+
PD-L1
PD-L1
PAR
-
-
+
PAR
-
-
+
SPOP
Proteintech: 16750-1-AP
42 kDa
SPOP
Proteintech: 16750-1-AP
42 kDa
IP
input
PD-L1
Proteintech: 66248-1-Ig
48 kDa
PD-L1
Proteintech: 66248-1-Ig
48 kDa

## Slide 11
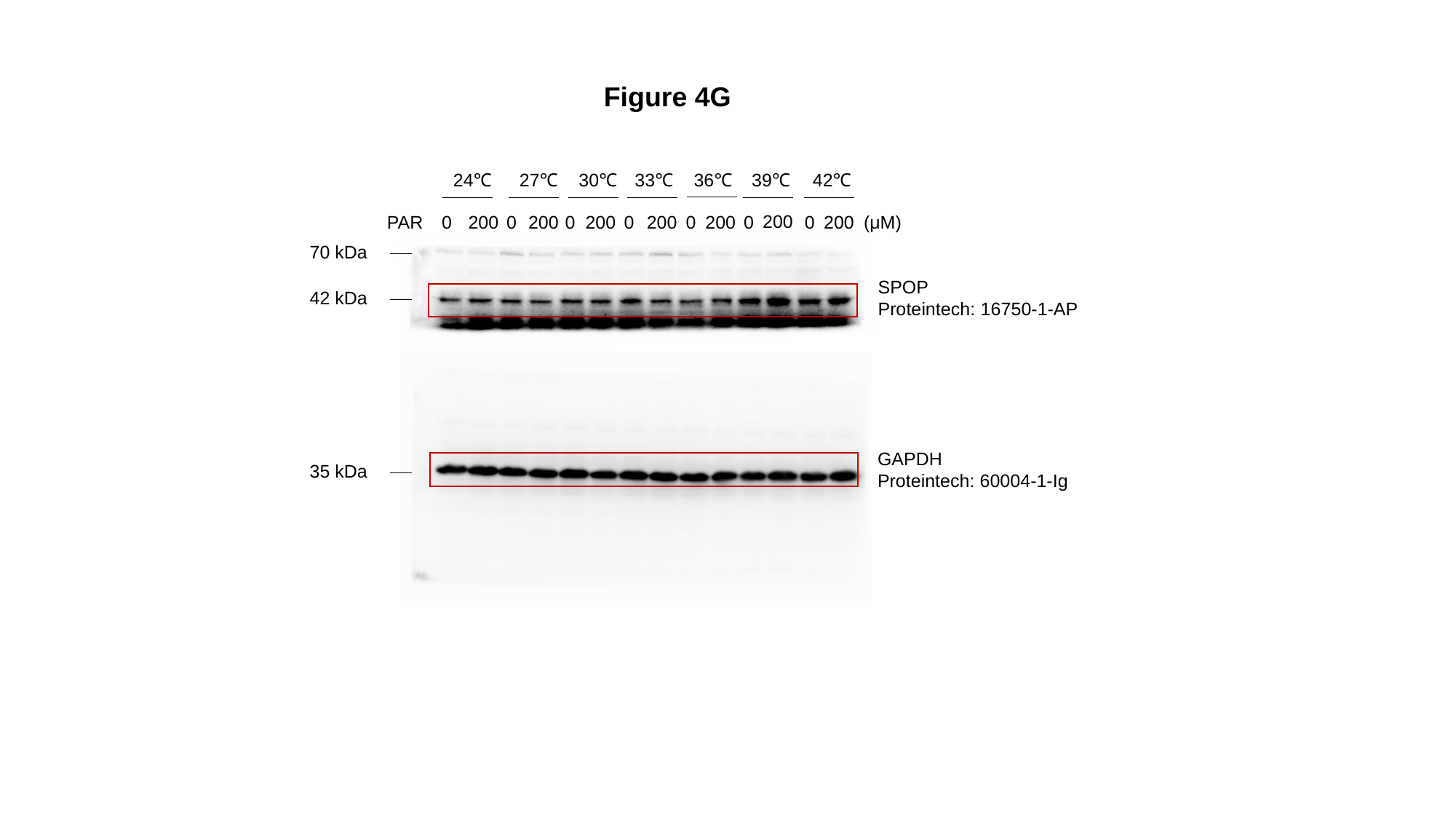

Figure 4G
36℃
39℃
24℃
27℃
30℃
33℃
42℃
200
200
PAR
0
200
0
200
0
200
0
200
0
200
0
0
(μM)
70 kDa
SPOP
Proteintech: 16750-1-AP
42 kDa
GAPDH
Proteintech: 60004-1-Ig
35 kDa

## Slide 12
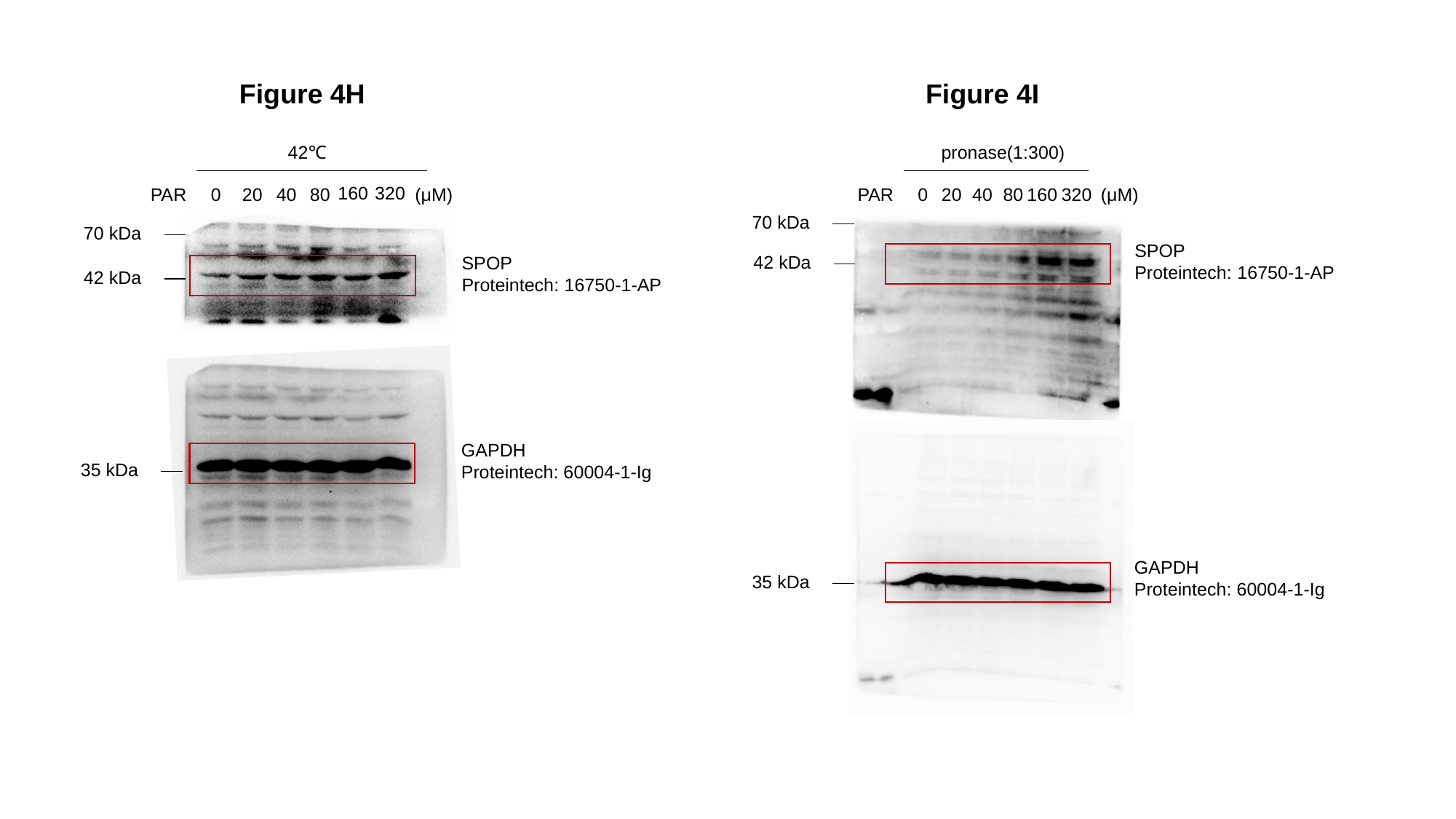

Figure 4H
Figure 4I
42℃
pronase(1:300)
160
320
PAR
PAR
(μM)
0
20
40
80
0
20
40
80
160
320
(μM)
70 kDa
70 kDa
SPOP
Proteintech: 16750-1-AP
42 kDa
SPOP
Proteintech: 16750-1-AP
42 kDa
GAPDH
Proteintech: 60004-1-Ig
35 kDa
GAPDH
Proteintech: 60004-1-Ig
35 kDa

## Slide 13
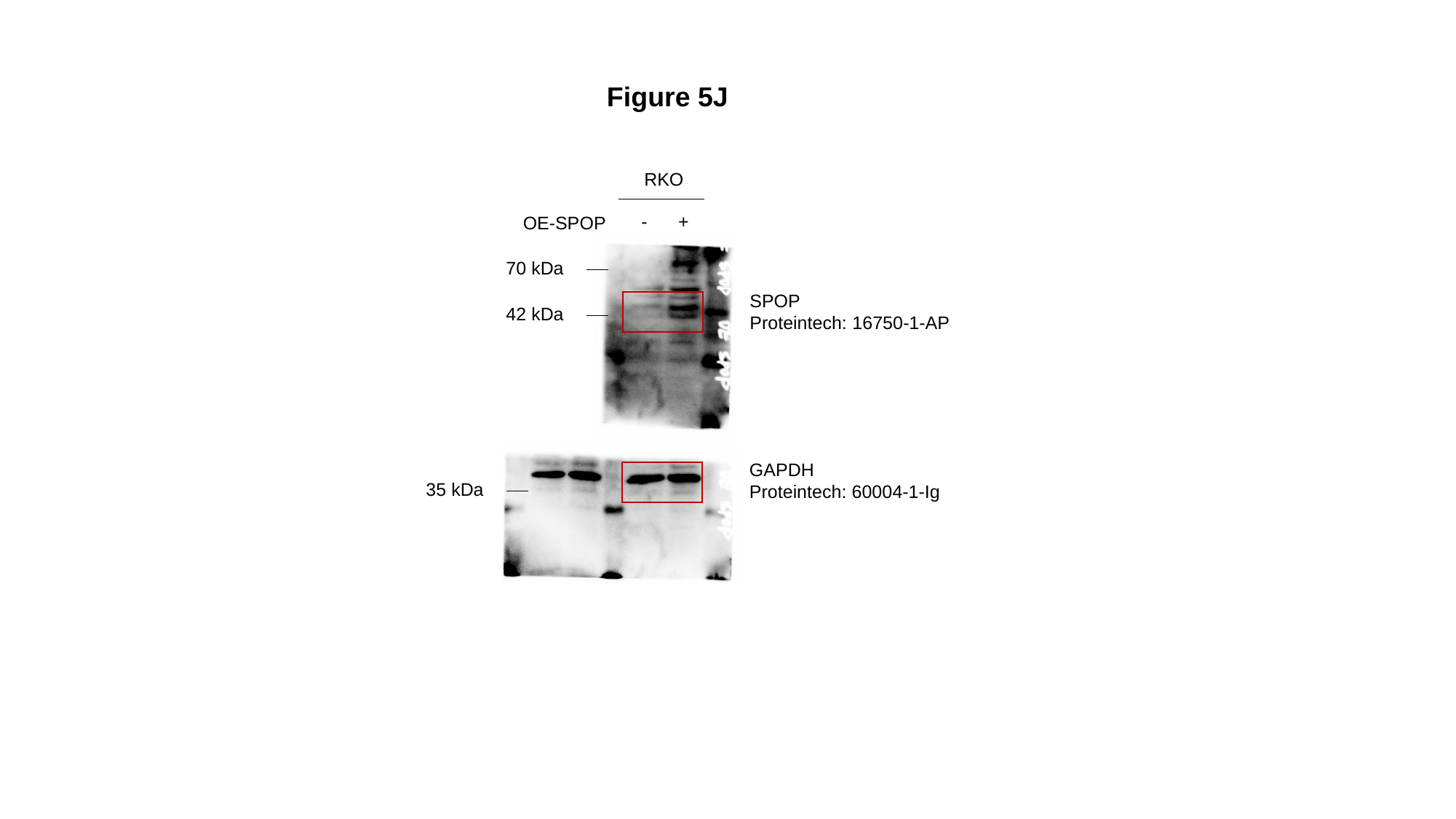

Figure 5J
RKO
-
+
OE-SPOP
70 kDa
SPOP
Proteintech: 16750-1-AP
42 kDa
GAPDH
Proteintech: 60004-1-Ig
35 kDa

## Slide 14
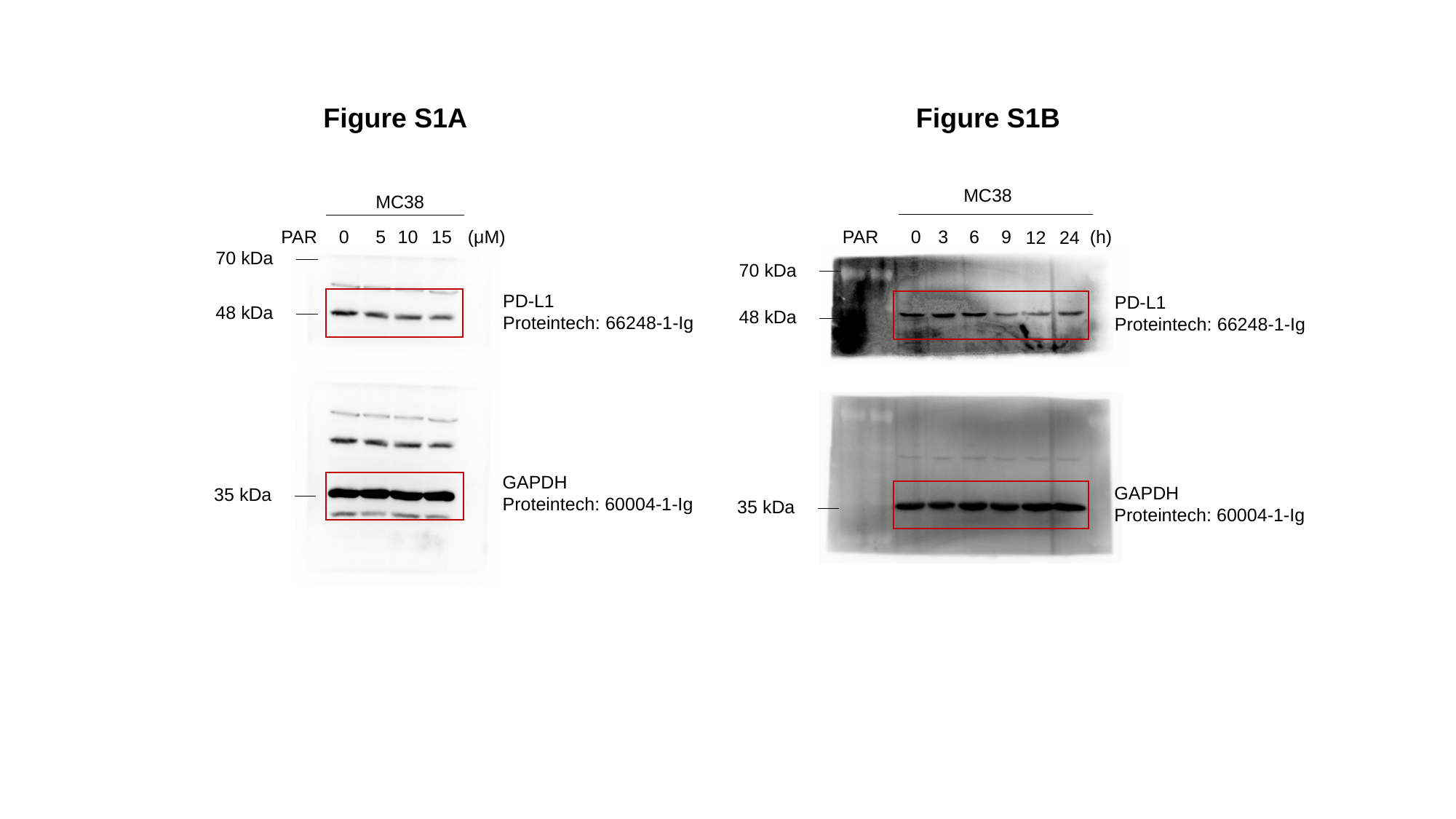

Figure S1A
Figure S1B
MC38
MC38
PAR
(μM)
0
5
10
15
PAR
(h)
0
3
6
9
12
24
70 kDa
70 kDa
PD-L1
Proteintech: 66248-1-Ig
PD-L1
Proteintech: 66248-1-Ig
48 kDa
48 kDa
GAPDH
Proteintech: 60004-1-Ig
GAPDH
Proteintech: 60004-1-Ig
35 kDa
35 kDa

## Slide 15
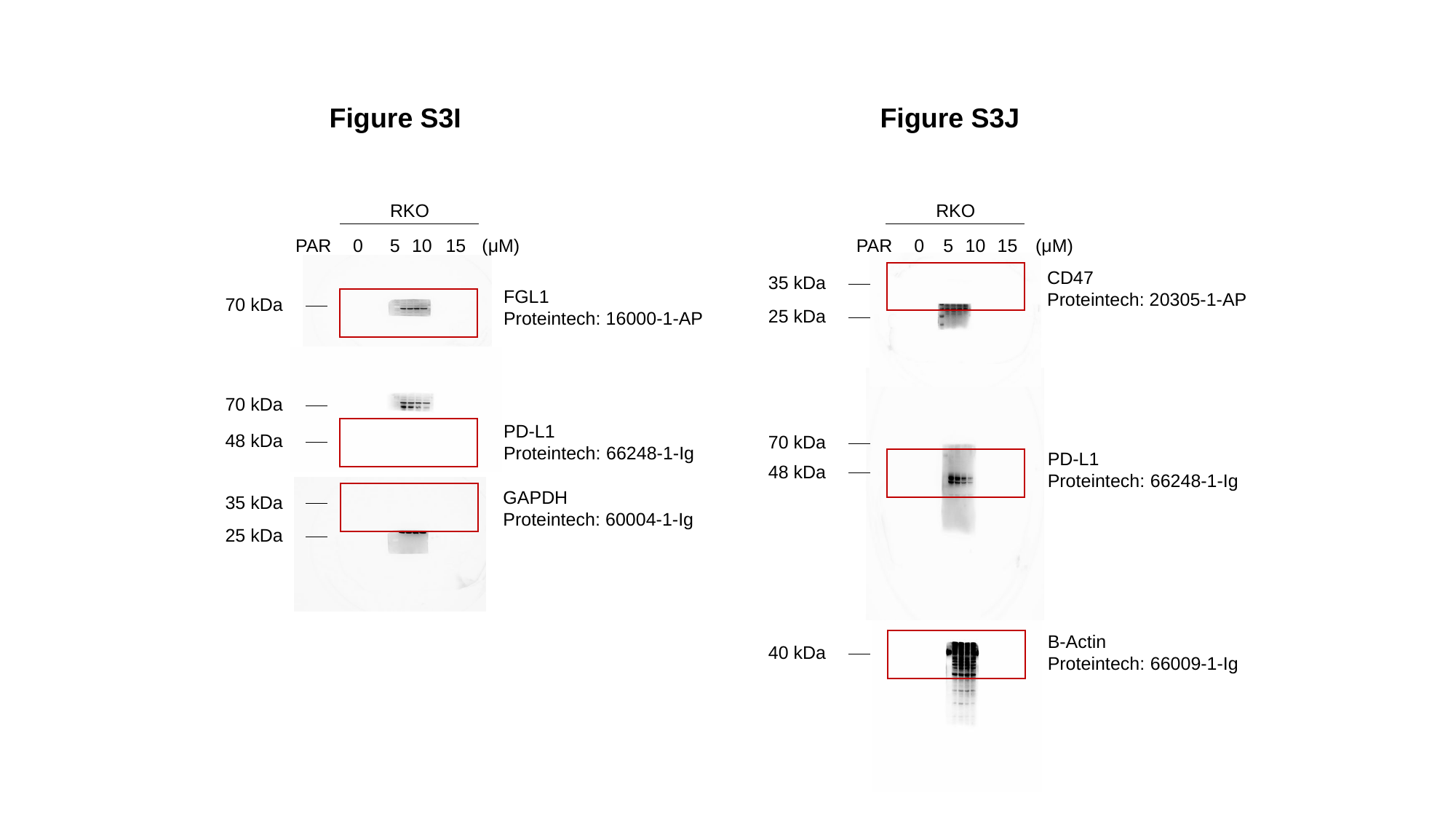

Figure S3I
Figure S3J
RKO
RKO
PAR
PAR
(μM)
(μM)
0
5
10
15
0
5
10
15
CD47
Proteintech: 20305-1-AP
35 kDa
FGL1
Proteintech: 16000-1-AP
70 kDa
25 kDa
70 kDa
PD-L1
Proteintech: 66248-1-Ig
48 kDa
70 kDa
PD-L1
Proteintech: 66248-1-Ig
48 kDa
GAPDH
Proteintech: 60004-1-Ig
35 kDa
25 kDa
Β-Actin
Proteintech: 66009-1-Ig
40 kDa

## Slide 16
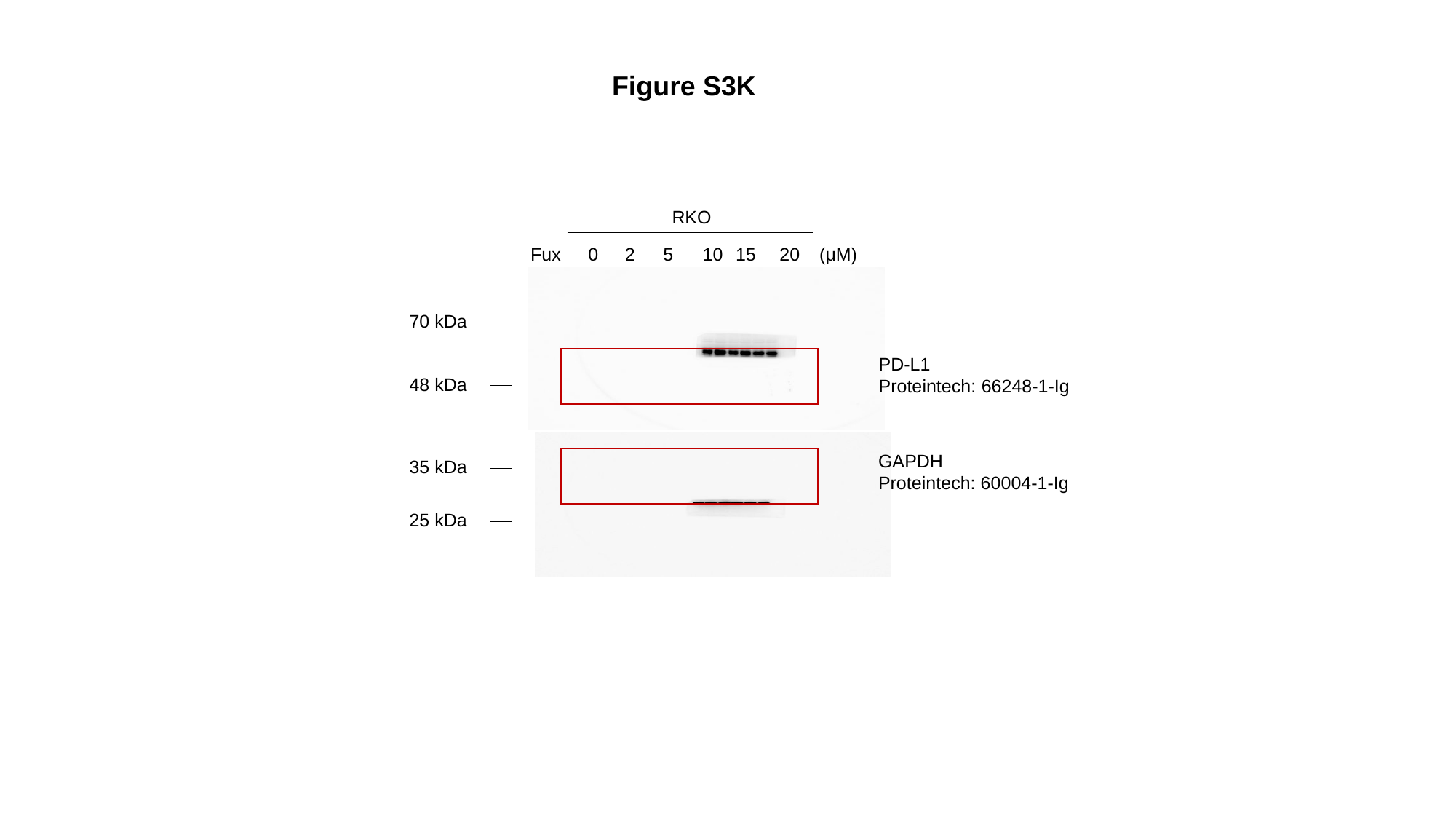

Figure S3K
RKO
Fux
(μM)
0
2
5
10
15
20
70 kDa
PD-L1
Proteintech: 66248-1-Ig
48 kDa
GAPDH
Proteintech: 60004-1-Ig
35 kDa
25 kDa

## Slide 17
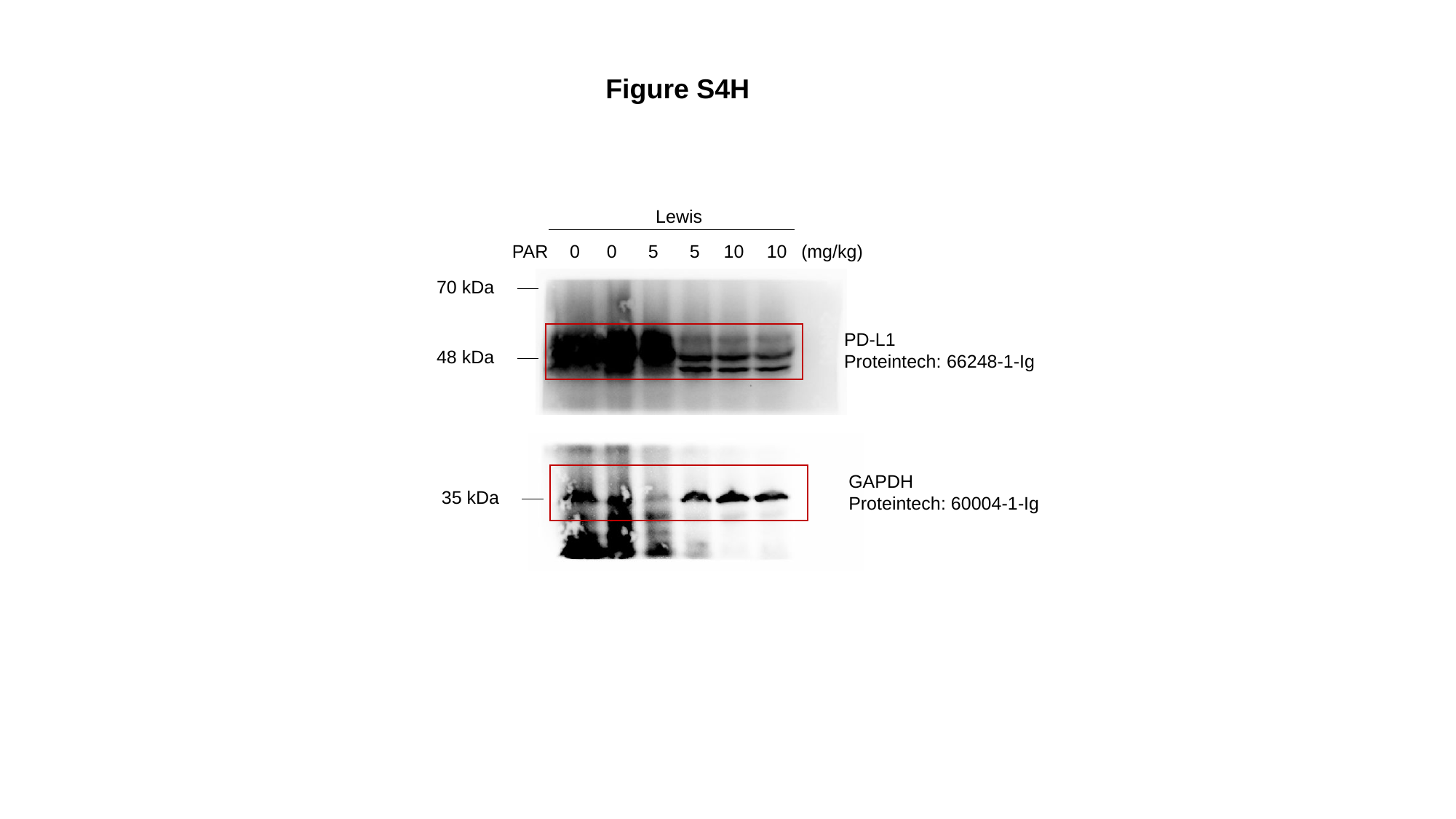

Figure S4H
Lewis
PAR
(mg/kg)
0
0
5
5
10
10
70 kDa
PD-L1
Proteintech: 66248-1-Ig
48 kDa
GAPDH
Proteintech: 60004-1-Ig
35 kDa

## Slide 18
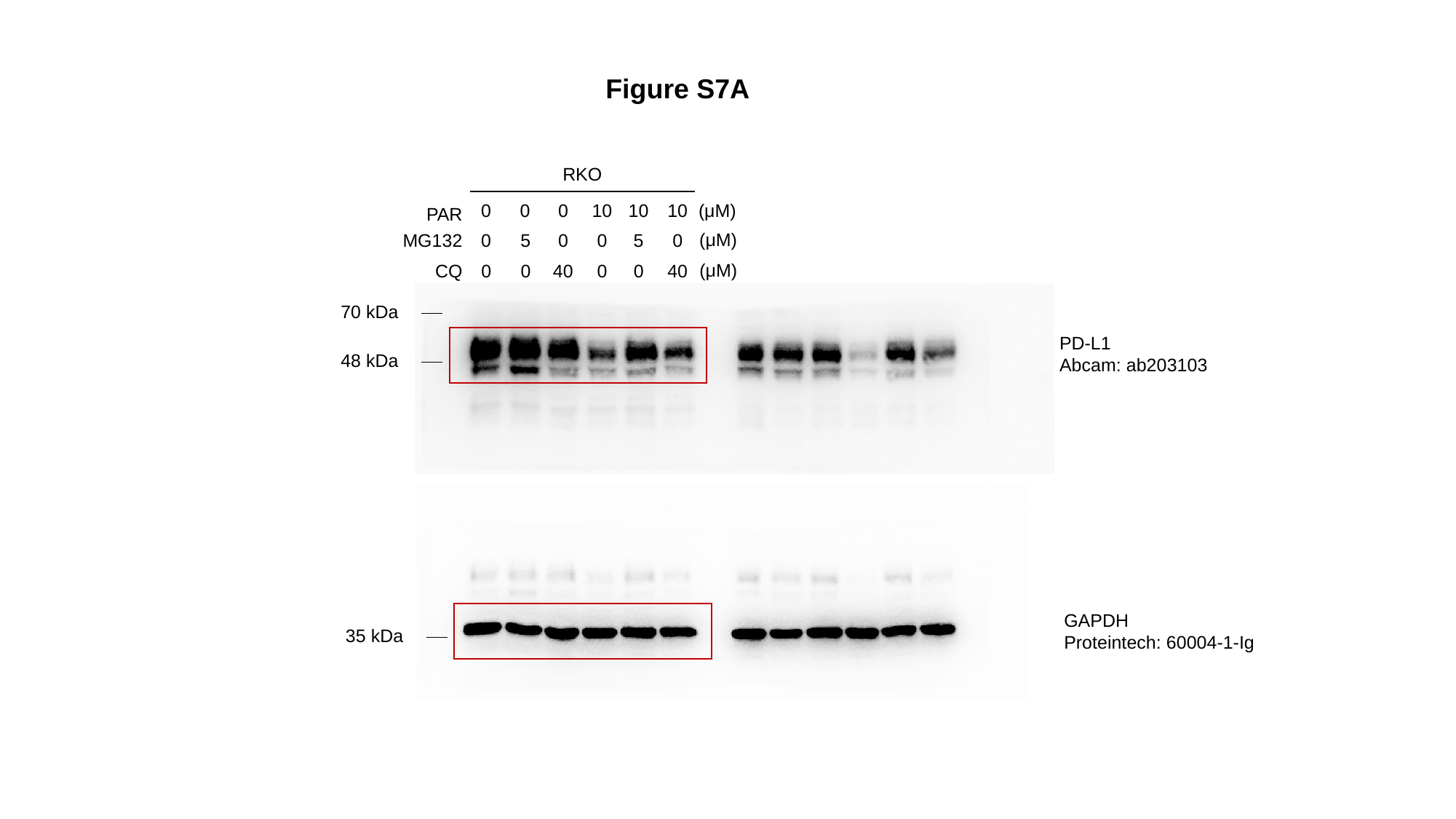

Figure S7A
RKO
0
0
0
10
10
10
(μM)
PAR
(μM)
MG132
0
5
0
0
5
0
(μM)
CQ
0
0
40
0
0
40
70 kDa
PD-L1
Abcam: ab203103
48 kDa
GAPDH
Proteintech: 60004-1-Ig
35 kDa

## Slide 19
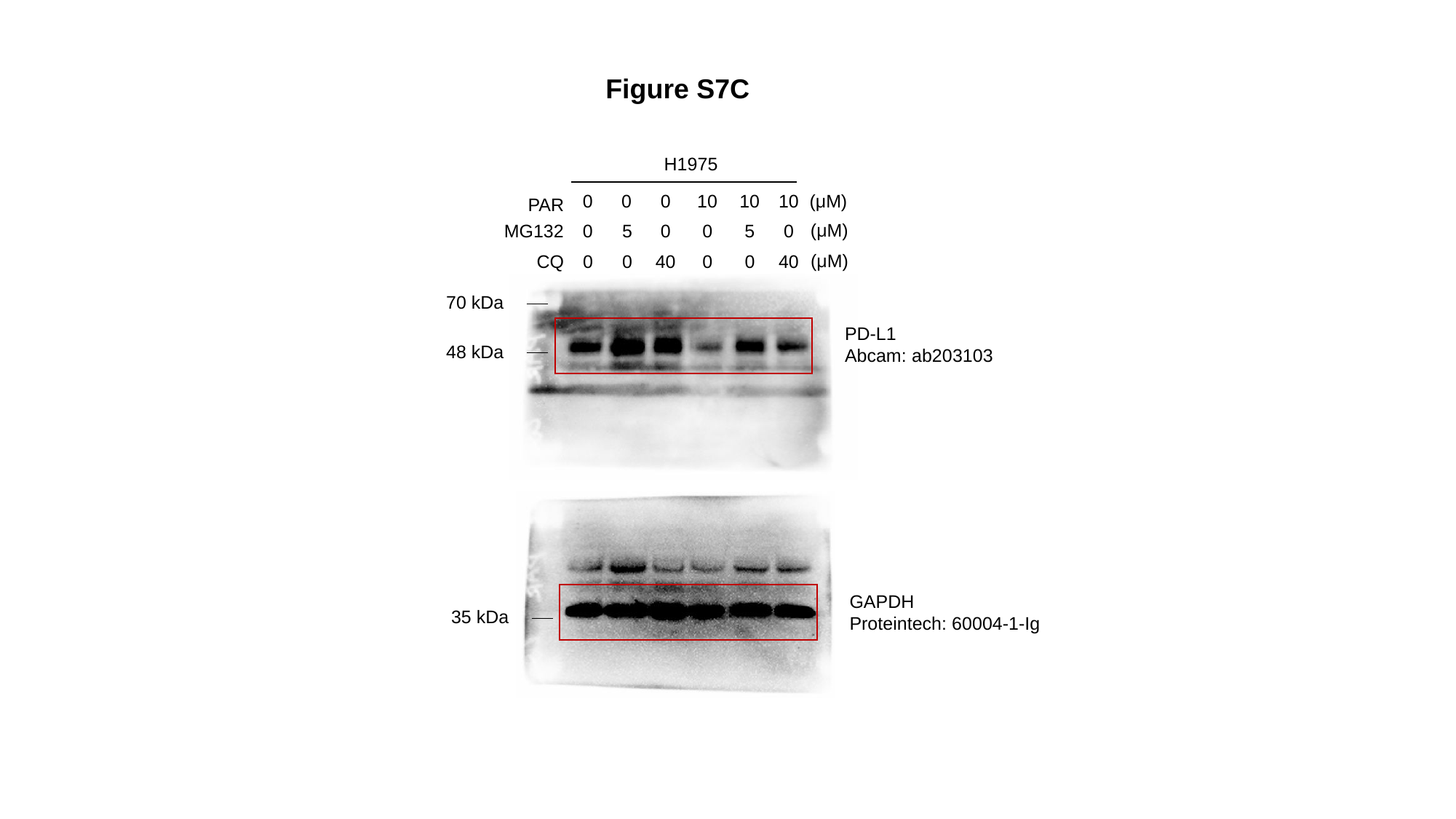

Figure S7C
H1975
0
0
0
10
10
10
(μM)
PAR
(μM)
MG132
0
5
0
0
5
0
(μM)
CQ
0
0
40
0
0
40
70 kDa
PD-L1
Abcam: ab203103
48 kDa
GAPDH
Proteintech: 60004-1-Ig
35 kDa

## Slide 20
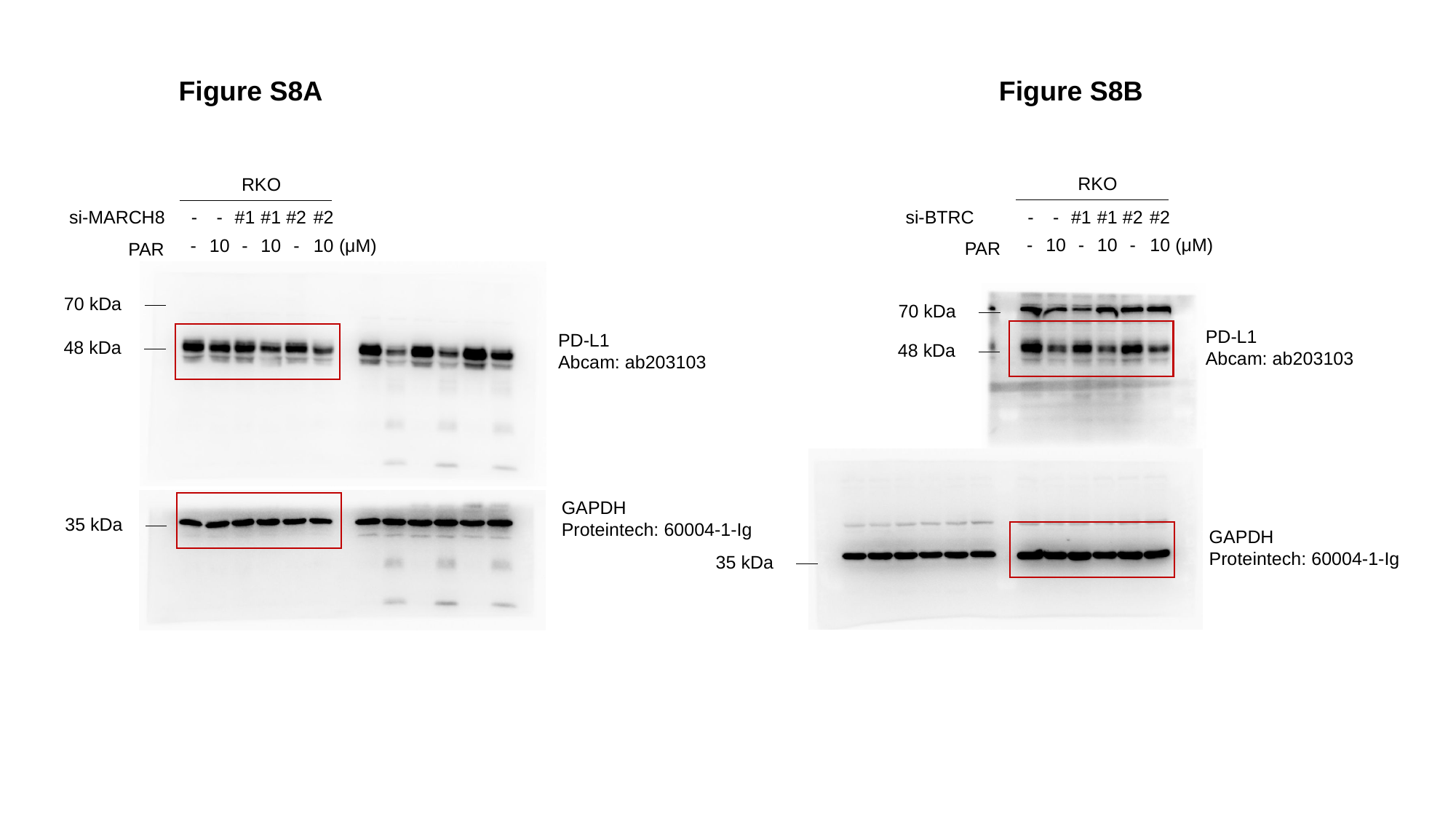

Figure S8A
Figure S8B
RKO
RKO
si-BTRC
-
-
#1
#1
#2
#2
si-MARCH8
-
-
#1
#1
#2
#2
-
10
-
10
-
10
(μM)
-
10
-
10
-
10
(μM)
PAR
PAR
70 kDa
70 kDa
PD-L1
Abcam: ab203103
PD-L1
Abcam: ab203103
48 kDa
48 kDa
GAPDH
Proteintech: 60004-1-Ig
35 kDa
GAPDH
Proteintech: 60004-1-Ig
35 kDa

## Slide 21
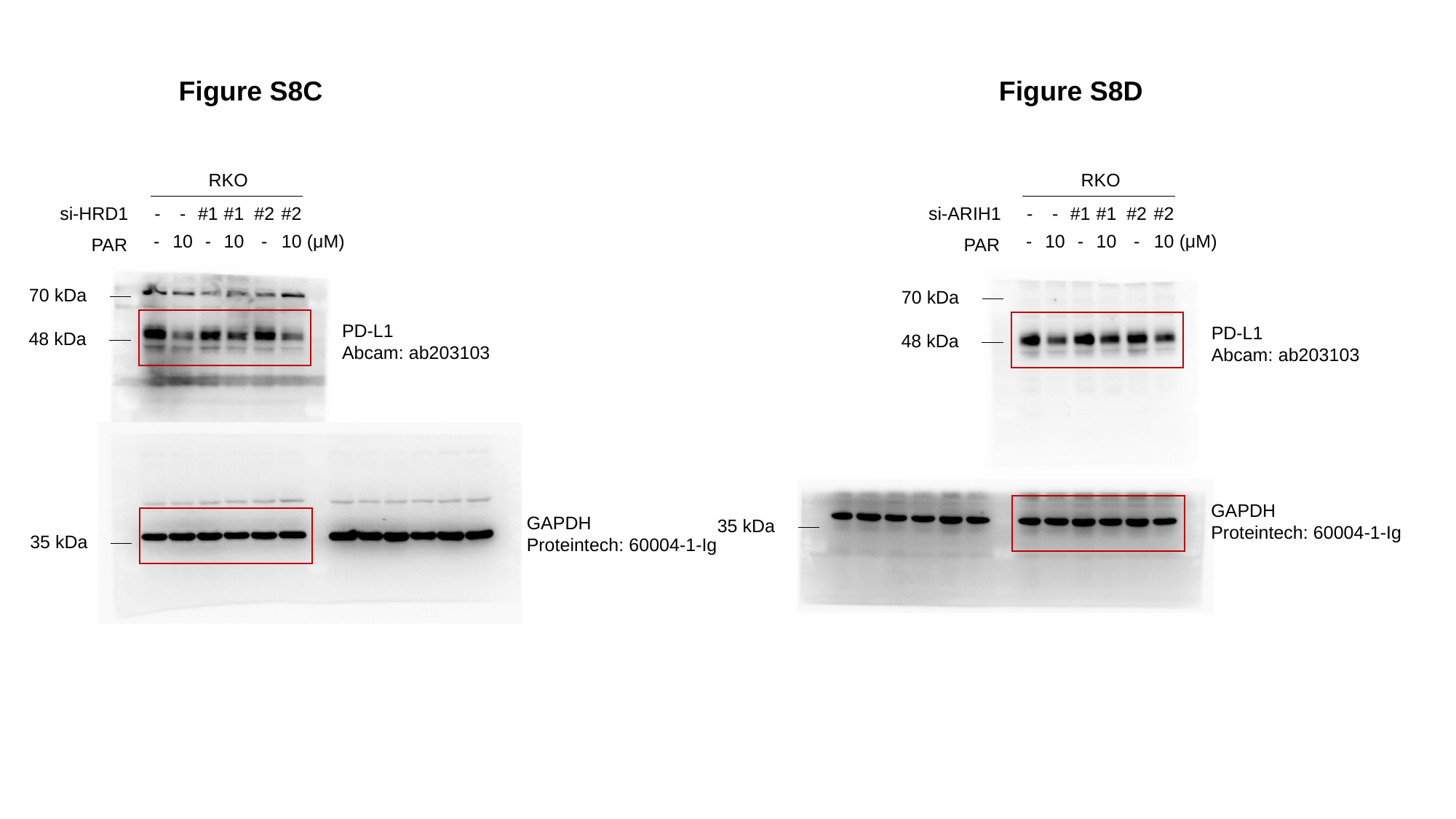

Figure S8C
Figure S8D
RKO
si-HRD1
-
-
#1
#1
#2
#2
-
10
-
10
-
10
(μM)
PAR
RKO
si-ARIH1
-
-
#1
#1
#2
#2
-
10
-
10
-
10
(μM)
PAR
70 kDa
70 kDa
PD-L1
Abcam: ab203103
PD-L1
Abcam: ab203103
48 kDa
48 kDa
GAPDH
Proteintech: 60004-1-Ig
GAPDH
Proteintech: 60004-1-Ig
35 kDa
35 kDa

## Slide 22
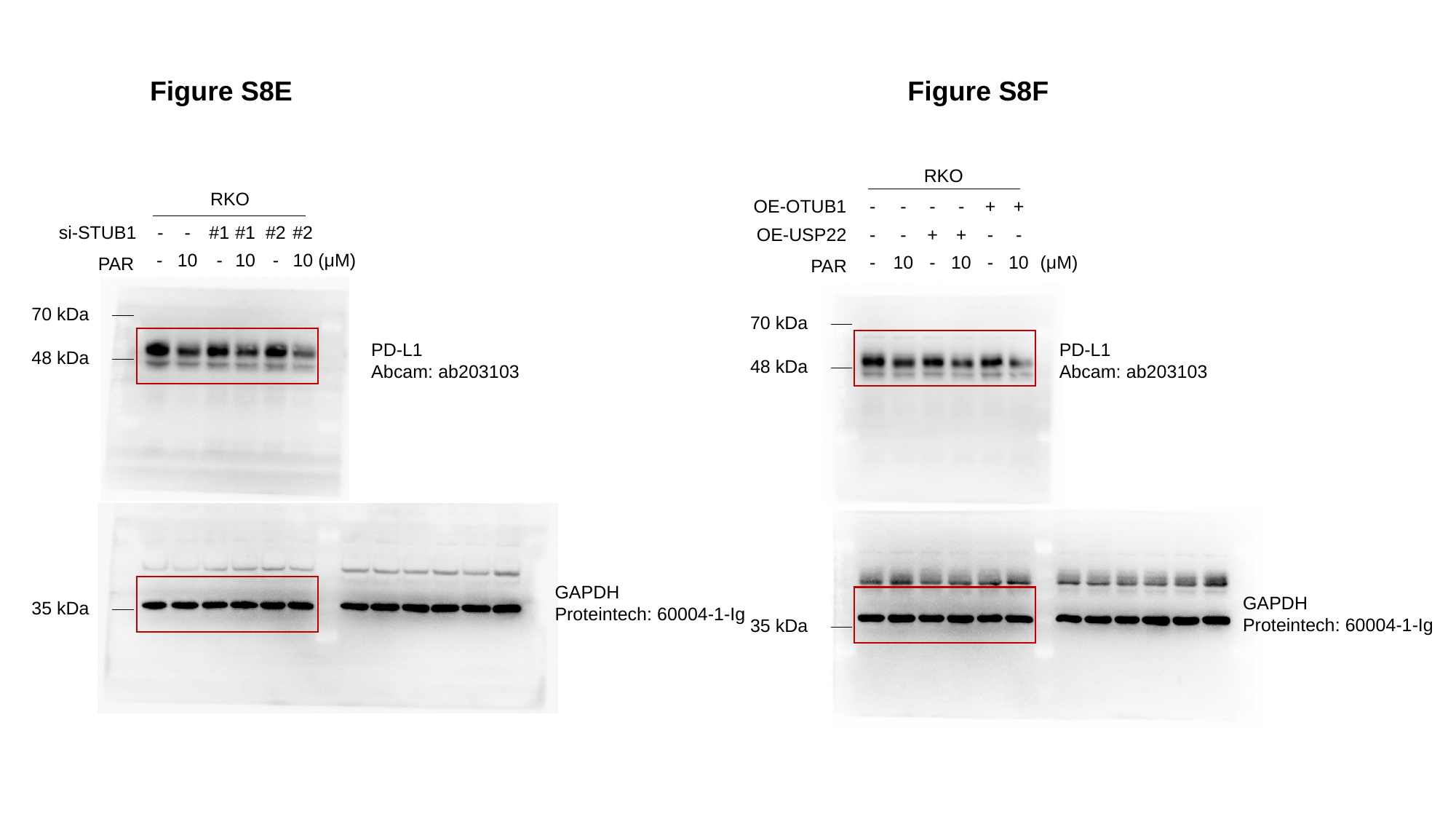

Figure S8E
Figure S8F
RKO
RKO
OE-OTUB1
-
-
-
-
+
+
si-STUB1
-
-
#1
#1
#2
#2
OE-USP22
-
-
+
+
-
-
-
10
-
10
-
10
(μM)
-
10
-
10
-
10
(μM)
PAR
PAR
70 kDa
70 kDa
PD-L1
Abcam: ab203103
PD-L1
Abcam: ab203103
48 kDa
48 kDa
GAPDH
Proteintech: 60004-1-Ig
GAPDH
Proteintech: 60004-1-Ig
35 kDa
35 kDa
